# Supplementary material for: Spatial epidemiology of gestational age and birth weight in Switzerland: census-based linkage study
Source: BMJ Open. 2019 Oct 30;9(10):e027834. doi: 10.1136/bmjopen-2018-027834 (PMC6830696; doi:10.1136/bmjopen-2018-027834)

Supplementary Table S1. Number of live births, mean gestational age and mean birth weight by maternal nationality in the eligible population (N = 315'177); 276 days correspond to $39^{3/7}$ weeks.

Nationality	N	Gestational age (days) Mean (SD)	Birth weight (g) Mean (SD)
**Switzerland**	194,570	276 (12)	3322 (511)
**Southern Europe**			
**Andorra**	1	279 (-)	3080 (-)
**Italy**	8337	275 (12)	3271 (496)
**Malta**	13	273 (8)	3188 (427)
**Portugal**	12,368	276 (12)	3235 (493)
**San Marino**	2	274 (1.4)	3485 (120)
**Spain**	2864	276 (12)	3263 (488)
**Western Europe**			
**Austria**	1555	275 (14)	3328 (528)
**Belgium**	583	276 (12)	3357 (482)
**Germany**	16,736	276 (13)	3369 (517)
**France**	6173	276 (12)	3294 (505)
**Lichtenstein**	100	275 (11)	3369 (488)
**Luxembourg**	55	276 (19)	3396 (636)
**Netherlands**	803	276 (12)	3377 (529)
**Northern Europe**			
**Denmark**	271	276 (13)	3383 (511)
**Estonia**	81	279 (7)	3601 (466)
**Finland**	312	276 (11)	3465 (523)
**Ireland**	212	276 (16)	3446 (548)
**Iceland**	31	272 (25)	3180 (775)
**Latvia**	187	279 (9)	3493 (434)
**Lithuania**	152	277 (13)	3450 (535)
**Norway**	110	275 (11)	3390 (525)
**Sweden**	571	276 (12)	3422 (470)
**UK**	1768	276 (13)	3397 (513)
**Eastern Europe**			
**Czech Republic**	623	275 (12)	3339 (499)
**Hungary**	913	275 (13)	3341 (512)
**Poland**	1778	276 (12)	3399 (497)
**Slovakia**	1068	276 (12)	3348 (509)
**Albania**	209	276 (12)	3406 (476)
**Bosnia & Herzegovina**	1952	276 (12)	3466 (492)
**Croatia**	1582	276 (12)	3448 (540)
**Kosovo**	10,278	276 (13)	3421 (530)
**Macedonia**	5842	276 (13)	3392 (514)
**Montenegro**	212	276 (10)	3416 (466)
**Serbia**	5195	276 (13)	3400 (536)
**Serbia & Montenegro**	10	277 (8)	3637 (250)
**Slovenia**	163	275 (15)	3366 (589)
**Cyprus**	15	278 (8)	3411 (525)
**Bulgaria**	406	273 (15)	3291 (559)
**Greece**	375	274 (13)	3317 (516)
**Romania**	971	274 (14)	3284 (537)
**Turkey**	4441	275 (13)	3347 (523)
**Belarus**	172	277 (13)	3385 (508)
**Moldova**	135	276 (10)	3496 (515)
**Russia**	1567	277 (12)	3427 (513)
**Ukraine**	855	277 (11)	3412 (473)
**Other (non-Europe)**			
6 most numerous: **Eritrea**	2600	279 (14)	3380 (528)
**Brazil**	2381	274 (12)	3312 (498)
**Sri Lanka**	1391	273 (14)	3158 (553)
**USA**	1291	276 (14)	3378 (532)
**China**	1293	276 (13)	3425 (541)
**Morocco**	1159	276 (14)	3378 (536)
. . .			
**Total**	315,177	276 (12)	3328 (515)

Supplementary Table S2. Comparison of results from fully adjusted model (model 3) accounting and not accounting for spatial autocorrelation. Based on complete-case population (*N* = 81,968).

	Accounting for spatial autocorrelation		Not accounting for spatial autocorrelation	
	**Gestational age (days) Absolute differences (95% CI)**	**Birth weight (g) * Relative differences (95% CI)**	**Gestational age (days) Absolute differences (95% CI)**	**Birth weight (g) * Relative differences (95% CI)**
Intercept	277.9 (277.6 to 278.1)	3293 (3163 to 3427)	277.9 (277.7 to 278.2)	3298 (3180 to 3420) &
**Sex**				
¶Female	0		0	1
Male	-0.62 (-0.78, -0.46)	1.048 (1.046, 1.049)	-0.63 (-0.79, -0.47)	1.048 (1.046, 1.049)
**Birth rank**				
1¶	0		0	1
2	-0.34 (-0.52, -0.17)	1.039 (1.037, 1.041)	-0.34 (-0.52, -0.16)	1.039 (1.037, 1.041)
3	-0.17 (-0.45, 0.10)	1.054 (1.051, 1.056)	-0.16 (-0.44, 0.11)	1.054 (1.051, 1.057)
≥ 4	0.23 (-0.26, 0.71)	1.065 (1.059, 1.070)	0.24 (-0.25, 0.72)	1.065 (1.059, 1.070)
**Maternal age (yrs)‡**				
≥ 20-30¶	0		0	1
≥ 30-40 yrs (per 5 yrs)	-0.92 (-1.06, -0.77)	0.998 (0.996, 1.000)	-0.93 (-1.07, -0.78)	0.998 (0.997, 1.000)
≥ 40 yrs (per 5 yrs)	-3.48 (-4.30, -2.66)	0.998 (0.990, 1.006)	-3.46 (-4.29, -2.63)	0.998 (0.990, 1.006)
**Civil status◊**				
Married¶	0		0	1
Not married	0.15 (-0.08, 0.38)	0.993 (0.990, 0.995)	0.15 (-0.08, 0.38)	0.993 (0.990, 0.995)
**Nationality mother**				
Switzerland¶	0		0	1
S Europe	0.38 (0.00, 0.77)	0.995 (0.992, 0.999)	0.39 (0.00, 0.78)	0.995 (0.991, 0.999)
W Europe	-0.08 (-0.42, 0.25)	1.007 (1.003, 1.010)	-0.08 (-0.43, 0.26)	1.007 (1.004, 1.011)
N Europe	0.30 (-0.56, 1.17)	1.022 (1.013, 1.031)	0.30 (-0.57, 1.17)	1.022 (1.013, 1.031)
E Europe	0.33 (-0.01, 0.67)	1.017 (1.013, 1.021)	0.33 (-0.01, 0.68)	1.017 (1.014, 1.021)
Other	-0.66 (-1.03, -0.29)	1.012 (1.008, 1.015)	-0.67 (-1.05, -0.30)	1.012 (1.008, 1.016)
**Nationality father**				
Switzerland¶	0		0	1
S Europe	-0.28 (-0.61, 0.06)	0.992 (0.989, 0.996)	-0.28 (-0.62, 0.06)	0.993 (0.989, 0.996)
W Europe	0.30 (-0.03, 0.64)	1.006 (1.002, 1.009)	0.30 (-0.04, 0.63)	1.006 (1.003, 1.010)
N Europe	-0.21 (-1.06, 0.63)	1.011 (1.002, 1.019)	-0.24 (-1.09, 0.62)	1.011 (1.003, 1.020)
E Europe	-0.02 (-0.38, 0.35)	1.011 (1.007, 1.015)	-0.01 (-0.38, 0.36)	1.011 (1.008, 1.015)
Other	0.49 (0.06, 0.91)	0.991 (0.987, 0.996)	0.48 (0.05, 0.90)	0.992 (0.987, 0.996)
**Education mother**				
Tertiary¶			0	1
Secondary	-0.56 (-0.75, -0.37)	0.997 (0.995, 0.999)	-0.55 (-0.74, -0.36)	0.996 (0.995, 0.998)
Compulsory	-0.92 (-1.23, -0.60)	0.993 (0.990, 0.997)	-0.90 (-1.22, -0.58)	0.993 (0.990, 0.996)
**Education father**				
Tertiary¶			0	1
Secondary	-0.16 (-0.35, 0.03)	0.997 (0.995, 0.999)	-0.16 (-0.35, 0.03)	0.996 (0.994, 0.998)
Compulsory	-0.25 (-0.57, 0.07)	0.997 (0.994, 1.001)	-0.25 (-0.58, 0.07)	0.997 (0.994, 1.000)
**Altitude (m)**				
500¶	0		0	1
per 500 m increase	-0.05 (-0.33, 0.24)	0.991 (0.987, 0.994)	0.03 (-0.24, 0.30)	0.989 (0.987, 0.992)
**Urbanization**				
Urban¶	0		0	1
Peri-urban	-0.54 (-0.75, -0.33)	1.001 (0.998, 1.004)	-0.59 (-0.82, -0.36)	1.003 (1.000, 1.005)
Rural	-0.25 (-0.50, 0.00)	1.003 (0.999, 1.006)	-0.29 (-0.55, -0.02)	1.003 (1.001, 1.006)
**Language region**				
German¶	0		0	1
French	-0.33 (-0.75, 0.09)	0.991 (0.983, 0.998)	-0.66 (-0.88, -0.44)	0.988 (0.985, 0.990)
Italian	-1.10 (-1.50, -0.70)	0.984 (0.978, 0.989)	-1.11 (-1.55, -0.68)	0.983 (0.979, 0.987)

*Birth weight was modelled on a log scale, which results in multiplicative effects. The model for birth weight was additionally adjusted for gestational age by a cubic spline function with knots at weeks 25, 30 and 35.

& In the model for BW, the intercept corresponds to an estimated mean birth weight (g) for a singleton girl born at gestational age 40 weeks as the first child (rank 1) in a German-speaking, urban region of elevation 500 m, whose mother is 20-30 years old at birth and married, and both parents have Swiss nationality and tertiary education.

¶ Reference category

‡ Age modelled by a piece-wise linear function: constant at reference range ≥20-30, and separate slopes for age <20, ≥30-40, and ≥40. For ages ≥40, the total estimated effect is hence addition of 10-year effect in age group ≥30-40 plus the corresponding effect in age-group ≥40.

† Percentage of regional variance explained by model predictors, i.e. percent reduction in variance of random effects (σ^2) when compared to model with no predictors (model 0).

Supplementary Table S3. Comparison of results from model (model 3) for birth weight, adjusted and not adjusted for gestational age.

	Birth weight - Model 3 without Gestational Age		Birth weight - Model 3 with Gestational Age*	
	Relative differences (95% CI)		Relative differences (95% CI)	
	Eligible population	Complete case population	Eligible population	Complete case population
**Intercept**	3188 (3181 to 3195)	3209 (3196 to 3222)	3278 (3218 to 3339)[&]	3298 (3180 to 3420)[&]
**Sex**				
¶Female	1	1	1	1
Male	1.04 (1.038, 1.041)	1.041 (1.039, 1.044)	1.045 (1.044 to 1.046)	1.048 (1.046, 1.049)
**Rank**				
1¶	1	1	1	1
2	1.039 (1.038, 1.041)	1.041 (1.038, 1.044)	1.038 (1.037, 1.039)	1.039 (1.037, 1.041)
3	1.052 (1.049, 1.054)	1.058 (1.054, 1.063)	1.050 (1.048, 1.051)	1.054 (1.051, 1.057)
≥ 4	1.060 (1.056, 1.064)	1.071 (1.063, 1.079)	1.058 (1.056, 1.061)	1.065 (1.059, 1.070)
**Age mother (yrs)‡**				
< 20yrs (per 5 yrs decr.)	0.956 (0.935, 0.978)	-	1.002 (0.987, 1.017)	-
≥ 20-30 yrs¶	1	1	1	1
≥ 30-40 yrs (per 5 yrs)	0.991 (0.990, 0.992)	0.990 (0.988, 0.993)	1.000 (1.000, 1.001)	0.998 (0.997, 1.000)
≥ 40 yrs (per 5 yrs)	0.973 (0.967, 0.979)	0.967 (0.955, 0.979)	0.998 (0.994, 1.003)	0.998 (0.990, 1.006)
**Civil status⁰**				
Married¶	1	1	1	1
Not married	0.989 (0.987, 0.990)	0.992 (0.989, 0.996)	0.990 (0.989, 0.991)	0.993 (0.99, 0.995)
**Nationality mother**				
Switzerland¶	1	1	1	1
S Europe	0.996 (0.993, 0.999)	0.999 (0.993, 1.005)	0.994 (0.992, 0.996)	0.995 (0.991, 0.999)
W Europe	1.010 (1.007, 1.013)	1.006 (1.001, 1.011)	1.008 (1.006, 1.010)	1.007 (1.004, 1.011)
N Europe	1.027 (1.020, 1.034)	1.024 (1.010, 1.037)	1.025 (1.020, 1.029)	1.022 (1.013, 1.031)
E Europe	1.014 (1.011, 1.016)	1.020 (1.015, 1.026)	1.013 (1.011, 1.014)	1.017 (1.014, 1.021)
Other	1.003 (1.000, 1.006)	1.005 (0.999, 1.010)	1.007 (1.005, 1.008)	1.012 (1.008, 1.016)
**Nationality father**				
Switzerland¶	1	1	1	1
S Europe	0.988 (0.985, 0.991)	0.991 (0.986, 0.996)	0.991 (0.990, 0.993)	0.993 (0.989, 0.996)
W Europe	1.008 (1.005, 1.011)	1.009 (1.004, 1.014)	1.008 (1.006, 1.009)	1.006 (1.003, 1.010)
N Europe	1.017 (1.010, 1.024)	1.007 (0.994, 1.019)	1.013 (1.009, 1.017)	1.011 (1.003, 1.020)
E Europe	1.003 (1.000, 1.006)	1.010 (1.004, 1.015)	1.009 (1.007, 1.010)	1.011 (1.008, 1.015)
Other	0.990 (0.987, 0.993)	0.997 (0.990, 1.003)	0.992 (0.991, 0.994)	0.992 (0.987, 0.996)
missing	0.933 (0.928, 0.938)	-	0.989 (0.985, 0.992)	-
**Education mother**				
Tertiary¶	-	1	-	1
Secondary	-	0.992 (0.989, 0.995)	-	0.996 (0.995, 0.998)
Compulsory	-	0.984 (0.979, 0.989)	-	0.993 (0.990, 0.996)
**Education father**				
Tertiary¶	-	1	-	1
Secondary	-	0.995 (0.992, 0.997)	-	0.996 (0.994, 0.998)
Compulsory	-	0.995 (0.990, 1.000)	-	0.997 (0.994, 1.000)
**Altitude (m)**				
500 m¶	1	1	1	1
per 500 m increase	0.990 (0.988, 0.992)	0.989 (0.985, 0.993)	0.989 (0.988, 0.991)	0.989 (0.987, 0.992)
**Urbanization**				
Urban¶	1	1	1	1
Peri-urban	0.998 (0.996, 1.000)	0.998 (0.995, 1.002)	1.001 (1.000, 1.002)	1.003 (1.000, 1.005)
Rural	0.999 (0.996, 1.001)	1.001 (0.997, 1.005)	1.000 (0.998, 1.001)	1.003 (1.001, 1.006)
**Language region**				
ᴿGerman¶	1	1	1	1
French	0.985 (0.983, 0.987)	0.983 (0.980, 0.986)	0.989 (0.987, 0.990)	0.988 (0.985, 0.990)
Italian	0.977 (0.973, 0.981)	0.976 (0.970, 0.982)	0.982 (0.980, 0.985)	0.983 (0.979, 0.987)
**% variation explained**				
Model 3	77%	76%	87%	88%
Model 2	25%	27%	52%	56%
Model 1	2%	5%	31%	37%

*Birth weight was modelled on a log scale, which results in multiplicative effects.
¶ Reference category
‡ Age modelled by a piece-wise linear function: constant at reference range ≥20-30, and separate slopes for age <20, ≥30-40, and ≥40. For ages ≥40, the total estimated effect is hence addition of 10-year effect in age group ≥30-40 plus the corresponding effect in age-group ≥40.
† Percentage of regional variance explained by model predictors, i.e. percent reduction in variance of random effects (σ^2) when compared to model with no predictors (model 0).

Supplementary Table S4. Comparison of results from fully adjusted model without and with additionally including Swiss index of socio-economic position (SEP). Based on eligible population (*N* = 315,177); 277 days correspond to $39^{4/7}$ weeks.

	Model 3 without SEP		Model 3 with SEP	
	Gestational age (days) Absolute differences (95% CI)	Birth weight (g) * Relative differences (95% CI)	Gestational age (days) Absolute differences (95% CI)	Birth weight (g) * Relative differences (95% CI)
**Intercept**	277.3 (277.2 to 277.5)	3278 (3218 to 3339)[&]	277.3 (277.2 to 277.5)	3278 (3218 to 3339)[&]
**Sex**				
[¶]Female	0	1	0	1
Male	-0.56 (-0.65, -0.48)	1.045 (1.044 to 1.046)	-0.56 (-0.65, -0.48)	1.045 (1.044 to 1.046)
**Birth rank**				
1[¶]	0	1	0	1
2	-0.39 (-0.49, -0.29)	1.038 (1.037, 1.039)	-0.39 (-0.49, -0.29)	1.038 (1.037, 1.039)
3	-0.37 (-0.52, -0.22)	1.050 (1.048, 1.051)	-0.37 (-0.52, -0.22)	1.050 (1.048, 1.051)
≥ 4	-0.24 (-0.50, 0.02)	1.058 (1.056, 1.061)	-0.24 (-0.49, 0.02)	1.058 (1.056, 1.061)
**Age mother (yrs)[‡]**				
< 20 (per 5 yrs decr.)	-4.10 (-5.59, -2.61)	1.002 (0.987, 1.017)	-4.10 (-5.59, -2.61)	1.002 (0.987, 1.017)
20 - <30[¶]	0	1	0	1
≥ 30-40 (per 5 yrs)	-0.99 (-1.06, -0.91)	1.000 (1.000, 1.001)	-0.99 (-1.07, -0.92)	1.000 (1.000, 1.001)
≥ 40 (per 5 yrs)	-2.93 (-3.36, -2.50)	0.998 (0.994, 1.003)	-2.92 (-3.35, -2.50)	0.998 (0.994, 1.003)
**Civil status[◊]**				
Married	0	1	0	1
Not married	-0.01 (-0.13, 0.10)	0.990 (0.989, 0.991)	-0.01 (-0.13, 0.10)	0.990 (0.989, 0.991)
**Nationality mother**				
Switzerland[¶]	0	1	0	1
S Europe	0.20 (-0.01, 0.40)	0.994 (0.992, 0.996)	0.20 (0.00, 0.41)	0.994 (0.992, 0.996)
W Europe	0.20 (0.02, 0.38)	1.008 (1.006, 1.010)	0.20 (0.02, 0.37)	1.008 (1.006, 1.010)
N Europe	0.37 (-0.07, 0.81)	1.025 (1.020, 1.029)	0.36 (-0.08, 0.80)	1.025 (1.020, 1.029)
E Europe	0.21 (0.04, 0.38)	1.013 (1.011, 1.014)	0.22 (0.05, 0.39)	1.013 (1.011, 1.015)
Other	-0.32 (-0.49, -0.14)	1.007 (1.005, 1.008)	-0.31 (-0.48, -0.14)	1.007 (1.005, 1.008)
**Nationality father**				
Switzerland[¶]	0	1	0	1
S Europe	-0.46 (-0.64, -0.28)	0.991 (0.990, 0.993)	-0.45 (-0.63, -0.27)	0.992 (0.99, 0.993)
W Europe	0.07 (-0.11, 0.25)	1.008 (1.006, 1.009)	0.07 (-0.11, 0.25)	1.008 (1.006, 1.009)
N Europe	0.51 (0.08, 0.94)	1.013 (1.009, 1.017)	0.50 (0.07, 0.93)	1.013 (1.009, 1.017)
E Europe	-0.46 (-0.64, -0.28)	1.009 (1.007, 1.010)	-0.45 (-0.63, -0.27)	1.009 (1.007, 1.011)
Other	-0.02 (-0.22, 0.18)	0.992 (0.991, 0.994)	-0.01 (-0.21, 0.19)	0.993 (0.991, 0.995)
missing	-3.87 (-4.24, -3.50)	0.989 (0.985, 0.992)	-3.86 (-4.23, -3.49)	0.989 (0.985, 0.993)
**SEP index**				
1st quintile	-	-	-0.08 (-0.25, 0.08)	0.997 (0.996, 0.999)
2nd quintile	-	-	-0.09 (-0.24, 0.06)	0.998 (0.997, 1.000)
3rd quintile	-	-	-0.02 (-0.17, 0.13)	0.998 (0.997, 0.999)
4th quintile	-	-	0.02 (-0.12, 0.17)	1.000 (0.999, 1.002)
5th quintile[¶]	-	-	0	1
**Altitude (m)**				
500[¶]	0	1	0	1
per 500 m increase	0.07 (-0.09, 0.23)	0.989 (0.988, 0.991)	0.08 (-0.08, 0.23)	0.989 (0.988, 0.991)
**Urbanization**				
Urban[¶]	0	1	0	1
Peri-urban	-0.43 (-0.57, -0.28)	1.001 (1.000, 1.002)	-0.43 (-0.57, -0.28)	1.001 (1.000, 1.002)
Rural	-0.15 (-0.32, 0.02)	1.000 (0.998, 1.001)	-0.13 (-0.30, 0.04)	1.001 (0.999, 1.002)
**Language region**				
German[¶]	0	1	0	1
French	-0.62 (-0.77, -0.47)	0.989 (0.987, 0.990)	-0.61 (-0.76, -0.46)	0.989 (0.987, 0.990)
Italian	-0.94 (-1.26, -0.63)	0.982 (0.980, 0.985)	-0.93 (-1.24, -0.61)	0.982 (0.980, 0.985)
**% variation explained**				
Full model	31%	87%	31%	88%
Model without Lang. region	14%	66%	15%	68%

*Birth weight was modelled on a log scale, which results in multiplicative effects. The model for birth weight was additionally adjusted for gestational age by a cubic spline function with knots at weeks 25, 30 and 35.

[&] In the model for BW, the intercept corresponds to an estimated mean birth weight (g) for a singleton girl born at gestational age 40 weeks as the first child (rank 1) in a German-speaking, urban region of elevation 500m, whose mother is 20-30 years old at birth and married, and both parents have Swiss nationality and tertiary education.

[¶] Reference category

‡ Age modelled by a piece-wise linear function: constant at reference range ≥20-30, and separate slopes for age <20, ≥30-40, and ≥40.

◊ Married or in registered partnership / Not married: Single, widow, divorced or in dissolved partnership

† Percentage of regional variance explained by model predictors, i.e. percent reduction in variance of random effects (σ^2) when compared to model with no predictors (model 0).

Supplementary Figure S1. Selection of eligible and complete case study populations among live births in Switzerland 2011 to 2014.

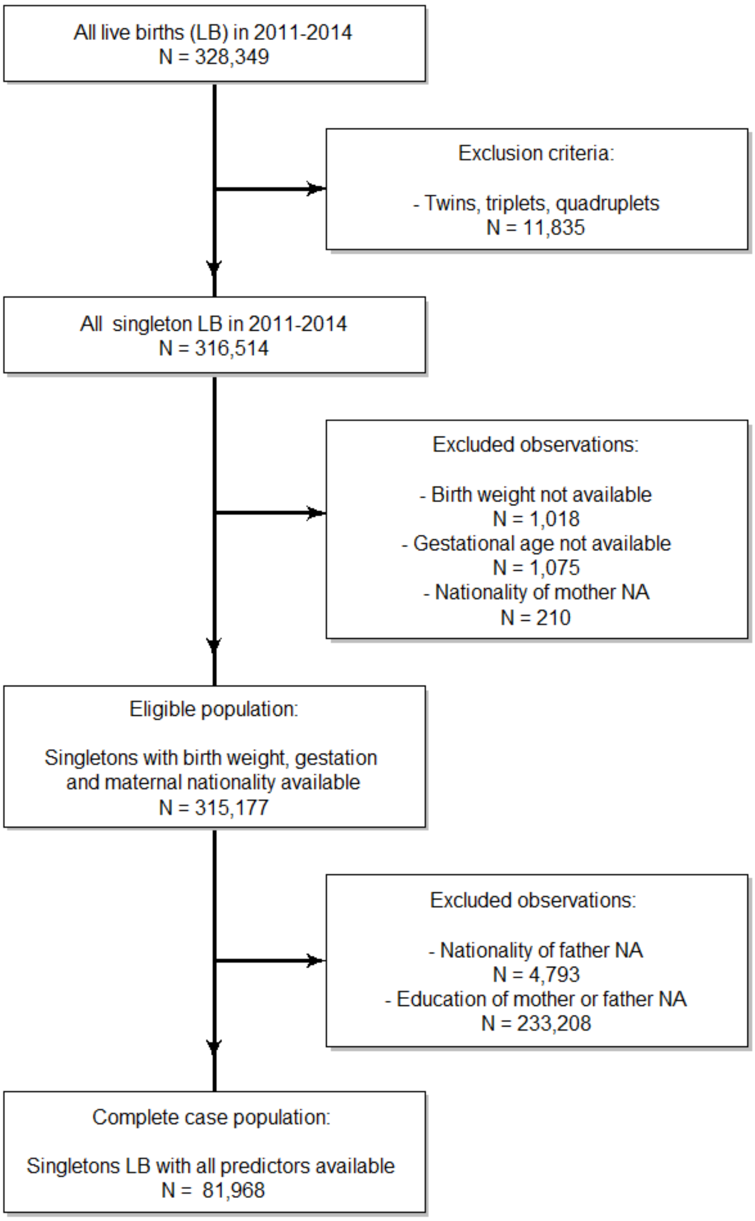

Supplementary Figure S2. Relationship between birth weight and gestational age at birth modelled by a cubic spline function. Separate fitted curves are shown for newborn girls and boys, with all other predictors corresponding to the reference categories shown in Table 2.

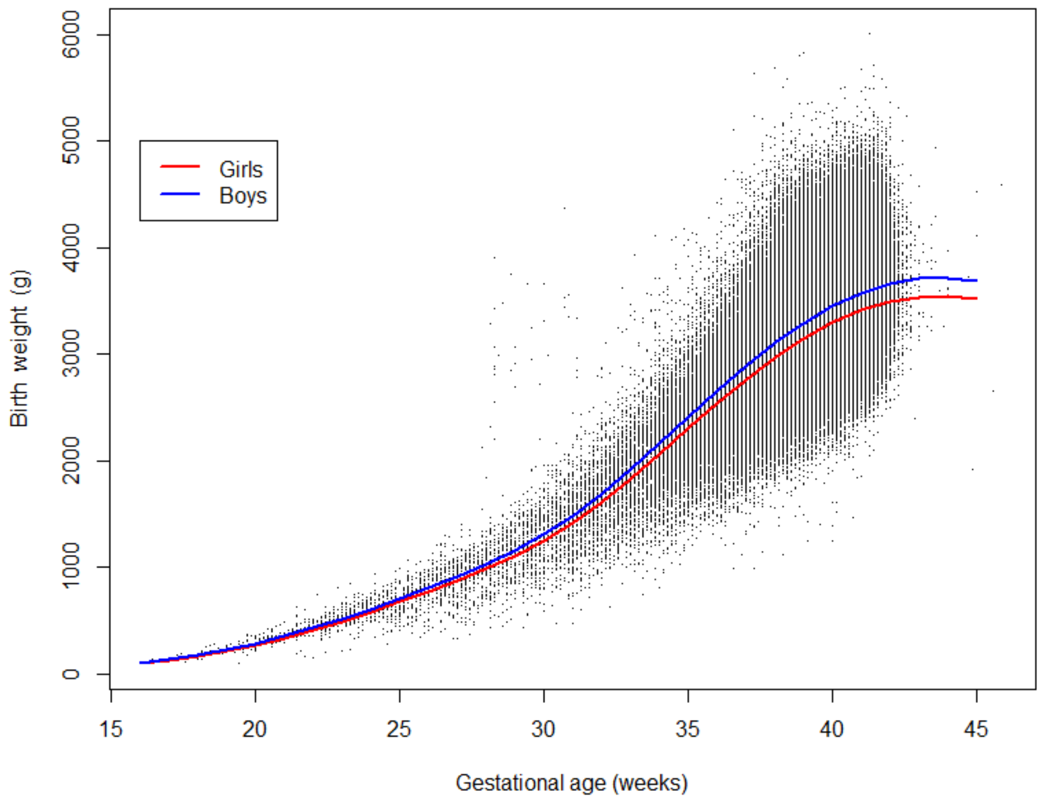

Supplementary Figure S3. Relationship between mean gestational age and proportion of preterm live births (<37 weeks) among eligible live births across 705 regions (upper panel) and between mean birth weight and proportion of low birth weight births (<2500g) (lower panel). Results from linear regression weighted by the number of live births in each region. Prediction intervals displayed for an average-size region (n=447). GA = gestational age; BW= birth weight; 276 days correspond to 39$^{3/7}$ weeks.

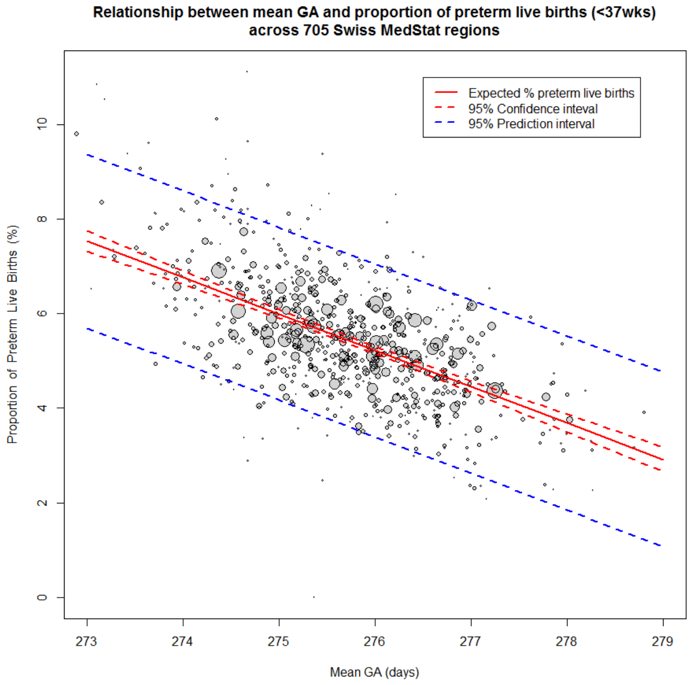

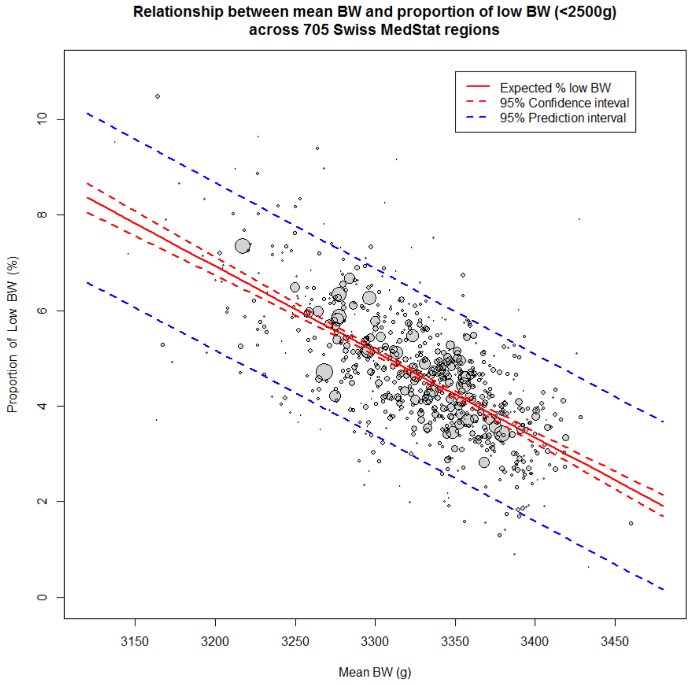

Supplement: Supplementary data [file bmjopen-2018-027834supp001.pdf]
