# Supplementary material for: Spatial epidemiology of gestational age and birth weight in Switzerland: census-based linkage study
Source: BMJ Open. 2019 Oct 30;9(10):e027834. doi: 10.1136/bmjopen-2018-027834 (PMC6830696; doi:10.1136/bmjopen-2018-027834)
Supplement: Author's manuscript [file bmjopen-2018-027834.draft_revisions.pdf]

BMJ Open is committed to open peer review. As part of this commitment we make the peer review history of every article we publish publicly available.

When an article is published we post the peer reviewers' comments and the authors' responses online. We also post the versions of the paper that were used during peer review. These are the versions that the peer review comments apply to.

The versions of the paper that follow are the versions that were submitted during the peer review process. They are not the versions of record or the final published versions. They should not be cited or distributed as the published version of this manuscript.

BMJ Open is an open access journal and the full, final, typeset and author-corrected version of record of the manuscript is available on our site with no access controls, subscription charges or pay-per-view fees (http://bmjopen.bmj.com).

If you have any questions on BMJ Open's open peer review process please email info.bmjopen@bmj.com

BMJ Open

Spatial epidemiology of gestational age and birth weight in Switzerland: Census-based linkage study

Journal:	*BMJ Open*
Manuscript ID	bmjopen-2018-027834
Article Type:	Research
Date Submitted by the Author:	19-Nov-2018
Complete List of Authors:	Skrivankova, Veronika; Universitat Bern Institut fur Sozial- und Praventivmedizin,<br>Zwahlen , Marcel; Universitat Bern Institut fur Sozial- und Praventivmedizin<br>Adams, Mark; University of Zurich, Department of Neonatology<br>Low, Nicola; University of Bern, Institute of Social and Preventive Medicine<br>Kuehni, Claudia; Universitat Bern Institut fur Sozial- und Praventivmedizin<br>Egger, Matthias; University of Bern, Institute of Social and Preventive Medicine
Keywords:	birth weight, gestational age, spatial epidemiology, Switzerland, socio-economic position, eduction

SCHOLARONE™
Manuscripts

Spatial epidemiology of gestational age and birth weight in Switzerland: Census-based linkage study

Veronika Skrivankova[1], Marcel Zwahlen[1], Mark Adams[2], Nicola Low[1], Claudia E Kuehni[1,3], Matthias Egger[1]

1) Institute of Social and Preventive Medicine (ISPM), University of Bern, Bern, Switzerland

2) Department of Neonatology, University Hospital Zurich, University of Zurich, Zurich, Switzerland

3) Pediatric Respiratory Medicine, Inselspital, Bern University Hospital, University of Bern, Switzerland

Correspondence to:

Professor Matthias Egger MD

Institute of Social and Preventive Medicine (ISPM), University of Bern

Mittelstrasse 43, CH-3012 Bern, Switzerland

matthias.egger@ispm.unibe.ch

Word counts: Abstract 197 words, strengths and limitations of the study 129 words, main text 3145 words, 43 references, 3 tables, 2 figures, supplementary materials with 3 tables and 3 figures.

For peer review only

ABSTRACT

Objectives To examine small-area variation in gestational age and birth weight in Switzerland.

Design Linkage of population census and survey data with Live Birth Register and smallarea analysis.

Setting: Resident population of Switzerland.

Participants: All 315,177 singleton live births recorded in the Swiss Live Birth Register 2011 to 2014.

Primary outcome measures: Gestational age and birth weight.

Results: Area-level averages of gestational age varied between 272-279 days, and between 3138-3467g for birth weight. The fully adjusted models explained 31% and 87% of spatial variation of gestational age and birth weight, respectively. Language region explained most of the variation, with shorter gestational age and lower birth weight in French- and Italian- than in German-speaking areas. Other variables explaining variation were, for gestational age, the level of urbanisation, the parents' nationality and missing father. For birth weight, they were gestational age, altitude, born out of wedlock, and parental nationality. In a subset of 69,463 live births with data on parental education, levels of education were only weakly associated with gestational age or birth weight.

Conclusions: In Switzerland, small area variation in birth weight is largely explained, and variation in gestational age partially explained by geocultural, socio-demographic and pregnancy factors.

For peer review only - http://bmjopen.bmj.com/site/about/guidelines.xhtml

Strengths and limitations of this study

- This study was based on a large sample with national coverage, with data on neonatal and pregnancy-related predictors of gestational age and birth weight, and precise spatial data.
- No data were available on the mode of delivery, maternal smoking, mothers' weight and height or gestational diabetes.
- The fully adjusted model explained about 80% of the regional variation in birth weight and about 40% of the variation in gestational age.
- Language region, a proxy for cultural, social and behavioural factors, was a strong explanatory factor, with lower birth weight and shorter gestation in the French and Italian compared to the German language region.
- Unknown father was associated with shorter gestation and lower birth weight, indicating that children not recognised by their fathers may be at higher risk of poor outcomes.

For peer review only

For peer review only - http://bmjopen.bmj.com/site/about/guidelines.xhtml

INTRODUCTION

Gestational age and birth weight are important indicators of prenatal development and predictors of infant morbidity, mortality and long-term health [1–4]. An understanding of geographic differences and their determinants can help to develop policies that reduce health inequalities across population groups and regions [1–4]. Many genetic, physiological, pregnancy-related, socio-economic, lifestyle and environmental factors have been reported to influence gestational age and birth weight [5–8]. Some of these factors tend to cluster in space and regional differences in health outcomes may hence be partially explained by the spatial distribution of their predictors. Importantly, both individual-level factors and the social and environmental characteristics of communities and neighbourhoods may contribute to regional differences [9,10].

Variation across small areas in pregnancy outcomes have not been studied widely. In Scotland, small area crime rates were associated with lower birth weight and with the risk of both small for gestational age babies and preterm birth [11]. A study at county level in Georgia and South Carolina in the United States showed that the proportion of African Americans was associated with low birth weight, whereas higher income was associated with higher birth weight [12]. Similarly, neighbourhood racial composition contributed to variation in low birth weight in New York State [13]. Other small-area analyses have examined associations between birth outcomes and air pollution [14,15]. To our knowledge, few small-area analyses have considered gestational age.

In Switzerland, studies of pregnancy outcomes have focused on specific groups such as migrants or HIV-infected women [16,17], but have not examined geographic variations. The Federal Office of Statistics publishes routine statistics from the Live Birth Register, which does not include geographic information [18]. The objectives of this study were to conduct a nationwide analysis of spatial variation in gestational age and birth weight, and to assess how much small-area variation was explained by available data about neonatal and pregnancy-related variables, parental characteristics and geographical variables.

For peer review only - http://bmjopen.bmj.com/site/about/guidelines.xhtml

METHODS

Data sources

We used deterministic methods to link three data sources using encrypted national identification numbers: the Live Birth Register, the Swiss National Cohort and the Structural Surveys. Registration of live births is compulsory by law in Switzerland coverage is near 100%. The Swiss National Cohort (SNC) is a long-term, national study of mortality in Switzerland [19,20], linking census and mortality records. The 1990 and 2000 censuses were the last house-to-house censuses with coverage of the entire Swiss population. From 2010 onwards, the national census was replaced by a national population register and annual postal survey of the resident population, known as Structural Surveys [21]. Each structural Survey includes a random sample of around 300,000 people aged 15 years or older; for example, in 2010, it included 317,221 persons [21]. The reference is the entire Swiss resident population and the reference day 31 December.

Variables and definitions

We defined three sets of variables. The first set, neonatal and pregnancy-related variables come from the Live Birth Register; date of birth, birth weight, gestational age, sex and birth rank. Birth weight is measured after initial mother-child bonding, usually by the midwife using a calibrated hospital scale. Gestational age is based on the last menstrual period, with or without additional information from ultrasound scans. Birth rank was classified as 1, 2, 3 and ≥4 live births, including the current birth. Birth rank is only available if the mother was married at the time of birth, and it is counted only within the current marriage. The second set includes parental variables. The Structural Surveys provide information about the highest level of completed maternal and paternal education, classified as ‘tertiary’, ‘secondary’, or ‘compulsory or less’. The Swiss National Cohort provides data about parental nationality categorised as ‘Swiss’, ‘Southern Europe’, ‘Western Europe’, ‘Northern Europe’, ‘Eastern Europe’, ‘Other’ (non-European), or missing (supplementary Table S1 gives the full list of countries). The third set, geographical variables comes from the Swiss National Cohort. Each live birth was assigned an altitude and one of 705 statistical areas [22], based on the geocode of place of residence of the mother at the time of birth. Language regions are ‘German’, ‘French’ and ‘Italian’, and the level of urbanisation was defined using standard definitions of ‘urban’, ‘peri-urban’ and ‘rural’.

For peer review only - http://bmjopen.bmj.com/site/about/guidelines.xhtml

Study populations and outcomes

All singleton live births recorded in the Live Birth Register from 1 January 2011 to 31 December 2014 were eligible. Gestational age at birth and birth weight were the outcomes of interest. For each outcome, two datasets were analysed: the first, larger dataset consisted of all eligible births with complete data on gestational age, birth weight and nationality of the mother. The second was the complete case population containing eligible live births with available data on all variables, including parental education. The second dataset included married mothers only who delivered at age 20 years or older because the birth rank is available for married women only, and education is incomplete below age 20 years.

Statistical and spatial analyses

We fitted linear mixed-effect models (LMEM) to examine the associations between the two outcomes and the neonatal and pregnancy, parental and environmental factors. In the model for birth weight, we log-transformed the outcome and used a cubic spline function with three knots at weeks 25, 30 and 35 to capture the relationship between gestational age and log birth weight. Log transforming the birth weight results in a multiplicative model. Except for gestational age, maternal age and altitude, all predictors were modelled categorically. Maternal age was modelled by a piece-wise linear function, with age group 20 to 30 years as the reference group and separate linear trends for age groups 30-40 years, over 40 years and less than 20 years. Altitude was centred at 500 m and modelled linearly. The random effects in the mixed-effect model captured area-level differences between observed and expected mean outcome, based on the 705 statistical areas [22]. In the main analysis, we fitted four models to the complete-case dataset: Model 0 contained no explanatory variables. Model 1 included birth and pregnancy-related variables: sex, birth rank and gestational age (for the analysis of birth weight). Model 2 additionally included age of the mother, parental education and nationality. Model 3 additionally included geographical variables: altitude, degree of urbanisation and language region.

We displayed mean gestational age and birth weight at area-level on maps and assessed to what extent spatial variation was accounted for by the explanatory variables. Values were categorised into seven intervals symmetric around the mean and color-coded. Spatial autocorrelation of the gestational age and birth weight across regions was tested by global and local Moran's I tests [23]. The global Moran test summarises overall spatial autocorrelation and the local test identifies areas that are correlated with neighbouring

For peer review only - http://bmjopen.bmj.com/site/about/guidelines.xhtml

areas. In the presence of spatial autocorrelation, model estimates are at risk of bias if the autocorrelation is not taken into account.

In a sensitivity analysis, we accounted for spatial autocorrelation using the Besag-York-Mollier (BYM) model [24] using uninformative gamma-distributed (1, 0.005) priors. The calculations were carried out using the Integrated Nested Laplace Approximation (INLA) approach [25]. Similar results from models with and without the spatial component indicate low bias. Finally, we repeated analyses of birth weight without adjusting for gestational age. All analyses and maps were done in R 3.3.2 [26] using packages lme4, maptools, sp, spdep, rgdal, INLA, GISTools, rgeos, raster and ggplot2.

Patient and public involvement

This analysis was based on routine registry data and no patients were involved in developing the research question, outcome measures and overall design of the study. Due to the anonymous nature of the data, we were unable to disseminate the results of the research directly to study participants.

For peer review only - http://bmjopen.bmj.com/site/about/guidelines.xhtml

RESULTS

Characteristics of study populations

A total of 328,349 live births were recorded in Switzerland between 1 January 2011 and 31 December 2014. We excluded non-singleton live births (n=11,835) and those with missing gestational age, birth weight or maternal nationality. The eligible study population therefore included 315,177 singleton live births. The complete case population consisted of 69,463 singleton live births with values available for all predictors including parental education, for which complete data were only available in the Structural Surveys (supplementary Figure S1).

Table 1 shows the distributions of predictors and outcomes in the two study populations. Data about the nationality of fathers was missing for 1.5% of eligible live births. In almost all of these cases, information about the father was missing completely, indicating that the father is unknown to the authorities. Apart from missing data, the distributions of most variables were similar between the two nested datasets. By design, the complete case population included married mothers only. The proportion of Swiss mothers and fathers was higher in the complete case population than in the eligible population. Birth at full term was defined as between 39 and 41 weeks of gestation (273 to 287 days). The mean gestational age in the eligible population was 276 days (SD 12) and the mean birth weight 3328 g (SD 515). The corresponding figures in the complete case population were 276 days (SD 12) and 3349 g (SD 501).

Maps of gestational age and birth weight

Figure 1 presents maps of Switzerland with crude average gestational age and birth weight across the 705 areas. For both outcomes, the maps are broadly similar between the eligible and complete case populations. For gestational age, area-level averages for the eligible population vary between 272 and 279 days. For the complete case population variation was greater, from 265 to 281 days, as expected for a smaller sample. The map shows shorter gestation in the Western, North Western region and Southern (Canton of Ticino) regions of Switzerland, with a patchy pattern in the densely populated areas between the Alps (across the centre) and Jura mountain ranges (to the North West). For birth weight, area-level averages vary between 3138 and 3467g for the eligible population and between 3020 and 3597g for the complete case population. The maps for birth weight show lower birth weights

For peer review only - http://bmjopen.bmj.com/site/about/guidelines.xhtml

in the Western and Southern regions of the country. The French and Italian-speaking regions are in the West and South of Switzerland, with the remainder being German-speaking.

Multivariable analyses

Table 2 shows associations of area-level mean gestational age at birth and mean birth weight with pregnancy, parental and environmental factors from the fully adjusted linear mixed-effects models (model 3). For gestational age, the largest differences are observed across categories of maternal age at birth, with pregnancies in mothers aged 40 years or older, and below 20 years about 3 days shorter than in mothers aged 20 to 30 years in both the eligible and the complete case populations. Of note, compared with Swiss fathers, pregnancies were about 4 days shorter if the nationality of the father was missing. Smaller differences in gestational age were observed across categories of sex, birth rank, nationality of the mother, urbanisation and between language regions (Table 2). In the complete case population, lower levels of education were associated with shorter pregnancies. Gestational age at birth was not associated with altitude.

Supplementary Figure S2 shows the relationship between gestational age and birth weight separately for male and female newborns. Male newborns were about 5% heavier than female newborns and birth weight increased with birth order (Table 2). In contrast to gestational age, mother's age was not associated with birth weight. Babies born to mothers or fathers from Northern or Eastern Europe were slightly heavier than babies born to Swiss mothers; birth weights were lowest for babies of fathers with missing nationality. Birth weight slightly decreased with increasing parental educational attainment. Babies born in the French and Italian-speaking regions were lighter than babies born in the German-speaking Switzerland. Finally, birth weight decreased with increasing altitude of residence.

Proportion of spatial variation explained

The fully adjusted model (model 3) for gestational age explained 31% and 41% of the spatial variation across the 705 areas for eligible and complete case populations, respectively. The corresponding figures for birth weight were 87% and 82%. When assessing each factor separately (Table 3), language region alone explained most of the spatial variation for both outcomes. For gestational age, level of urbanisation of the mother's place of residence also explained part of the variation. Factors that also contributed to explaining the spatial variation in birth weight were gestational age, parental nationalities, altitude at the mother's

For peer review only - http://bmjopen.bmj.com/site/about/guidelines.xhtml

place of residence and birth order. Figure 2 illustrates the reduction in the spatial variation of gestational age and birth weight with maps, when moving from model 0 (0% reduction) to models 1, 2 and 3, based on the complete case population.

Spatial autocorrelation and sensitivity analyses

For gestational age, the global Moran's I statistic, based on the complete case dataset and model 0, was I=0.19, with $P<10^{-13}$. After adjusting for all the predictors in model 3 there was still some residual autocorrelation (*I*=0.09, P=0.0001). For birth weight, the corresponding Moran's I statistic was I=0.26, with $P<10^{-15}$. After adjusting for all predictors in model 3 there was little residual autocorrelation (I=0.04, P=0.07). Supplementary Table S2 compares the results from model 3 accounting and not accounting for spatial autocorrelation. The results are similar and the potential bias from residual spatial autocorrelation is therefore unlikely to be a major issue. Repeating analyses of birth weight without adjusting for gestational age produced generally similar coefficients (supplementary Table S3). Associations with maternal age, maternal education and language regions were slightly stronger in model 3 without adjustment for gestational age, possibly because some of their effect was mediated by gestational age. Model 3 without gestational age explained 77% of the spatial variation both in the eligible and complete case populations.

For peer review only

For peer review only - http://bmjopen.bmj.com/site/about/guidelines.xhtml

DISCUSSION

Our study assessed factors associated with gestational age and birth weight in Switzerland and their contribution to spatial variation, based on routinely collected data. Gestational age at birth was strongly associated with maternal age, missing information on the father and language region. Birth weight was associated with sex, birth rank, missing information on the father, parental education, altitude and language region. There was substantial regional variation and spatial autocorrelation across regions. The variables included in the fully adjusted model explained about 80% of the regional variation in birth weight and about 40% of the regional variation in gestational age. Strengths of this study include a large sample with national coverage of the Swiss resident population, as well as the availability of data on several relevant predictors, either on all births or on a large random sample of eligible births. Precise spatial data and spatial statistics allowed us to assess the proportion of area-level variation explained, spatial autocorrelation and gauge the likelihood of bias due to residual autocorrelation.

This study found important spatial variation in both gestational age and birth weight in Switzerland. Language region in Switzerland was the single factor that explained the greatest proportion of spatial variation in gestational age and birth weight. In the French and Italian speaking regions, gestational age was shorter and birth weight lower than in the German speaking part. Language region combines a wide range of cultural, social and behavioural factors, including diet, smoking and alcohol consumption [27] of parents, as well as their ancestry, which probably explain its strong explanatory power. Other factors that could not be measured directly, such as health care provision, might have accounted for some of the unexplained variation. Data about the mode of delivery (vaginal or by Caesarean section, induced or spontaneous) were not available. Whilst Caesarean section rates vary geographically, they are unlikely to account for the observed spatial variation in gestational age at birth. Geographical patterns of Caesarean section are largely driven by urban-rural differences [28].

While young and old maternal age are well-known predictors of shorter gestation [29,30], the association we found with missing data on the father's nationality was somewhat unexpected. In the vast majority of cases, the information is missing because no father came forward and officially accepted paternity of the child. It is possible that missing

For peer review only - http://bmjopen.bmj.com/site/about/guidelines.xhtml

data about the father are an indicator of lower socio-economic position and social support of the mother, resulting in greater vulnerability. Studies from the United States of America found a missing name of the father on the infant's birth certificate was associated with lower education, smoking during pregnancy, preterm birth, lower birth weight, no breastfeeding and higher neonatal and post-neonatal mortality [31–34]. Children not recognised by their fathers may thus be a group at higher risk of infant and child morbidity and mothers might benefit from additional care during pregnancy and postnatally.

There are several limitations to our study. The complete case dataset was restricted to married mothers because the Swiss Live Birth Register only records birth rank if the mother was married at the time of birth. This limitation might have resulted, for example, in the weaker than expected association between birth weight and parental education. Studies from countries such as the Netherlands have shown larger gaps across levels of educational attainment, which were largest amongst unmarried women [35]. We did not have data about maternal health-related behaviours such as smoking [36], mothers' weight and height [36], disease such as gestational diabetes and data on parental genetic factors. Whilst parental nationality and education might have served as crude proxies for some missing variables, individual-level data about these factors would be valuable. A recent large-scale meta-analysis of genome-wide association data indicated that genetic factors influence birth weight through their effects on gestational age, maternal glucose metabolism, cytochrome P450 activity and possibly on maternal immune function and blood pressure [37]. Of note, compared to the foetus who carries maternal and paternal genes, maternal genes exert a larger effect on gestational age and a weaker effect on birth weight [38,39]. Examining the proportion of preterm births (before 37 weeks) or the proportion of low birth weights (<2500g) might seem clinically more relevant than the means examined in this study. However, from a statistical point of view, dichotomizing continuous data is "a practice to avoid" [40], while the mean observed in a region and the proportion of preterm and low birth weight births are highly correlated, as shown in supplementary Figure S3.

We adjusted analyses of birth weight for gestational age, which may mediate the effects of other variables, for example maternal age. Adjusting for a variable on the causal pathway has been criticised because it may introduce selection bias (or collider bias in the language of directed acyclic graphs), if there are unknown or unmeasured factors that have an effect on both gestational age and birth weight [41–43]. In our study results were broadly

For peer review only - http://bmjopen.bmj.com/site/about/guidelines.xhtml

similar with and without adjustment for gestational age and the focus of our study was not on causal inference, but on gaining an understanding of the factors contributing to spatial variation of birth weight and gestational age.

In conclusion, our study identified important differences in mean gestational age and birth weight across Switzerland. Small area variation in birth weight is largely, and in gestational age partially, explained by pregnancy-related, parental, and environmental factors.

For peer review only

For peer review only - http://bmjopen.bmj.com/site/about/guidelines.xhtml

REFERENCES

1 Regev RH, Lusky A, Dolfin T, *et al.* Excess mortality and morbidity among small-for-gestational-age premature infants: a population-based study. *J Pediatr* 2003;**143**:186–91.

2 Wilcox AJ. Birth weight and perinatal mortality: the effect of maternal smoking. *Am J Epidemiol* 1993;**137**:1098–104.

3 Barker DJ, Winter PD, Osmond C, *et al.* Weight in infancy and death from ischaemic heart disease. *Lancet* 1989;**ii**:577–80.http://www.ncbi.nlm.nih.gov/pubmed/2570282 (accessed 3 Feb 2015).

4 Lubchenco LO, Searls DT, Brazie J V. Neonatal mortality rate: relationship to birth weight and gestational age. *J Pediatr* 1972;**81**:814–22.

5 de Bernabé J V., Soriano T, Albaladejo R, *et al.* Risk factors for low birth weight: a review. *Eur J Obstet Gynecol Reprod Biol* 2004;**116**:3–15.

6 Kramer MS. Determinants of low birth weight: methodological assessment and meta-analysis. *Bull World Health Organ* 1987;**65**:663–737.

7 Secker-Walker RH, Vacek PM. Relationships between cigarette smoking during pregnancy, gestational age, maternal weight gain, and infant birthweight. *Addict Behav* 2003;**28**:55–66. doi:10.1016/S0306-4603(01)00216-7

8 Matoba N, Yu Y, Mestan K, *et al.* Differential Patterns of 27 Cord Blood Immune Biomarkers Across Gestational Age. *Pediatrics* 2009;**123**:1320–8. doi:10.1542/peds.2008-1222

9 Diez Roux A V, Mair C. Neighborhoods and health. *Ann N Y Acad Sci* 2010;**1186**:125–45. doi:10.1111/j.1749-6632.2009.05333.x

10 Macintyre S, Ellaway A, Cummins S. Place effects on health: how can we conceptualise, operationalise and measure them? *Soc Sci Med* 2002;**55**:125–39.http://www.ncbi.nlm.nih.gov/pubmed/12137182

11 Clemens T, Dibben C. Living in stressful neighbourhoods during pregnancy: An observational study of crime rates and birth outcomes. *Eur J Public Health* 2017;**27**:197–202. doi:10.1093/eurpub/ckw131

12 Kirby RS, Liu J, Lawson AB, *et al.* Spatio-temporal patterning of small area low birth weight incidence and its correlates: A latent spatial structure approach. *Spat Spatiotemporal Epidemiol* 2011;**2**:265–71. doi:10.1016/j.sste.2011.07.011

For peer review only - http://bmjopen.bmj.com/site/about/guidelines.xhtml

13 Insaf TZ, Talbot T. Identifying areas at risk of low birth weight using spatial epidemiology: A small area surveillance study. *Prev Med (Baltim)* 2016;**88**:108–14. doi:10.1016/j.ypmed.2016.03.019

14 Dibben C, Clemens T. Place of work and residential exposure to ambient air pollution and birth outcomes in Scotland, using geographically fine pollution climate mapping estimates. *Environ Res* 2015;**140**:535–41. doi:10.1016/j.envres.2015.05.010

15 Ghosh JKC, Wilhelm M, Su J, *et al.* Assessing the Influence of Traffic-related Air Pollution on Risk of Term Low Birth Weight on the Basis of Land-Use-based Regression Models and Measures of Air Toxics. *Am J Epidemiol* 2012;**175**:1262–74. doi:10.1093/aje/kwr469

16 Aebi-Popp K, Lapaire O, Glass TR, *et al.* Pregnancy and delivery outcomes of HIV infected women in Switzerland 2003-2008. *J Perinat Med* 2010;**38**:353–8. doi:10.1515/JPM.2010.052

17 Jaeger FN, Hossain M, Kiss L, *et al.* The health of migrant children in Switzerland. *Int J Public Health* 2012;**57**:659–71. doi:10.1007/s00038-012-0375-8

18 Federal Statistical Office. Births. https://www.bfs.admin.ch/bfs/de/home/statistiken/bevoelkerung/geburten-todesfaelle/geburten.html (accessed 18 Sep 2018).

19 Spörri A, Zwahlen M, Egger M, *et al.* The Swiss National Cohort: a unique database for national and international researchers. *Int J Public Heal* 2010;**55**:239–42. doi:10.1007/s00038-010-0160-5

20 Bopp M, Spörri A, Zwahlen M, *et al.* Cohort Profile: the Swiss National Cohort--a longitudinal study of 6.8 million people. *Int J Epidemiol* 2009;**38**:379–84. doi:10.1093/ije/dyn042

21 Federal Statistical Office. Structural Survey. https://www.bfs.admin.ch/bfs/en/home/statistics/population/surveys/se.html (accessed 18 Sep 2018).

22 Federal Statistical Office. Swiss MedStat regions. https://www.bfs.admin.ch/bfs/de/home/statistiken/gesundheit/nomenklaturen/medsreg.html (accessed 18 Sep 2018).

23 Moran PAP. Notes on Continuous Stochastic Phenomena. *Biometrika* 1950;**37**:17–23.http://www.jstor.org/stable/2332142

For peer review only - http://bmjopen.bmj.com/site/about/guidelines.xhtml

24 Besag J, York J, Mollié A. Bayesian image restoration, with two applications in spatial statistics. *Ann Inst Stat Math* 1991;:1–20.

25 Rue H, Martino S, Chopin N. Approximate Bayesian inference for latent Gaussian models by using integrated nested Laplace approximations. *J R Stat Soc Ser B* 2009;**71**:319–92.

26 The R Core Team: R. A Language and Environment for Statistical Computing. Vienna, Austria. 2014;**ISBN 3-900**.http://www.r-project.org.

27 Faeh D, Minder C, Gutzwiller F, *et al.* Culture, risk factors and mortality: can Switzerland add missing pieces to the European puzzle? *J Epidemiol Community Heal* 2009;**63**:639–645.

28 Federal Office of Statistics. Rate of Caeserian section (MS regions). https://www.bfs.admin.ch/bfs/de/home/statistiken/kataloge-datenbanken/karten.assetdetail.4262550.html (accessed 31 Aug 2018).

29 Kozuki N, Lee AC, Silveira MF, *et al.* The associations of parity and maternal age with small-for-gestational-age, preterm, and neonatal and infant mortality: A meta-analysis. BMC Public Health. 2013;**13**:S2. doi:10.1186/1471-2458-13-S3-S2

30 Restrepo-Méndez MC, Lawlor DA, Horta BL, *et al.* The association of maternal age with birthweight and gestational age: A cross-cohort comparison. *Paediatr Perinat Epidemiol* 2015;**29**:31–40. doi:10.1111/ppe.12162

31 Alio AP, Mbah AK, Kornosky JL, *et al.* Assessing the impact of paternal involvement on racial/ethnic disparities in infant mortality rates. *J Community Health* 2011;**36**:63–8. doi:10.1007/s10900-010-9280-3

32 Gaudino JA, Jenkins B, Rochat RW. No fathers' names: A risk factor for infant mortality in the State of Georgia, Usa. *Soc Sci Med* 1999;**48**:253–65. doi:10.1016/S0277-9536(98)00342-6

33 Cheng ER, Hawkins SS, Rifas-Shiman SL, *et al.* Association of missing paternal demographics on infant birth certificates with perinatal risk factors for childhood obesity. *BMC Public Health* 2016;**16**:1–10. doi:10.1186/s12889-016-3110-1

34 Tan H, Wen SW, Walker M, *et al.* Missing paternal demographics: A novel indicator for identifying high risk population of adverse pregnancy outcomes. *BMC Pregnancy Childbirth* 2004;**4**. doi:10.1186/1471-2393-4-21

35 Jansen PW, Tiemeier H, Looman CWN, *et al.* Explaining educational inequalities in

For peer review only - http://bmjopen.bmj.com/site/about/guidelines.xhtml

birthweight: The Generation R Study. *Paediatr Perinat Epidemiol* 2009;**23**:216–28. doi:10.1111/j.1365-3016.2009.01023.x

36 Wen SW, Goldenberg RL, Cutter GR, *et al.* Smoking, maternal age, fetal growth, and gestational age at delivery. *Am J Obstet Gynecol* 1990;**162**:53–8. doi:10.1016/0002-9378(90)90819-S

37 Beaumont RN, Warrington NM, Cavadino A, *et al.* Genome-wide association study of offspring birth weight in 86 577 women identifies five novel loci and highlights maternal genetic effects that are independent of fetal genetics. *Hum Mol Genet* 2018;**27**:742–56. doi:10.1093/hmg/ddx429

38 Beaty TH. Invited commentary: Two studies of genetic control of birth weight where large data sets were available. *Am J Epidemiol* 2007;**165**:753–5. doi:10.1093/aje/kwk106

39 Lunde A, Melve KK, Gjessing HK, *et al.* Genetic and environmental influences on birth weight, birth length, head circumference, and gestational age by use of population-based parent-offspring data. *Am J Epidemiol* 2007;**165**:734–41. doi:10.1093/aje/kwk107

40 Dawson N V., Weiss R. Dichotomizing continuous variables in statistical analysis: A practice to avoid. *Med Decis Mak* 2012;**32**:225–6. doi:10.1177/0272989X12437605

41 Cole SR, Platt RW, Schisterman EF, *et al.* Illustrating bias due to conditioning on a collider. *Int J Epidemiol* 2010;**39**:417–20. doi:10.1093/ije/dyp334

42 Delbaere I, Vansteelandt S, De Bacquer D, *et al.* Should we adjust for gestational age when analysing birth weights? The use of z-scores revisited. *Hum Reprod* 2007;**22**:2080–3. doi:10.1093/humrep/dem151

43 Wilcox AJ, Weinberg CR, Basso O. On the pitfalls of adjusting for gestational age at birth. *Am J Epidemiol* 2011;**174**:1062–8. doi:10.1093/aje/kwr230

For peer review only

For peer review only - http://bmjopen.bmj.com/site/about/guidelines.xhtml

Table 1. Characteristics of complete case and eligible study populations.

	Eligible population			Complete case population		
	No. (%)	Gestational age (days) Mean (SD)	Birth weight (g) Mean (SD)	No. (%)	Gestational age (days) Mean (SD)	Birth weight (g) Mean (SD)
**Total**	315177 (100%)	276 (12)	3328 (515)	69463 (100%)	276 (12)	3349 (501)
**Birth weight (g)**						
<1500	2141 (0.7%)	196 (27)	966 (354)	347 (0.5%)	197 (29)	969 (516)
1500-1999	2413 (0.8%)	238 (15)	1800 (142)	513 (0.7%)	239 (15)	1804 (503)
2000-2499	10036 (3.2%)	258 (14)	2312 (134)	1993 (2.9%)	258 (13)	2313 (490)
≥ 2500	300586 (95.4%)	277 (9)	3391 (423)	66609 (95.9%)	277 (9)	3404 (502)
**Gestation. age (weeks)**						
$<32^0$	2333 (0.7%)	195 (23)	1108 (527)	381 (0.5%)	195 (24)	1127 (512)
32^0-34^6	3950 (1.3%)	237 (6)	2144 (424)	800 (1.2%)	237 (6)	2138 (492)
35^0-36^6	10907 (3.5%)	253 (4)	2686 (431)	2296 (3.3%)	253 (4)	2698 (490)
$\geq 37^0$	297987 (94.5%)	278 (8)	3385 (440)	65986 (95%)	278 (8)	3399 (502)
**Sex**						
Female	152757 (48.5%)	276 (12)	3260 (494)	33698 (48.5%)	276 (11)	3277 (499)
Male	162420 (51.5%)	275 (13)	3392 (525)	35765 (51.5%)	276 (12)	3416 (504)
**Birth rank**						
1	115871 (36.8%)	276 (13)	3278 (511)	30647 (44.1%)	276 (12)	3278 (503)
2	97705 (31%)	275 (11)	3390 (493)	28544 (41.1%)	276 (11)	3389 (502)
3	28738 (9.1%)	275 (11)	3433 (502)	8154 (11.7%)	276 (11)	3438 (501)
≥ 4	7616 (2.4%)	276 (12)	3463 (527)	2118 (3%)	276 (11)	3479 (494)
missing (not married)	65247 (20.7%)	276 (14)	3263 (536)	-	-	-
**Civil status**						
Married	250055 (79.3%)	276 (12)	3345 (508)	69463 (100%)	276 (12)	3349 (502)
Single	56462 (17.9%)	276 (14)	3263 (533)	0 (0%)	-	-
Divorced	8353 (2.7%)	274 (14)	3258 (556)	0 (0%)	-	-
Widow	307 (0.1%)	274 (14)	3286 (550)	0 (0%)	-	-
**Maternal age (years)**						
mean (SD)	31.7 (5.0)			32.2 (4.6)		
< 20	2679 (0.8%)	275 (16)	3224 (554)	0 (0%)	-	-
≥ 20-25	28615 (9.1%)	277 (12)	3317 (511)	4365 (6.3%)	277 (12)	3359 (497)
≥ 25-30	82620 (26.2%)	276 (12)	3330 (506)	17764 (25.6%)	276 (11)	3346 (505)
≥ 30-35	118303 (37.5%)	276 (12)	3335 (510)	28108 (40.5%)	276 (12)	3351 (501)
≥ 35-40	67914 (21.5%)	275 (12)	3333 (523)	16021 (23.1%)	275 (11)	3353 (501)
≥ 40	15046 (4.8%)	273 (14)	3286 (555)	3205 (4.6%)	273 (14)	3304 (501)
**Nationality mother**						
Switzerland	194570 (61.7%)	276 (12)	3322 (511)	46651 (67.2%)	276 (11)	3342 (504)
Southern Europe	23585 (7.5%)	275 (12)	3251 (494)	4763 (6.9%)	276 (11)	3269 (501)
Western Europe	26005 (8.3%)	276 (12)	3348 (516)	4799 (6.9%)	276 (12)	3369 (499)
Northern Europe	3695 (1.2%)	276 (13)	3418 (510)	703 (1%)	276 (13)	3433 (489)
Eastern Europe	38762 (12.3%)	276 (13)	3397 (523)	7743 (11.1%)	276 (12)	3428 (499)
Other	28560 (9.1%)	275 (14)	3313 (535)	4804 (6.9%)	275 (13)	3331 (492)
**Nationality father**						
Switzerland	191589 (60.8%)	276 (12)	3329 (506)	47018 (67.7%)	276 (12)	3346 (504)
Southern Europe	31466 (10%)	275 (12)	3256 (493)	6473 (9.3%)	275 (11)	3273 (499)
Western Europe	26954 (8.6%)	276 (12)	3353 (518)	4949 (7.1%)	276 (12)	3376 (486)
Northern Europe	3911 (1.2%)	276 (12)	3406 (510)	724 (1%)	276 (13)	3412 (492)
Eastern Europe	35387 (11.2%)	276 (13)	3397 (528)	6960 (10%)	276 (12)	3424 (503)
Other	21077 (6.7%)	276 (13)	3307 (531)	3339 (4.8%)	276 (12)	3318 (502)
missing	4793 (1.5%)	272 (23)	3148 (693)	-	-	-
**Education mother**						
Tertiary	42088 (13.4%)	276 (12)	3344 (500)	28016 (40.3%)	276 (12)	3356 (499)
Secondary	48878 (15.5%)	276 (12)	3328 (509)	32614 (47.0%)	276 (12)	3343 (505)
Compulsory	14642 (4.6%)	275 (13)	3329 (534)	8833 (12.7%)	275 (12)	3345 (501)
Unknown (age <20 yrs)	2679 (0.8%)	275 (16)	3224 (554)	-	-	-
missing	206890 (65.6%)	276 (12)	3326 (517)	-	-	-
**Education father**						
Tertiary	49848 (15.8%)	276 (12)	3348 (497)	34325 (49.4%)	276 (12)	3357 (501)
Secondary	41301 (13.1%)	276 (12)	3323 (511)	26857 (38.7%)	276 (12)	3340 (502)
Compulsory	13731 (4.4%)	276 (12)	3323 (514)	8281 (11.9%)	275 (12)	3340 (506)
missing	210297 (66.7%)	276 (13)	3325 (519)	-	-	-
**Altitude (m)**						
mean (SD)	515 (189)			511 (181)		
**Urbanisation**						
Urban	96643 (30.7%)	276 (13)	3326 (517)	18516 (26.7%)	276 (12)	3344 (498)
Peri-urban	138826 (44%)	275 (12)	3329 (514)	31430 (45.2%)	276 (12)	3348 (501)
Rural	79708 (25.3%)	276 (12)	3329 (512)	19517 (28.1%)	276 (12)	3354 (506)
**Language region**						
German	223586 (70.9%)	276 (12)	3348 (515)	46546 (67%)	276 (12)	3370 (502)
French	80068 (25.4%)	275 (12)	3283 (512)	19324 (27.8%)	275 (11)	3310 (502)
Italian	11523 (3.7%)	275 (12)	3252 (494)	3593 (5.2%)	275 (12)	3273 (494)

For peer review only - http://bmjopen.bmj.com/site/about/guidelines.xhtml

Table 2. Associations of mean gestational age at birth and mean birth weight with pregnancy, parental and environmental factors from adjusted linear mixed-effects model (model 3).

	Gestational age (days) Absolute differences (95% CI)		Birth weight (g) * Relative differences (95% CI)	
	Eligible population	Complete case population	Eligible population	Complete case population
Intercept	277.3 (277.2 to 277.5)	278.0 (277.7 to 278.3)	3276 (3215 to 3337)&	3294 (3167 to 3426) &
**Sex**				
Female	0	0	1	1
Male	-0.56 (-0.65 to -0.48)	-0.61 (-0.79 to -0.44)	1.045 (1.044 to 1.046)	1.048 (1.046 to 1.050)
**Birth rank**				
1¶	0	0	1	1
2	-0.44 (-0.54 to -0.33)	-0.42 (-0.61 to -0.23)	1.037 (1.036 to 1.038)	1.038 (1.036 to 1.040)
3	-0.28 (-0.44 to -0.11)	-0.18 (-0.47 to 0.11)	1.050 (1.048 to 1.051)	1.053 (1.050 to 1.056)
≥ 4	0.15 (-0.14 to 0.43)	0.56 (0.03 to 1.08)	1.058 (1.055 to 1.061)	1.064 (1.059 to 1.070)
missing (not married)	-0.11 (-0.24 to 0.01)	-	1.003 (1.002 to 1.004)	-
**Maternal age (yrs)**				
< 20	-3.92 (-5.41 to -2.44)	-	0.980 (0.965 to 0.994)	-
[20-30)¶	0	0	1	1
[30-40)	-1.01 (-1.09 to -0.94)	-1.04 (-1.19 to -0.88)	1.002 (1.001 to 1.003)	0.999 (0.998 to 1.001)
≥ 40	-2.94 (-3.36 to -2.51)	-3.61 (-4.51 to -2.71)	1.000 (0.996 to 1.004)	1.003 (0.993 to 1.012)
**Nationality mother**				
Switzerland¶	0	0	1	1
S Europe	0.21 (0.01 to 0.41)	0.37 (-0.06 to 0.80)	0.994 (0.992 to 0.996)	0.995 (0.991 to 1.000)
W Europe	0.21 (0.03 to 0.39)	0.04 (-0.35 to 0.42)	1.007 (1.005 to 1.009)	1.006 (1.002 to 1.009)
N Europe	0.38 (-0.06 to 0.82)	0.09 (-0.85 to 1.04)	1.024 (1.019 to 1.028)	1.026 (1.016 to 1.036)
E Europe	0.21 (0.04 to 0.38)	0.35 (0.00 to 0.71)	1.013 (1.011 to 1.014)	1.017 (1.014 to 1.021)
Other	-0.32 (-0.50 to -0.15)	-0.58 (-0.96 to -0.19)	1.007 (1.006 to 1.009)	1.010 (1.006 to 1.014)
**Nationality father**				
Switzerland¶	0	0	1	1
S Europe	-0.46 (-0.64 to -0.27)	-0.15 (-0.52 to 0.23)	0.992 (0.990 to 0.993)	0.992 (0.988 to 0.996)
W Europe	0.08 (-0.10 to 0.25)	0.07 (-0.31 to 0.45)	1.007 (1.006 to 1.009)	1.006 (1.002 to 1.010)
N Europe	0.52 (0.09 to 0.95)	-0.10 (-1.04 to 0.83)	1.012 (1.008 to 1.017)	1.011 (1.001 to 1.020)
E Europe	-0.47 (-0.65 to -0.29)	0.04 (-0.33 to 0.42)	1.009 (1.008 to 1.011)	1.012 (1.008 to 1.016)
Other	-0.03 (-0.23 to 0.17)	0.42 (-0.02 to 0.87)	0.994 (0.992 to 0.996)	0.991 (0.986 to 0.995)
missing	-3.88 (-4.25 to -3.52)	-	0.989 (0.985 to 0.993)	-
**Education mother**				
Tertiary¶		0		1
Secondary		-0.56 (-0.76 to -0.35)		0.997 (0.995 to 0.999)
Compulsory		-0.90 (-1.24 to -0.56)		0.993 (0.990 to 0.997)
**Education father**				
Tertiary¶		0		1
Secondary		-0.14 (-0.35 to 0.06)		0.997 (0.995 to 0.999)
Compulsory		-0.38 (-0.73 to -0.04)		0.997 (0.994 to 1.001)
**Altitude (m)**				
500¶	0	0	1	1
per 500 m increase	0.07 (-0.09 to 0.22)	0.04 (-0.24 to 0.32)	0.989 (0.988 to 0.991)	0.988 (0.985 to 0.991)
**Urbanisation**				
Urban¶	0	0	1	1
Peri-urban	-0.43 (-0.57 to -0.28)	-0.51 (-0.75 to -0.27)	1.001 (1.000 to 1.003)	1.002 (1.000 to 1.005)
Rural	-0.16 (-0.33 to 0.00)	-0.18 (-0.45 to 0.10)	1.001 (0.999 to 1.002)	1.003 (1.001 to 1.006)
**Language region**				
German¶	0	0	1	1
French	-0.62 (-0.77 to -0.47)	-0.66 (-0.88 to -0.43)	0.989 (0.988 to 0.99)	0.989 (0.987 to 0.991)
Italian	-0.94 (-1.26 to -0.63)	-1.29 (-1.75 to -0.84)	0.982 (0.98 to 0.985)	0.983 (0.979 to 0.988)
**Percent of spatial variance explained†**	31%	41%	87%	82%

*Birth weight was modeled on a log scale, which results in multiplicative effects. The model for birth weight was additionally adjusted for gestational age by a cubic spline function with knots at weeks 25, 30 and 35.

& In the model for BW, the intercept corresponds to an estimated mean birth weight (g) for a singleton girl born at gestational age 40 weeks as the first child (rank 1) in a German-speaking, urban region of elevation 500m, whose mother is 20-30 years old at birth and married, and both parents have Swiss nationality and tertiary education.

¶ Reference category

† Percentage of regional variance explained by model predictors, i.e. percent reduction in variance of random effects (σ^2) when compared to model with no predictors (model 0).

For peer review only - http://bmjopen.bmj.com/site/about/guidelines.xhtml

Table 3. Percentage of spatial variation explained by each individual variable and explained in addition after adjusting for all other variables.

		Gestational age		Birth weight	
**Spatial variation explained**		**Eligible population**	**Complete case population**	**Eligible population**	**Complete case population**
**By single variables**					
**Pregnancy factors**	Gestational age	-	-	27%	31%
	Sex	0%	0%	1%	3%
	Birth rank	-	0%	-	3%
	Birth rank missing (not married)	0%	-	12%	-
**Parental factors**	Maternal age	0%	4%	1%	1%
	Nationality mother	1%	3%	17%	19%
	Nationality father	-	4%	-	21%
	Missing data on father	3%	-	25%	-
	Nationality parents*	3%	5%	27%	24%
	Education mother	-	0%	-	0%
	Education father	-	1%	-	1%
	Education parents*	-	0%	-	1%
**Regional factors**	Altitude	0%	0%	10%	6%
	Urbanisation	10%	9%	0%	0%
	Language region	23%	33%	62%	60%
**In addition to all other variables**					
**Pregnancy factors**	Gestational age	-	-	11%	5%
	Sex	0%	0%	0%	1%
	Birth rank	-	0%	-	3%
	Birth rank missing (not married)	0%	-	4%	-
**Parental factors**	Maternal age	0%	1.5%	1%	0%
	Nationality mother	0%	0%	0.5%	2%
	Nationality father	-	0%	-	0%
	Missing data on father	1.5%	-	0.5%	-
	Nationality parents*	2.5%	0%	2.5%	4%
	Education mother	-	1.5%	-	0%
	Education father	-	0%	-	0%
	Education parents*	-	1.5%	-	0.5%
**Regional Factors**	Altitude	0%	0%	9%	6%
	Urbanisation	9%	7%	0%	1%
	Language region	19%	26%	21%	21%
	Model 3 (full)	31%	42%	87%	82%

-, data not available; *, nationality or educational attainment of mother and father were entered into the model.

For peer review only - http://bmjopen.bmj.com/site/about/guidelines.xhtml

FIGURE LEGENDS

Figure 1. Maps of average gestational age (upper two panels) and birth weight (lower two panels) observed across 705 Swiss areas. Left: all eligible live births (n=315,177), right: complete case population (n=69,463).

Figure 2. Maps of gestational age and birth weight from crude model (model 0) and multivariable linear mixed-effect models (models 1-3) with percent reduction in the regional variation, represented by random effects. Analyses based on complete case population (*N* = 69,463).

FOOTNOTES

Contributors: ME and CEK conceived the study and obtained funding. VS, ME, MZ and CEK developed the analysis plan. VS did all statistical analyses and wrote the first draft of the paper, which was revised by ME taking into account the critical comments from all authors. ME supervised the study. All authors approved the final version of the report.

Funding: The Swiss National Cohort is funded by the Swiss National Science Foundation (SNSF) cohort grant No. 148415. The current analysis was funded by SNSF project grant No. 163452. ME was supported by special SNSF project funding (grant No. 174281).

Competing interests: None declared.

Patient consent: Not required.

Ethics approval: The SNC has been approved by the Ethics Committee of the Canton of Bern.

Provenance and peer review: Not commissioned; externally peer reviewed.

Data sharing statement: Data are available within the framework of a data sharing agreement.

For peer review only - http://bmjopen.bmj.com/site/about/guidelines.xhtml

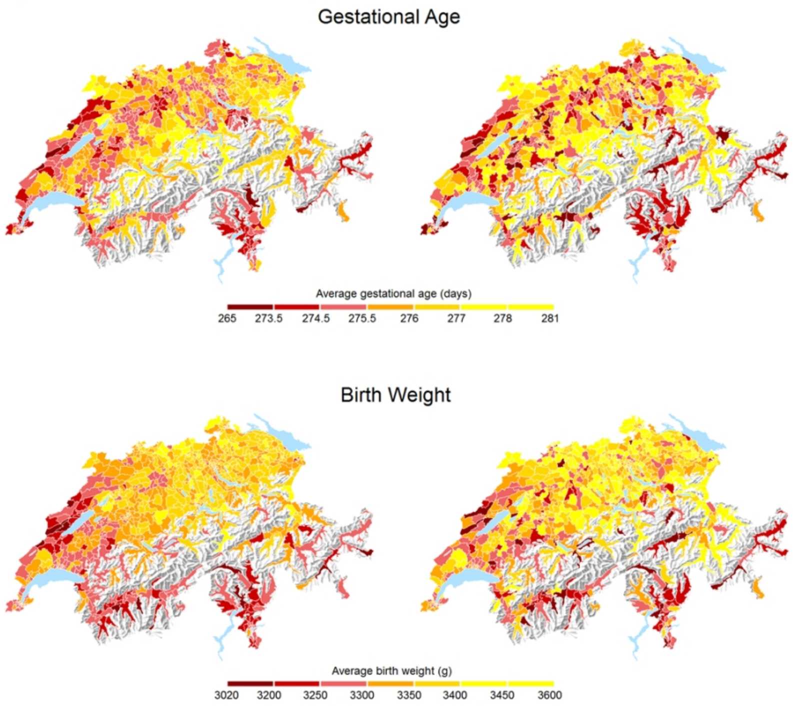

Figure 1. Maps of average gestational age (upper two panels) and birth weight (lower two panels) observed across 705 Swiss areas. Left: all eligible live births (n=315,177), right: complete case population (n=69,463).

338x254mm (96 x 96 DPI)

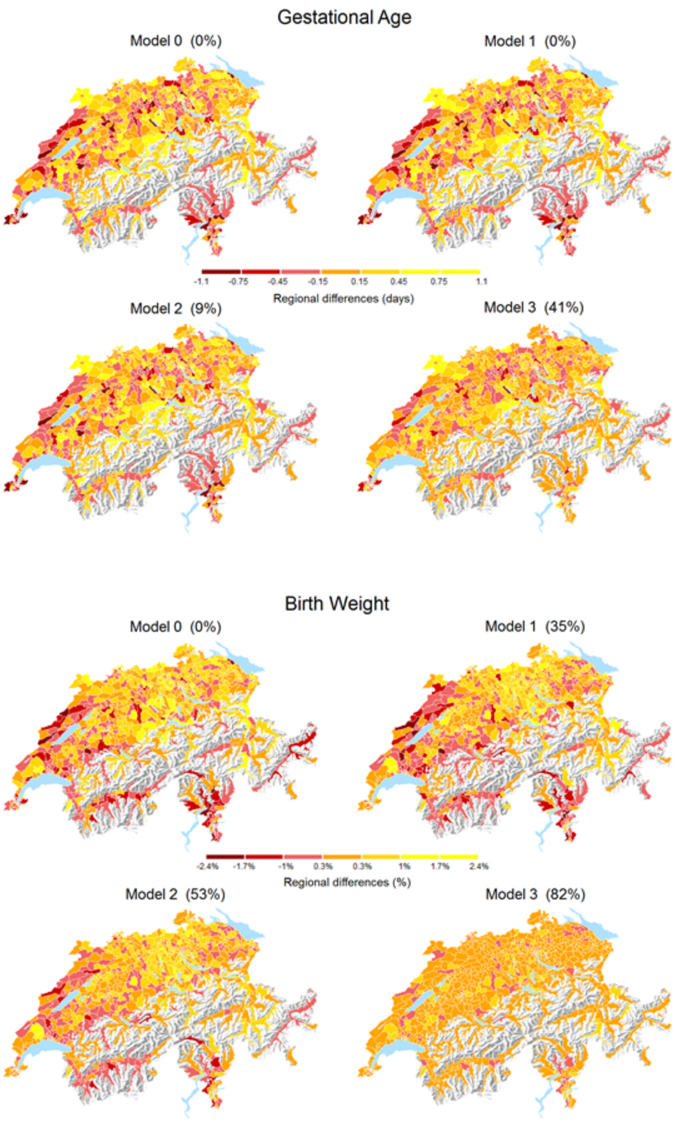

Figure 2. Maps of gestational age and birth weight from crude model (model 0) and multivariable linear mixed-effect models (models 1-3) with percent reduction in the regional variation, represented by random effects. Analyses based on complete case population (N = 69,463).

190x254mm (96 x 96 DPI)

Supplementary Table S1. Number of live births, mean gestational age and mean birth weight by maternal nationality in the eligible population (*N* = 315'177).

Nationality	N	Gestational age (days) Mean (SD)	Birth weight (g) Mean (SD)
**Switzerland**	194,570	276 (12)	3322 (511)
**Southern Europe**			
**Andorra**	1	279 (-)	3080 (-)
**Italy**	8337	275 (12)	3271 (496)
**Malta**	13	273 (8)	3188 (427)
**Portugal**	12,368	276 (12)	3235 (493)
**San Marino**	2	274 (1.4)	3485 (120)
**Spain**	2864	276 (12)	3263 (488)
**Western Europe**			
**Austria**	1555	275 (14)	3328 (528)
**Belgium**	583	276 (12)	3357 (482)
**Germany**	16,736	276 (13)	3369 (517)
**France**	6173	276 (12)	3294 (505)
**Lichtenstein**	100	275 (11)	3369 (488)
**Luxembourg**	55	276 (19)	3396 (636)
**Netherlands**	803	276 (12)	3377 (529)
**Northern Europe**			
**Denmark**	271	276 (13)	3383 (511)
**Estonia**	81	279 (7)	3601 (466)
**Finland**	312	276 (11)	3465 (523)
**Ireland**	212	276 (16)	3446 (548)
**Iceland**	31	272 (25)	3180 (775)
**Latvia**	187	279 (9)	3493 (434)
**Lithuania**	152	277 (13)	3450 (535)
**Norway**	110	275 (11)	3390 (525)
**Sweden**	571	276 (12)	3422 (470)
**UK**	1768	276 (13)	3397 (513)
**Eastern Europe**			
**Czech Republic**	623	275 (12)	3339 (499)
**Hungary**	913	275 (13)	3341 (512)
**Poland**	1778	276 (12)	3399 (497)
**Slovakia**	1068	276 (12)	3348 (509)
**Albania**	209	276 (12)	3406 (476)
**Bosnia & Herzegovina**	1952	276 (12)	3466 (492)
**Croatia**	1582	276 (12)	3448 (540)
**Kosovo**	10,278	276 (13)	3421 (530)
**Macedonia**	5842	276 (13)	3392 (514)
**Montenegro**	212	276 (10)	3416 (466)
**Serbia**	5195	276 (13)	3400 (536)
**Serbia & Montenegro**	10	277 (8)	3637 (250)
**Slovenia**	163	275 (15)	3366 (589)
**Cyprus**	15	278 (8)	3411 (525)
**Bulgaria**	406	273 (15)	3291 (559)
**Greece**	375	274 (13)	3317 (516)
**Romania**	971	274 (14)	3284 (537)
**Turkey**	4441	275 (13)	3347 (523)
**Belarus**	172	277 (13)	3385 (508)
**Moldova**	135	276 (10)	3496 (515)
**Russia**	1567	277 (12)	3427 (513)
**Ukraine**	855	277 (11)	3412 (473)
**Other (non-Europe)**			
6 most numerous: **Eritrea**	2600	279 (14)	3380 (528)
**Brazil**	2381	274 (12)	3312 (498)
**Sri Lanka**	1391	273 (14)	3158 (553)
**USA**	1291	276 (14)	3378 (532)
**China**	1293	276 (13)	3425 (541)
**Morocco**	1159	276 (14)	3378 (536)
. . .			
**Total**	315,177	276 (12)	3328 (515)

For peer review only - http://bmjopen.bmj.com/site/about/guidelines.xhtml

Supplementary Table S2. Comparison of results from fully adjusted model (model 3) accounting and not accounting for spatial autocorrelation. Based on complete-case population (*N* = 69'463).

	Accounting for spatial autocorrelation		Not accounting for spatial autocorrelation	
	Gestational age (days) Absolute differences (95% CI)	Birth weight (g) * Relative differences (95% CI)	Gestational age (days) Absolute differences (95% CI)	Birth weight (g) * Relative differences (95% CI)
Intercept	277.9 (277.6, 278.2)	3289 (3159, 3423)	278.0 (277.7 to 278.3)	3294 (3167 to 3426) [&]
**Sex**				
Female	0	1	0	1
Male	-0.61 (-0.79, -0.44)	1.048 (1.046, 1.049)	-0.61 (-0.79 to -0.44)	1.048 (1.046 to 1.050)
**Birth rank**				
1[¶]	0	1	0	1
2	-0.42 (-0.61, -0.23)	1.038 (1.036, 1.040)	-0.42 (-0.61 to -0.23)	1.038 (1.036 to 1.040)
3	-0.18 (-0.47, 0.11)	1.053 (1.050, 1.056)	-0.18 (-0.47 to 0.11)	1.053 (1.050 to 1.056)
≥ 4	0.55 (0.03, 1.08)	1.064 (1.059, 1.070)	0.56 (0.03 to 1.08)	1.064 (1.059 to 1.070)
**Maternal age (yrs)**				
[20-30)[¶]	0	1	0	1
[30-40)	-1.03 (-1.19, -0.87)	0.999 (0.997, 1.001)	-1.04 (-1.19 to -0.88)	0.999 (0.998 to 1.001)
≥ 40	-3.61 (-4.52, -2.71)	1.003 (0.993, 1.012)	-3.61 (-4.51 to -2.71)	1.003 (0.993 to 1.012)
**Nationality mother**				
Switzerland[¶]	0	1	0	1
S Europe	0.37 (-0.07, 0.80)	0.995 (0.991, 1.000)	0.37 (-0.06 to 0.80)	0.995 (0.991 to 1.000)
W Europe	0.03 (-0.35, 0.42)	1.005 (1.001, 1.009)	0.04 (-0.35 to 0.42)	1.006 (1.002 to 1.009)
N Europe	0.10 (-0.85, 1.05)	1.025 (1.015, 1.035)	0.09 (-0.85 to 1.04)	1.026 (1.016 to 1.036)
E Europe	0.35 (0.00, 0.71)	1.017 (1.013, 1.021)	0.35 (0.00 to 0.71)	1.017 (1.014 to 1.021)
Other	-0.56 (-0.95, -0.18)	1.010 (1.006, 1.014)	-0.58 (-0.96 to -0.19)	1.010 (1.006 to 1.014)
**Nationality father**				
Switzerland[¶]	0	1	0	1
S Europe	-0.14 (-0.52, 0.23)	0.992 (0.988, 0.995)	-0.15 (-0.52 to 0.23)	0.992 (0.988 to 0.996)
W Europe	0.07 (-0.31, 0.45)	1.006 (1.002, 1.010)	0.07 (-0.31 to 0.45)	1.006 (1.002 to 1.010)
N Europe	-0.08 (-1.02, 0.85)	1.010 (1.001, 1.020)	-0.10 (-1.04 to 0.83)	1.011 (1.001 to 1.020)
E Europe	0.04 (-0.34, 0.42)	1.011 (1.007, 1.015)	0.04 (-0.33 to 0.42)	1.012 (1.008 to 1.016)
Other	0.44 (-0.01, 0.89)	0.991 (0.986, 0.995)	0.42 (-0.02 to 0.87)	0.991 (0.986 to 0.995)
**Education mother**				
Tertiary[¶]	0	1	0	1
Secondary	-0.56 (-0.77, -0.36)	0.997 (0.995, 0.999)	-0.56 (-0.76 to -0.35)	0.997 (0.995 to 0.999)
Compulsory	-0.91 (-1.25, -0.57)	0.994 (0.990, 0.997)	-0.90 (-1.24 to -0.56)	0.993 (0.990 to 0.997)
**Education father**				*
Tertiary[¶]	0	1	0	1
Secondary	-0.14 (-0.35, 0.06)	0.997 (0.995, 0.999)	-0.14 (-0.35 to 0.06)	0.997 (0.995 to 0.999)
Compulsory	-0.39 (-0.73, -0.04)	0.997 (0.994, 1.001)	-0.38 (-0.73 to -0.04)	0.997 (0.994 to 1.001)
**Altitude (m)**				
500[¶]	0	1	0	1
per 500 m increase	-0.01 (-0.31, 0.29)	0.989 (0.985, 0.993)	0.04 (-0.24 to 0.32)	0.988 (0.985 to 0.991)
**Urbanisation**				
Urban[¶]	0	1	0	1
Peri-urban	-0.49 (-0.73, -0.25)	1.001 (0.998, 1.004)	-0.51 (-0.75 to -0.27)	1.002 (1.000 to 1.005)
Rural	-0.19 (-0.47, 0.09)	1.002 (0.998, 1.006)	-0.18 (-0.45 to 0.10)	1.003 (1.001 to 1.006)
**Language region**				
German[¶]	0	1	0	1
French	-0.41 (-0.77, -0.05)	0.992 (0.984, 1.000)	-0.66 (-0.88 to -0.43)	0.989 (0.987 to 0.991)
Italian	-1.29 (-1.75, -0.83)	0.984 (0.978, 0.990)	-1.29 (-1.75 to -0.84)	0.983 (0.979 to 0.988)

*Birth weight was modeled on a log scale, which results in multiplicative effects. The model for birth weight was additionally adjusted for gestational age by a cubic spline function with knots at weeks 25, 30 and 35.
[&] In the model for BW, the intercept corresponds to an estimated mean birth weight (g) for a singleton girl born at gestational age 40 weeks as the first child (rank 1) in a German-speaking, urban region of elevation 500m, whose mother is 20-30 years old at birth and married, and both parents have Swiss nationality and tertiary education.
[¶] Reference category
† Percentage of regional variance explained by model predictors, i.e. percent reduction in variance of random effects (σ^2) when compared to model with no predictors (model 0).

For peer review only - http://bmjopen.bmj.com/site/about/guidelines.xhtml

Supplementary Table S3. Comparison of results from model (model 3) for birth weight, adjusted and not adjusted for gestational age.

	Birth weight - Model 3 without Gestational Age		Birth weight - Model 3 with Gestational Age*	
	Relative differences (95% CI)		Relative differences (95% CI)	
	Complete case population	Eligible population	Complete case population	Eligible population
**Intercept**	3209 (3195, 3222)	3186 (3179, 3193)	3294 (3167 to 3426)	3276 (3215 to 3337)
**Sex**				
¶Female	1	1	1	1
Male	1.042 (1.039, 1.044)	1.040 (1.038, 1.041)	1.048 (1.046 to 1.050)	1.045 (1.044 to 1.046)
**Rank**				
1¶	1	1	1	1
2	1.039 (1.036, 1.042)	1.038 (1.036, 1.040)	1.038 (1.036 to 1.040)	1.037 (1.036 to 1.038)
3	1.057 (1.052, 1.062)	1.052 (1.049, 1.055)	1.053 (1.050 to 1.056)	1.050 (1.048 to 1.051)
≥ 4	1.074 (1.065, 1.082)	1.063 (1.058, 1.067)	1.064 (1.059 to 1.070)	1.058 (1.055 to 1.061)
missing (non-married)	-	1.002 (1.000, 1.003)	-	1.003 (1.002 to 1.004)
**Age mother (yrs)**				
< 20 yrs (per 5 yrs)	-	0.934 (0.913, 0.955)	-	0.980 (0.965 to 0.994)
20-30 yrs¶	1	1	1	1
30-40 yrs (per 5 yrs)	0.990 (0.988, 0.993)	0.993 (0.992, 0.994)	0.999 (0.998 to 1.001)	1.002 (1.001 to 1.003)
> 40 yrs (per 5 yrs)	0.969 (0.956, 0.982)	0.974 (0.968, 0.981)	1.003 (0.993 to 1.012)	1.000 (0.996 to 1.004)
**Nationality mother**				
Switzerland¶	1	1	1	1
S Europe	0.999 (0.992, 1.005)	0.996 (0.993, 0.999)	0.995 (0.991 to 1.000)	0.994 (0.992 to 0.996)
W Europe	1.006 (1.000, 1.012)	1.009 (1.006, 1.012)	1.006 (1.002 to 1.009)	1.007 (1.005 to 1.009)
N Europe	1.024 (1.010, 1.039)	1.026 (1.019, 1.033)	1.026 (1.016 to 1.036)	1.024 (1.019 to 1.028)
E Europe	1.020 (1.015, 1.026)	1.014 (1.011, 1.016)	1.017 (1.014 to 1.021)	1.013 (1.011 to 1.014)
Other	1.004 (0.998, 1.010)	1.004 (1.001, 1.007)	1.010 (1.006 to 1.014)	1.007 (1.006 to 1.009)
**Nationality father**				
Switzerland¶	1	1	1	1
S Europe	0.992 (0.986, 0.998)	0.988 (0.986, 0.991)	0.992 (0.988 to 0.996)	0.992 (0.990 to 0.993)
W Europe	1.007 (1.001, 1.013)	1.008 (1.005, 1.010)	1.006 (1.002 to 1.010)	1.007 (1.006 to 1.009)
N Europe	1.008 (0.994, 1.022)	1.016 (1.010, 1.023)	1.011 (1.001 to 1.020)	1.012 (1.008 to 1.017)
E Europe	1.011 (1.005, 1.017)	1.004 (1.001, 1.007)	1.012 (1.008 to 1.016)	1.009 (1.008 to 1.011)
Other	0.995 (0.989, 1.002)	0.991 (0.989, 0.994)	0.991 (0.986 to 0.995)	0.994 (0.992 to 0.996)
missing	-	0.933 (0.928, 0.938)	-	0.989 (0.985 to 0.993)
**Education mother**				
Tertiary¶	1		1	
Secondary	0.993 (0.990, 0.996)		0.997 (0.995 to 0.999)	
Compulsory	0.984 (0.979, 0.989)		0.993 (0.990 to 0.997)	
**Education father**				
Tertiary¶	1		1	
Secondary	0.996 (0.993, 0.999)		0.997 (0.995 to 0.999)	
Compulsory	0.994 (0.989, 1.000)		0.997 (0.994 to 1.001)	
**Altitude (m)**				
500 m¶	1	1	1	1
per 500 m increase	0.988 (0.984, 0.992)	0.990 (0.988, 0.992)	0.988 (0.985 to 0.991)	0.989 (0.988 to 0.991)
**Urbanisation**				
Urban¶	1	1	1	1
Peri-urban	0.999 (0.996, 1.002)	0.998 (0.996, 1.000)	1.002 (1.000 to 1.005)	1.001 (1.000 to 1.003)
Rural	1.002 (0.998, 1.006)	0.999 (0.997, 1.002)	1.003 (1.001 to 1.006)	1.001 (0.999 to 1.002)
**Language region**				
RGerman¶	1	1	1	1
French	0.985 (0.982, 0.988)	0.985 (0.983, 0.987)	0.989 (0.987 to 0.991)	0.989 (0.988 to 0.99)
Italian	0.974 (0.968, 0.981)	0.977 (0.973, 0.981)	0.983 (0.979 to 0.988)	0.982 (0.98 to 0.985)
**% variation explained**				
Model 3	77%	77%	82%	87%
Model 2	28%	30%	53%	53%
Model 1	6%	12%	35%	35%

*Birth weight was modelled on a log scale, which results in multiplicative effects. The model was additionally adjusted for gestational age by a cubic spline function with knots at weeks 25, 30 and 35.

¶ Reference category

† Percentage of regional variance explained by model predictors, i.e. percent reduction in variance of random effects (σ^2) when compared to model with no predictors (model 0).

Supplementary Figure S1. Selection of eligible and complete case study populations among live births in Switzerland 2011 to 2014.

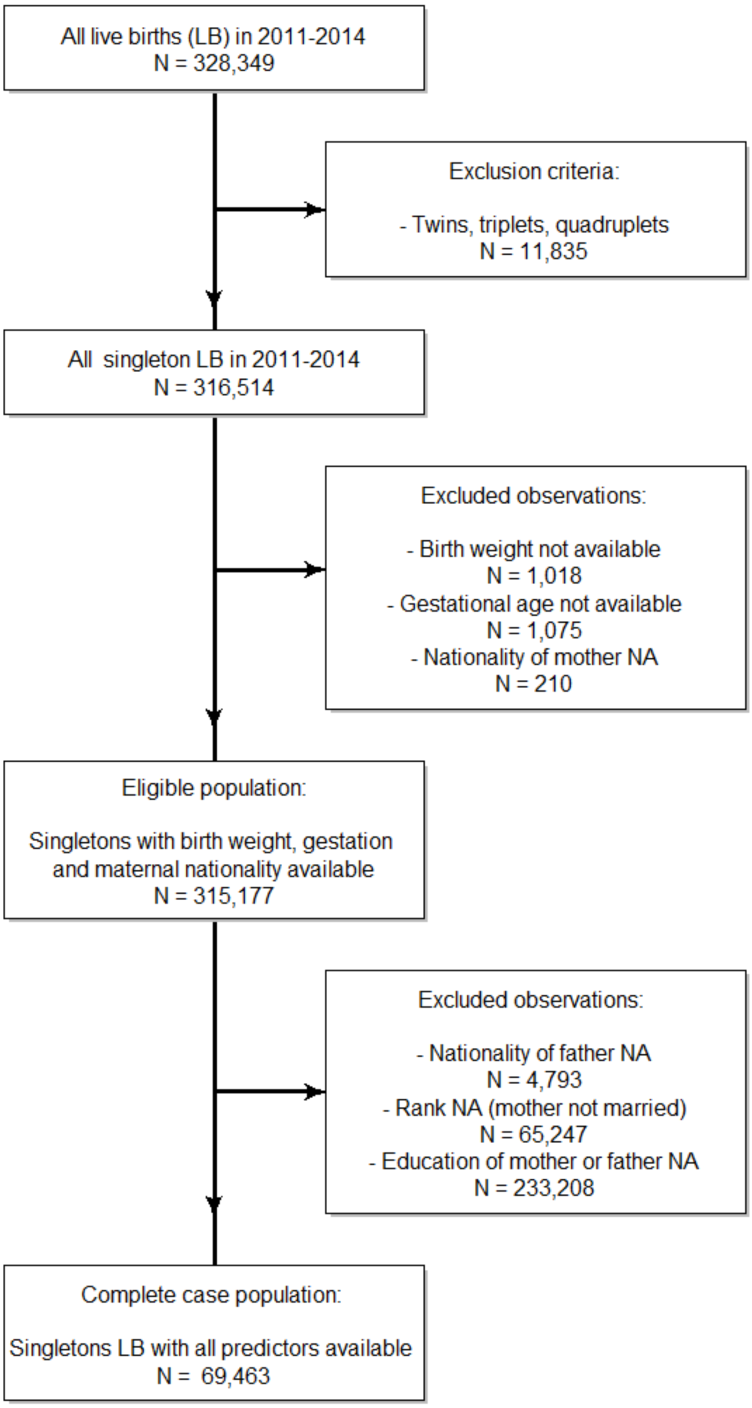

For peer review only - http://bmjopen.bmj.com/site/about/guidelines.xhtml

Supplementary Figure S2. Relationship between birth weight and gestational age at birth modeled by a cubic spline function. Separate fitted curves are shown for newborn girls and boys, with all other predictors corresponding to the reference categories shown in Table 2.

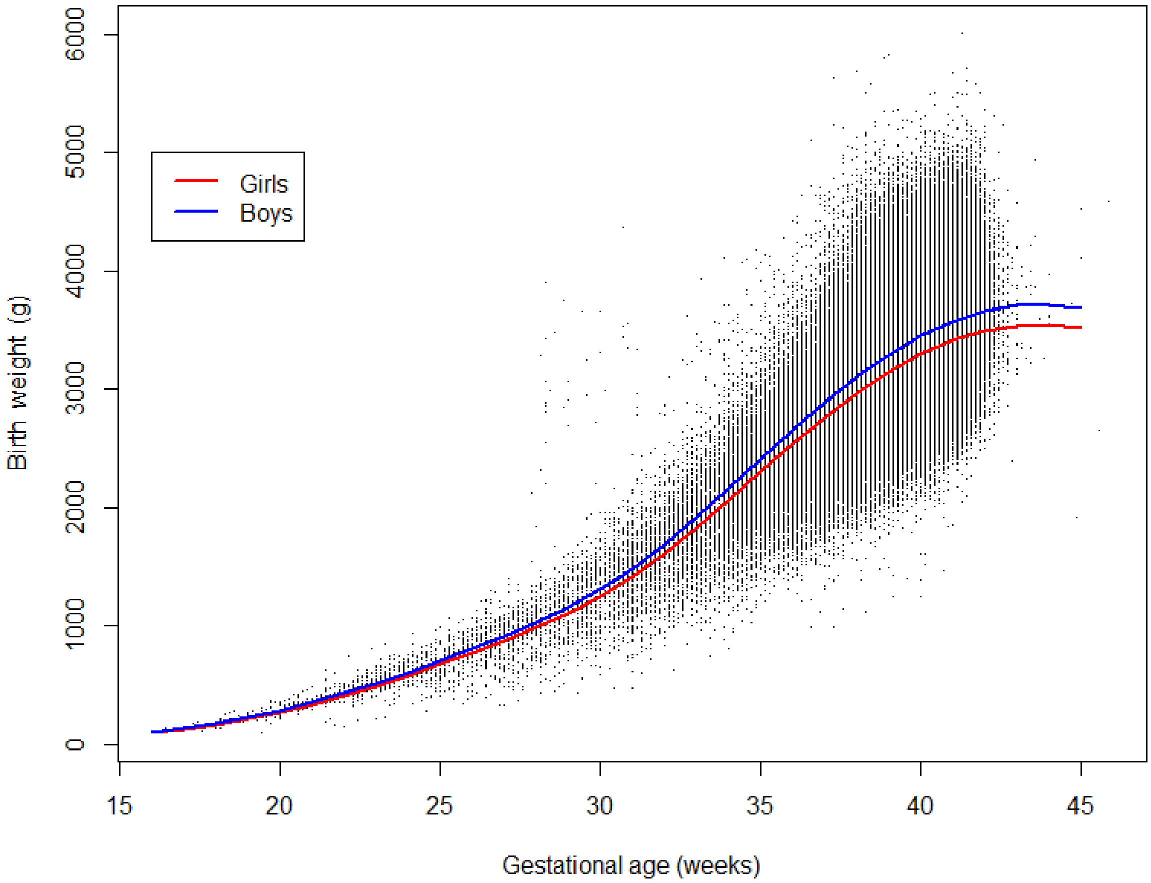

For peer review only - http://bmjopen.bmj.com/site/about/guidelines.xhtml

Supplementary Figure S3. Relationship between mean gestational age and proportion of preterm live births (<37 weeks) among eligible live births across 705 regions (upper panel) and between mean birth weight and proportion of low birth weight births (<2500g) (lower panel).
Results from linear regression weighted by the number of live births in each region. Prediction interval displayed for an average-size region (n=447). GA = gestational age; BW= birth weight

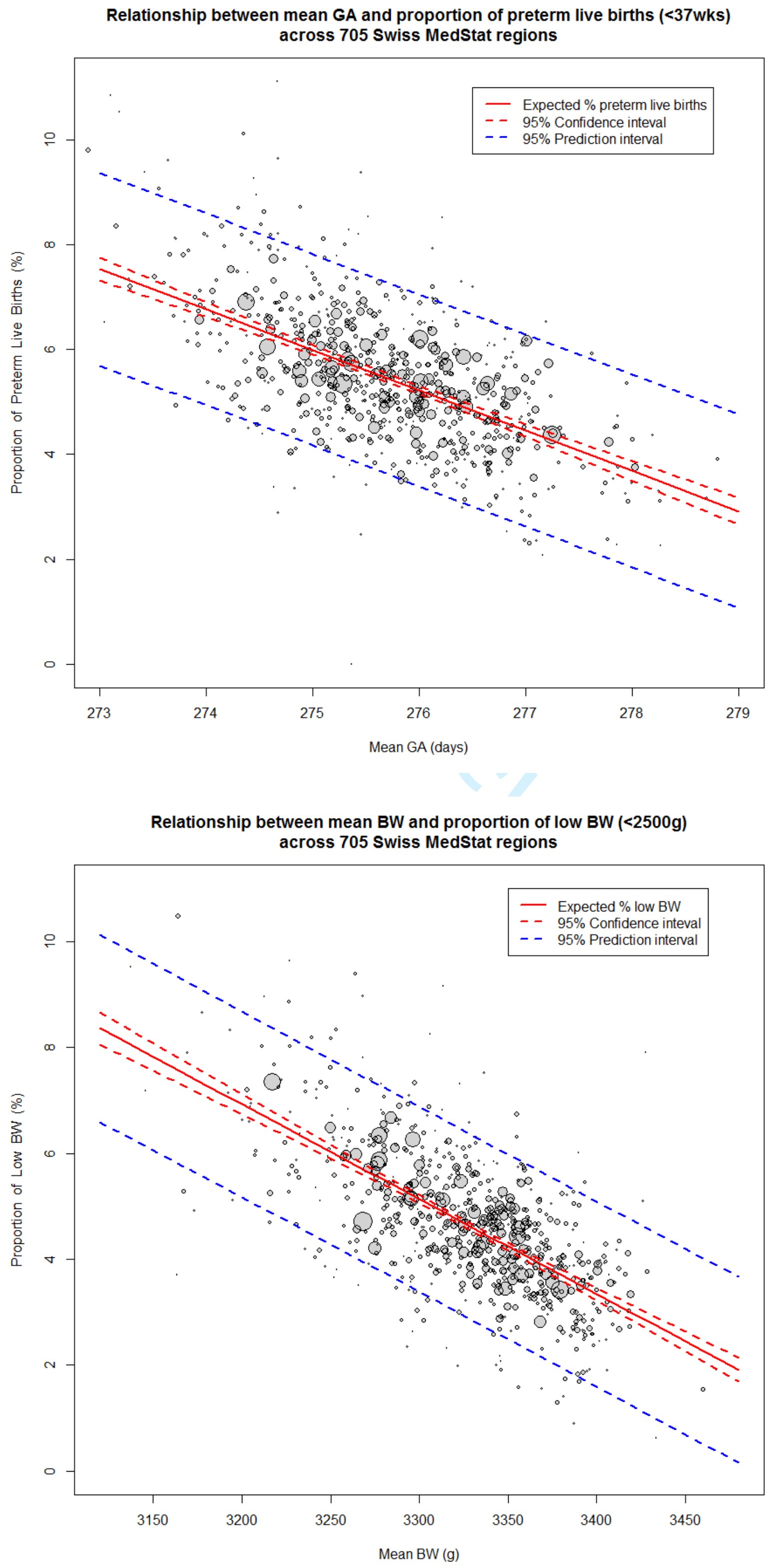

STROBE Statement—checklist of items that should be included in reports of observational studies

	Item No	Recommendation	Page No
**Title and abstract**	1	(*a*) Indicate the study's design with a commonly used term in the title or the abstract	1
		(*b*) Provide in the abstract an informative and balanced summary of what was done and what was found	2
**Introduction**			
Background/rationale	2	Explain the scientific background and rationale for the investigation being reported	4
Objectives	3	State specific objectives, including any prespecified hypotheses	4
**Methods**			
Study design	4	Present key elements of study design early in the paper	5
Setting	5	Describe the setting, locations, and relevant dates, including periods of recruitment, exposure, follow-up, and data collection	6
Participants	6	(*a*) *Cohort study*—Give the eligibility criteria, and the sources and methods of selection of participants. Describe methods of follow-up<br>*Case-control study*—Give the eligibility criteria, and the sources and methods of case ascertainment and control selection. Give the rationale for the choice of cases and controls<br>*Cross-sectional study*—Give the eligibility criteria, and the sources and methods of selection of participants	6
		(*b*) *Cohort study*—For matched studies, give matching criteria and number of exposed and unexposed<br>*Case-control study*—For matched studies, give matching criteria and the number of controls per case	
Variables	7	Clearly define all outcomes, exposures, predictors, potential confounders, and effect modifiers. Give diagnostic criteria, if applicable	5
Data sources/ measurement	8*	For each variable of interest, give sources of data and details of methods of assessment (measurement). Describe comparability of assessment methods if there is more than one group	5
Bias	9	Describe any efforts to address potential sources of bias	7
Study size	10	Explain how the study size was arrived at	8
Quantitative variables	11	Explain how quantitative variables were handled in the analyses. If applicable, describe which groupings were chosen and why	6
Statistical methods	12	(*a*) Describe all statistical methods, including those used to control for confounding	6
		(*b*) Describe any methods used to examine subgroups and interactions	na
		(*c*) Explain how missing data were addressed	8
		(*d*) *Cohort study*—If applicable, explain how loss to follow-up was addressed<br>*Case-control study*—If applicable, explain how matching of cases and controls was addressed<br>*Cross-sectional study*—If applicable, describe analytical methods taking account of sampling strategy	na
		(*e*) Describe any sensitivity analyses	7

Continued on next page

For peer review only - http://bmjopen.bmj.com/site/about/guidelines.xhtml

**Results**			
Participants	13*	(a) Report numbers of individuals at each stage of study—eg numbers potentially eligible, examined for eligibility, confirmed eligible, included in the study, completing follow-up, and analysed	8
		(b) Give reasons for non-participation at each stage	8
		(c) Consider use of a flow diagram	Figure S1
Descriptive data	14*	(a) Give characteristics of study participants (eg demographic, clinical, social) and information on exposures and potential confounders	Table 1, Table S1
		(b) Indicate number of participants with missing data for each variable of interest	Table 1
		(c) *Cohort study*—Summarise follow-up time (eg, average and total amount)	
Outcome data	15*	*Cohort study*—Report numbers of outcome events or summary measures over time	
		Case-control study—Report numbers in each exposure category, or summary measures of exposure	
		Cross-sectional study—Report numbers of outcome events or summary measures	Table 1
Main results	16	(*a*) Give unadjusted estimates and, if applicable, confounder-adjusted estimates and their precision (eg, 95% confidence interval). Make clear which confounders were adjusted for and why they were included	Table 2, Table S3
		(*b*) Report category boundaries when continuous variables were categorized	All tables
		(*c*) If relevant, consider translating estimates of relative risk into absolute risk for a meaningful time period	na
Other analyses	17	Report other analyses done—eg analyses of subgroups and interactions, and sensitivity analyses	Table S2, S3
**Discussion**			
Key results	18	Summarise key results with reference to study objectives	11
Limitations	19	Discuss limitations of the study, taking into account sources of potential bias or imprecision. Discuss both direction and magnitude of any potential bias	12
Interpretation	20	Give a cautious overall interpretation of results considering objectives, limitations, multiplicity of analyses, results from similar studies, and other relevant evidence	12
Generalisability	21	Discuss the generalisability (external validity) of the study results	12
**Other information**			
Funding	22	Give the source of funding and the role of the funders for the present study and, if applicable, for the original study on which the present article is based	29

*Give information separately for cases and controls in case-control studies and, if applicable, for exposed and unexposed groups in cohort and cross-sectional studies.

Note: An Explanation and Elaboration article discusses each checklist item and gives methodological background and published examples of transparent reporting. The STROBE checklist is best used in conjunction with this article (freely available on the Web sites of PLoS Medicine at http://www.plosmedicine.org/, Annals of Internal Medicine at

For peer review only

http://www.annals.org/, and Epidemiology at http://www.epidem.com/). Information on the STROBE Initiative is available at www.strobe-statement.org.

For peer review only

BMJ Open

Spatial epidemiology of gestational age and birth weight in Switzerland: Census-based linkage study

Journal:	*BMJ Open*
Manuscript ID	bmjopen-2018-027834.R1
Article Type:	Original research
Date Submitted by the Author:	12-Jun-2019
Complete List of Authors:	Skrivankova, Veronika; Universitat Bern Institut fur Sozial- und Praventivmedizin,<br>Zwahlen , Marcel; Universitat Bern Institut fur Sozial- und Praventivmedizin<br>Adams, Mark; University of Zurich, Department of Neonatology<br>Low, Nicola; University of Bern, Institute of Social and Preventive Medicine<br>Kuehni, Claudia; Universitat Bern Institut fur Sozial- und Praventivmedizin<br>Egger, Matthias; University of Bern, Institute of Social and Preventive Medicine
**Primary Subject Heading**:	Public health
Secondary Subject Heading:	Reproductive medicine, Obstetrics and gynaecology
Keywords:	birth weight, gestational age, spatial epidemiology, Switzerland, socio-economic position, education

SCHOLARONE™
Manuscripts

Spatial epidemiology of gestational age and birth weight in Switzerland: Census-based linkage study

Veronika Skrivankova[1], Marcel Zwahlen[1], Mark Adams[2], Nicola Low[1],
Claudia E Kuehni[1,3], Matthias Egger[1]

1) Institute of Social and Preventive Medicine (ISPM), University of Bern, Bern, Switzerland

2) Department of Neonatology, University Hospital Zurich, University of Zurich, Zurich, Switzerland

3) Pediatric Respiratory Medicine, Inselspital, Bern University Hospital, University of Bern, Switzerland

Correspondence to:

Professor Matthias Egger MD

Institute of Social and Preventive Medicine (ISPM), University of Bern

Mittelstrasse 43, CH-3012 Bern, Switzerland

matthias.egger@ispm.unibe.ch

Word counts: Abstract 245 words, strengths and limitations of the study 129 words, main text 3484 words, 44 references, 3 tables, 2 figures, supplementary materials with 4 tables and 3 figures.

For peer review only - http://bmjopen.bmj.com/site/about/guidelines.xhtml

ABSTRACT

Background: Gestational age and birth weight are strong predictors of infant morbidity and mortality. Understanding spatial variation can inform policies to reduce health inequalities. We examined small-area variation in gestational age and birth weight in Switzerland.

Methods: All singleton live births recorded in the Swiss Live Birth Register 2011 to 2014 were eligible. We deterministically linked the Live Birth Register with census and survey data to create datasets including neonatal and pregnancy-related variables, parental characteristics and geographical variables. We produced maps of 705 areas and fitted linear mixed-effect models to assess to what extent spatial variation was explained by these variables.

Results: We analysed all 315,177 eligible live births. Area-level averages of gestational age varied between 272-279 days, and between 3138-3467g for birth weight. The fully adjusted models explained 31% and 87% of spatial variation of gestational age and birth weight, respectively. Language region explained most of the variation, with shorter gestational age and lower birth weight in French- and Italian- than in German-speaking areas. Other variables explaining variation were, for gestational age, the level of urbanisation, the parents' nationality and missing father. For birth weight, they were gestational age, altitude, and parental nationality. In a random sample of 81,968 live births with data on parental education, levels of education were only weakly associated with gestational age or birth weight.

Conclusions: In Switzerland, small area variation in birth weight is largely explained, and variation in gestational age partially explained, by geocultural, socio-demographic and pregnancy factors.

For peer review only

Strengths and limitations of this study

- This study was based on a large sample with national coverage, with data on neonatal and pregnancy-related predictors of gestational age and birth weight, and precise spatial data.
- No data were available on the mode of delivery, maternal smoking, mothers' weight and height or gestational diabetes.
- The fully adjusted model explained over 80% of the regional variation in birth weight and about 30% of the variation in gestational age.
- Language region, a proxy for cultural, social and behavioural factors, was a strong explanatory factor, with lower birth weight and shorter gestation in the French and Italian compared to the German language region.
- Unknown father was associated with shorter gestation and lower birth weight, indicating that children not recognised by their fathers may be at higher risk of poor outcomes.

For peer review only - http://bmjopen.bmj.com/site/about/guidelines.xhtml

INTRODUCTION

Gestational age and birth weight are important indicators of prenatal development and predictors of infant morbidity, mortality and long-term health [1–4]. An understanding of geographic differences and their determinants can help to develop policies that reduce health inequalities across population groups and regions [1–4]. Many genetic, physiological, pregnancy-related, socio-economic, lifestyle and environmental factors have been reported to influence gestational age and birth weight [5–8]. Some of these factors tend to cluster in space and regional differences in health outcomes may hence be partially explained by the spatial distribution of their predictors. Importantly, both individual-level factors and the social and environmental characteristics of communities and neighbourhoods may contribute to regional differences [9,10].

Variation across small areas in pregnancy outcomes have not been studied widely. In Scotland, small area crime rates were associated with lower birth weight and with the risk of both small for gestational age babies and preterm birth [11]. A study at county level in Georgia and South Carolina in the United States showed that the proportion of African Americans was associated with low birth weight, whereas higher income was associated with higher birth weight [12]. Similarly, neighbourhood racial composition contributed to variation in low birth weight in New York State [13]. Other small-area analyses have examined associations between birth outcomes and air pollution [14,15]. To our knowledge, few small-area analyses have considered gestational age.

In Switzerland, studies of pregnancy outcomes have focused on specific groups such as migrants or HIV-infected women [16,17], but have not examined geographic variations. The Federal Office of Statistics publishes routine statistics from the Live Birth Register, which does not include geographic information [18]. The objectives of this study were to conduct a nationwide analysis of spatial variation in gestational age and birth weight, and to assess how much small-area variation was explained by available data about neonatal and pregnancy-related variables, parental characteristics and geographical variables.

For peer review only - http://bmjopen.bmj.com/site/about/guidelines.xhtml

METHODS

Data sources

We used deterministic methods to link three data sources using encrypted national identification numbers: the Live Birth Register, the Swiss National Cohort and the Structural Surveys. Registration of live births is compulsory by law in Switzerland coverage is near 100%. The Swiss National Cohort (SNC) is a long-term, national study of mortality in Switzerland [19,20], linking census and mortality records. The 1990 and 2000 censuses were the last house-to-house censuses with coverage of the entire Swiss population. From 2010 onwards, the national census was replaced by a national population register and annual postal survey of the resident population, known as Structural Surveys [21]. Each structural Survey includes a random sample of around 300,000 people aged 15 years or older; for example, in 2010, it included 317,221 persons [21]. The reference is the entire Swiss resident population and the reference day 31 December.

Variables and definitions

We defined three sets of variables. The first set, neonatal and pregnancy-related variables come from the Live Birth Register; date of birth, birth weight, gestational age, sex and birth rank. Birth weight is measured after initial mother-child bonding, usually by the midwife using a calibrated hospital scale. Gestational age is based on the last menstrual period, with or without additional information from ultrasound scans. Birth rank was calculated from the list of all live births by the same mother recorded in the Live Birth Register, and is hence restricted to the births that occurred in Switzerland. It was classified as 1, 2, 3 and ≥4 live births, including the current birth. The second set includes parental variables. The Structural Surveys provide information about the highest level of completed maternal and paternal education, classified as ‘tertiary’, ‘secondary’, or ‘compulsory or less’. The Swiss National Cohort provides data about parental nationality categorised as ‘Swiss’, ‘Southern Europe’, ‘Western Europe’, ‘Northern Europe’, ‘Eastern Europe’, ‘Other’ (non-European), or missing (supplementary Table S1 gives the full list of countries). The third set, geographical variables comes from the Swiss National Cohort. Each live birth was assigned an altitude and one of 705 statistical areas [22], based on the geocode of place of residence of the mother at the time of birth. Language regions are ‘German’, ‘French’ and ‘Italian’, and the level of urbanisation was defined using standard definitions of ‘urban’, ‘peri-urban’ and ‘rural’.

For peer review only - http://bmjopen.bmj.com/site/about/guidelines.xhtml

Study populations and outcomes

All singleton live births recorded in the Live Birth Register from 1 January 2011 to 31 December 2014 were eligible. Gestational age at birth and birth weight were the outcomes of interest. For each outcome, two datasets were analysed: the first, larger dataset consisted of all eligible births with complete data on gestational age, birth weight and nationality of the mother. The second was the complete case population containing eligible live births with available data on all variables, including parental education. The second dataset hence included only newborns whose parents were included in the random sample of one of the Structural Surveys 2010-2014. We also excluded mothers who delivered at age less than 20 years, because education is incomplete at that age.

Statistical and spatial analyses

We fitted linear mixed-effect models (LMEM) to examine the associations between the two outcomes and the neonatal and pregnancy, parental and environmental factors. In the model for birth weight, we log-transformed the outcome and used a cubic spline function with three knots at weeks 25, 30 and 35 to capture the relationship between gestational age and log birth weight. Log transforming the birth weight results in a multiplicative model. Except for gestational age, maternal age and altitude, all predictors were modelled categorically. Maternal age was modelled by a piece-wise linear function, with age group 20 to 30 years as the reference group and separate linear trends for age groups 30-40 years, over 40 years and less than 20 years. Altitude was centred at 500 m and modelled linearly. The random effects in the mixed-effect model captured area-level differences between observed and expected mean outcome, based on the 705 statistical areas [22]. In the main analysis, we fitted four models to the complete-case dataset: Model 0 contained no explanatory variables. Model 1 included birth and pregnancy-related variables: sex, birth rank and gestational age (for the analysis of birth weight). Model 2 additionally included age of the mother, parental education and nationality. Model 3 additionally included geographical variables: altitude, degree of urbanisation and language region.

We displayed mean gestational age and birth weight at area-level on maps and assessed to what extent spatial variation was accounted for by the explanatory variables. Values were categorised into seven intervals symmetric around the mean and color-coded. Spatial autocorrelation of the gestational age and birth weight across regions was tested by global and local Moran's I tests [23]. The global Moran test summarises overall spatial

For peer review only - http://bmjopen.bmj.com/site/about/guidelines.xhtml

autocorrelation and the local test identifies areas that are correlated with neighbouring areas. In the presence of spatial autocorrelation, model estimates are at risk of bias if the autocorrelation is not taken into account.

We performed three sensitivity analyses. First, we accounted for spatial autocorrelation using the Besag-York-Mollier (BYM) model [24] using uninformative gamma-distributed (1, 0.005) priors. The calculations were carried out using the Integrated Nested Laplace Approximation (INLA) approach [25]. Similar results from models with and without the spatial component indicate low bias. Second, we repeated analyses of birth weight without adjusting for gestational age. Third we repeated analyses of birth weight and gestational age, additionally adjusting for neighbourhood socio-economic position (SEP), using an updated version of the Swiss SEP index, which is based on levels of rent, education and occupation of heads of households and crowding [26]. The updated version of the index is based on data from Structural Surveys 2010-2014, and includes information on income of households in the neighbourhood. We used quintiles of the index in the analysis, with higher quintiles indicating higher SEP.

All analyses and maps were done in R 3.3.2 [27] using packages lme4, maptools, sp, spdep, rgdal, INLA, GISTools, rgeos, raster and ggplot2.

Patient and public involvement

This analysis was based on routine registry data and no patients were involved in developing the research question, outcome measures and overall design of the study. Due to the anonymous nature of the data, we were unable to disseminate the results of the research directly to study participants.

For peer review only - http://bmjopen.bmj.com/site/about/guidelines.xhtml

RESULTS

Characteristics of study populations

A total of 328,349 live births were recorded in Switzerland between 1 January 2011 and 31 December 2014. We excluded non-singleton live births (n=11,835) and those with missing gestational age, birth weight or maternal nationality. The eligible study population therefore included 315,177 singleton live births. The complete case population consisted of 81,968 singleton live births with values available for all predictors including parental education, for which complete data were only available in the Structural Surveys (supplementary Figure S1).

Table 1 shows the distributions of predictors and outcomes in the two study populations. Data about the nationality of fathers was missing for 1.5% of eligible live births. In almost all of these cases, information about the father was missing completely, indicating that the father is unknown to the authorities. Apart from missing data, the distributions of most variables were similar between the two nested datasets. The proportion of Swiss mothers and fathers was higher in the complete case population than in the eligible population. Birth at full term is defined as between 39 and 41 weeks of gestation (273 to 287 days). The mean gestational age in the eligible population was 276 days (SD 12) and the mean birth weight 3328 g (SD 515). The corresponding figures in the complete case population were 276 days (SD 12) and 3339 g (SD 501).

Maps of gestational age and birth weight

Figure 1 presents maps of Switzerland with crude average gestational age and birth weight across the 705 areas. For both outcomes, the maps are broadly similar between the eligible and complete case populations. For gestational age, area-level averages for the eligible population vary between 272 and 279 days. For the complete case population variation was greater, from 268 to 281 days, as expected for a smaller sample. The map shows shorter gestation in the Western, North Western region and Southern (Canton of Ticino) regions of Switzerland, with a patchy pattern in the densely populated areas between the Alps (across the centre) and Jura mountain ranges (to the North West). For birth weight, area-level averages vary between 3138 and 3467g for the eligible population and between 3080 and 3648 g for the complete case population. The maps for birth weight show lower birth weights in the Western and Southern regions of the country. The French and Italian-speaking

For peer review only - http://bmjopen.bmj.com/site/about/guidelines.xhtml

regions are in the West and South of Switzerland, with the remainder being German-speaking.

Multivariable analyses

Table 2 shows associations of area-level mean gestational age at birth and mean birth weight with pregnancy, parental and environmental factors from the fully adjusted linear mixed-effects models (model 3). For gestational age, the largest differences were observed across maternal age at birth. Compared to maternal age 20-30 years, gestational age was considerably shorter in teenage mothers, and in mothers aged over 40 years. For example, in mothers aged 15 years, pregnancies were about 4 days shorter, and after age of 40 years, they were about 3 days shorter for each 5-year increase in maternal age. Compared with Swiss fathers, pregnancies were about 4 days shorter if the nationality of the father was missing. Smaller differences in gestational age were observed across categories of sex, birth rank, nationality of the mother, urbanisation and between language regions (Table 2). In the complete case population, lower levels of education were associated with shorter pregnancies. Gestational age at birth was not associated with altitude.

Supplementary Figure S2 shows the relationship between gestational age and birth weight separately for male and female newborns. Male newborns were about 5% heavier than female newborns and birth weight increased with birth order (Table 2). In contrast to gestational age, mother's age was not associated with birth weight. Babies born to mothers or fathers from Northern or Eastern Europe were slightly heavier than babies born to Swiss mothers; birth weights were lowest for babies of fathers with missing nationality. Birth weight slightly decreased with increasing parental educational attainment. Babies born in the French and Italian-speaking regions were lighter than babies born in the German-speaking Switzerland. Finally, birth weight decreased with increasing altitude of residence.

Proportion of spatial variation explained

The fully adjusted model (model 3) for gestational age explained 31% and 39% of the spatial variation across the 705 areas for eligible and complete case populations, respectively. The corresponding figures for birth weight were 87% and 88%. When assessing each factor separately (Table 3), language region alone explained most of the spatial variation for both outcomes. For gestational age, level of urbanisation of the mother's place of residence also explained a considerable part of the variation. Factors that contributed to explaining the

For peer review only - http://bmjopen.bmj.com/site/about/guidelines.xhtml

spatial variation in birth weight were gestational age, parental nationalities, altitude at the mother's place of residence and birth order. Figure 2 illustrates the reduction in the spatial variation of gestational age and birth weight with maps, moving from model 0 (0% reduction) to models 1, 2 and 3, based on the complete case population.

Spatial autocorrelation and sensitivity analyses

For gestational age, the global Moran's I statistic, based on the complete case dataset and model 0, was I=0.19, with $P<10^{-14}$. After adjusting for all the predictors in model 3 there was still some residual autocorrelation (*I*=0.10, P=0.0004). For birth weight, the corresponding Moran's I statistic was I=0.28, with $P<10^{-15}$. After adjusting for all predictors in model 3 there was little residual autocorrelation (I=0.04, P=0.051). Supplementary Table S2 compares the results from model 3 accounting and not accounting for spatial autocorrelation. The results are similar and the potential bias from residual spatial autocorrelation is therefore unlikely to be a major issue. Repeating analyses of birth weight without adjusting for gestational age produced generally similar coefficients (supplementary Table S3). Associations with maternal age, maternal education and language regions were slightly stronger in model 3 without adjustment for gestational age, possibly because some of their effect was mediated by gestational age. Model 3 without gestational age explained 77% and 76% of the spatial variation in the eligible and complete case population, respectively. The index of neighbourhood SEP was only weakly associated with the two outcomes (Supplementary Table S4), and adjusting for it only slightly increased the amount of spatial variation explained.

For peer review only

For peer review only - http://bmjopen.bmj.com/site/about/guidelines.xhtml

DISCUSSION

Our study assessed factors associated with gestational age and birth weight in Switzerland and their contribution to spatial variation, based on routinely collected data. Gestational age at birth was strongly associated with maternal age, missing information on the father and language region. Birth weight was associated with sex, birth rank, missing information on the father, parental education, altitude and language region. The variables included in the fully adjusted model explained more than 80% of the regional variation in birth weight and about 30% of the regional variation in gestational age. Strengths of this study include a large sample with national coverage of the Swiss resident population, as well as the availability of data on several relevant predictors, either on all births or on a large random sample of eligible births. Precise spatial data and spatial statistics allowed us to assess the proportion of area-level variation explained, spatial autocorrelation and gauge the likelihood of bias due to residual autocorrelation.

This study found important spatial variation in both gestational age and birth weight in Switzerland. Language region in Switzerland was the single factor that explained the greatest proportion of spatial variation in gestational age and birth weight. In the French and Italian speaking regions, gestational age was shorter and birth weight lower than in the German speaking part. Language region is a proxy for a wide range of cultural, social and behavioural factors, including diet, smoking and alcohol consumption [28] of parents, as well as their ancestry. In this context it is noteworthy that neighbourhood SEP explained only a small proportion of the spatial variation.

Other factors that could not be measured directly, such as health care provision, might have accounted for some of the unexplained variation. In particular, data at the individual or small area level on the mode of delivery (vaginal or by Caesarean section, induced or spontaneous) were not available. The proportion of live births with Caesarian section as the mode of delivery varies across regions in Switzerland, and it is reasonable to expect that it would explain some of the remaining variation, both in gestational age and birth weight. Specifically, we would expect regions with higher proportions of Caesarian section to have lower mean gestational age (and consequently birthweight). However, the regional rates of Caesarian section published by the Federal Office of Statistics do not match this expectation [29], with urban areas showing some of the highest Caesarian section rates

For peer review only - http://bmjopen.bmj.com/site/about/guidelines.xhtml

but also high mean gestational age and birth weight. In fact, geographical patterns of Caesarean section seem to be largely driven by urban-rural differences. Differences in section rates may have contributed to spatial variation in gestational age, but it seems unlikely that they are an important driver of this variation.

While young and old maternal age are well-known predictors of shorter gestation [30,31], the association we found with missing data on the father's nationality was unexpected. In the vast majority of cases, the information is missing because no father came forward and officially accepted paternity of the child. It is possible that missing data about the father are an indicator of lower socio-economic position and social support of the mother, resulting in greater vulnerability. Studies from the United States of America found a missing name of the father on the infant's birth certificate was associated with lower education, smoking during pregnancy, preterm birth, lower birth weight, no breastfeeding and higher neonatal and post-neonatal mortality [32–35]. Children not recognised by their fathers may thus be a group at higher risk of infant and child morbidity and mothers might benefit from additional care during pregnancy and postnatally.

There are several limitations to our study. We did not have data about maternal health-related behaviours such as smoking [36], mothers' weight and height [36], disease such as gestational diabetes and data on parental genetic factors. Whilst parental nationality and education might have served as crude proxies for some missing variables, individual-level data about these factors would be valuable. A recent large-scale meta-analysis of genome-wide association data indicated that genetic factors influence birth weight through their effects on gestational age, maternal glucose metabolism, cytochrome P450 activity and possibly through effects on maternal immune function and blood pressure [37]. Of note, compared to the foetus who carries maternal and paternal genes, maternal genes exert a larger effect on gestational age and a weaker effect on birth weight [38,39].

Our study also showed associations between mean gestational age and the proportion of preterm births (<37 weeks), as well as mean birth weight and proportion of low birth weight newborns (<2500 g) across the 705 small areas, i.e. associations with conditions that are clinically relevant (Figure S3). However, from a statistical point of view, analyzing means is more robust and powerful than using a binary indicator defined by a cutoff [40]. Finally, we adjusted analyses of birth weight for gestational age, which may

For peer review only - http://bmjopen.bmj.com/site/about/guidelines.xhtml

mediate the effects of other variables, for example maternal age. Adjusting for a variable on the causal pathway has been criticised because it may introduce selection bias (or collider bias in the language of directed acyclic graphs), if there are unknown or unmeasured factors that have an effect on both gestational age and birth weight [41–43]. In this study, results were broadly similar with and without adjustment for gestational age and our focus was not on causal inference, but on gaining an understanding of the factors contributing to spatial variation of birth weight and gestational age.

In conclusion, our study identified important differences in mean gestational age and birth weight across Switzerland. Small area variation in birth weight is largely, and variation in gestational age partially explained by pregnancy-related, parental, and environmental factors.

For peer review only

For peer review only - http://bmjopen.bmj.com/site/about/guidelines.xhtml

REFERENCES

1 Regev RH, Lusky A, Dolfin T, *et al.* Excess mortality and morbidity among small-for-gestational-age premature infants: a population-based study. *J Pediatr* 2003;**143**:186–91.

2 Wilcox AJ. Birth weight and perinatal mortality: the effect of maternal smoking. *Am J Epidemiol* 1993;**137**:1098–104.

3 Barker DJ, Winter PD, Osmond C, *et al.* Weight in infancy and death from ischaemic heart disease. *Lancet* 1989;**ii**:577–80.http://www.ncbi.nlm.nih.gov/pubmed/2570282 (accessed 3 Feb 2015).

4 Lubchenco LO, Searls DT, Brazie J V. Neonatal mortality rate: relationship to birth weight and gestational age. *J Pediatr* 1972;**81**:814–22.

5 de Bernabé J V., Soriano T, Albaladejo R, *et al.* Risk factors for low birth weight: a review. *Eur J Obstet Gynecol Reprod Biol* 2004;**116**:3–15.

6 Kramer MS. Determinants of low birth weight: methodological assessment and meta-analysis. *Bull World Health Organ* 1987;**65**:663–737.

7 Secker-Walker RH, Vacek PM. Relationships between cigarette smoking during pregnancy, gestational age, maternal weight gain, and infant birthweight. *Addict Behav* 2003;**28**:55–66. doi:10.1016/S0306-4603(01)00216-7

8 Matoba N, Yu Y, Mestan K, *et al.* Differential Patterns of 27 Cord Blood Immune Biomarkers Across Gestational Age. *Pediatrics* 2009;**123**:1320–8. doi:10.1542/peds.2008-1222

9 Diez Roux A V, Mair C. Neighborhoods and health. *Ann N Y Acad Sci* 2010;**1186**:125–45. doi:10.1111/j.1749-6632.2009.05333.x

10 Macintyre S, Ellaway A, Cummins S. Place effects on health: how can we conceptualise, operationalise and measure them? *Soc Sci Med* 2002;**55**:125–39.http://www.ncbi.nlm.nih.gov/pubmed/12137182

11 Clemens T, Dibben C. Living in stressful neighbourhoods during pregnancy: An observational study of crime rates and birth outcomes. *Eur J Public Health* 2017;**27**:197–202. doi:10.1093/eurpub/ckw131

12 Kirby RS, Liu J, Lawson AB, *et al.* Spatio-temporal patterning of small area low birth weight incidence and its correlates: A latent spatial structure approach. *Spat Spatiotemporal Epidemiol* 2011;**2**:265–71. doi:10.1016/j.sste.2011.07.011

For peer review only - http://bmjopen.bmj.com/site/about/guidelines.xhtml

13 Insaf TZ, Talbot T. Identifying areas at risk of low birth weight using spatial epidemiology: A small area surveillance study. *Prev Med (Baltim)* 2016;**88**:108–14. doi:10.1016/j.ypmed.2016.03.019

14 Dibben C, Clemens T. Place of work and residential exposure to ambient air pollution and birth outcomes in Scotland, using geographically fine pollution climate mapping estimates. *Environ Res* 2015;**140**:535–41. doi:10.1016/j.envres.2015.05.010

15 Ghosh JKC, Wilhelm M, Su J, *et al.* Assessing the Influence of Traffic-related Air Pollution on Risk of Term Low Birth Weight on the Basis of Land-Use-based Regression Models and Measures of Air Toxics. *Am J Epidemiol* 2012;**175**:1262–74. doi:10.1093/aje/kwr469

16 Aebi-Popp K, Lapaire O, Glass TR, *et al.* Pregnancy and delivery outcomes of HIV infected women in Switzerland 2003-2008. *J Perinat Med* 2010;**38**:353–8. doi:10.1515/JPM.2010.052

17 Jaeger FN, Hossain M, Kiss L, *et al.* The health of migrant children in Switzerland. *Int J Public Health* 2012;**57**:659–71. doi:10.1007/s00038-012-0375-8

18 Federal Statistical Office. Births. https://www.bfs.admin.ch/bfs/de/home/statistiken/bevoelkerung/geburten-todesfaelle/geburten.html (accessed 18 Sep 2018).

19 Spörri A, Zwahlen M, Egger M, *et al.* The Swiss National Cohort: a unique database for national and international researchers. *Int J Public Heal* 2010;**55**:239–42. doi:10.1007/s00038-010-0160-5

20 Bopp M, Spörri A, Zwahlen M, *et al.* Cohort Profile: the Swiss National Cohort--a longitudinal study of 6.8 million people. *Int J Epidemiol* 2009;**38**:379–84. doi:10.1093/ije/dyn042

21 Federal Statistical Office. Structural Survey. https://www.bfs.admin.ch/bfs/en/home/statistics/population/surveys/se.html (accessed 18 Sep 2018).

22 Federal Statistical Office. Swiss MedStat regions. https://www.bfs.admin.ch/bfs/de/home/statistiken/gesundheit/nomenklaturen/medsreg.html (accessed 18 Sep 2018).

23 Moran PAP. Notes on Continuous Stochastic Phenomena. *Biometrika* 1950;**37**:17–23.http://www.jstor.org/stable/2332142

For peer review only - http://bmjopen.bmj.com/site/about/guidelines.xhtml

24 Besag J, York J, Mollié A. Bayesian image restoration, with two applications in spatial statistics. *Ann Inst Stat Math* 1991;:1–20.

25 Rue H, Martino S, Chopin N. Approximate Bayesian inference for latent Gaussian models by using integrated nested Laplace approximations. *J R Stat Soc Ser B* 2009;**71**:319–92.

26 Panczak R, Galobardes B, Voorpostel M, *et al.* A Swiss neighbourhood index of socioeconomic position: development and association with mortality. *J Epidemiol Community Health* 2012;**66**:1129–36. doi:10.1136/jech-2011-200699

27 The R Core Team: R. A Language and Environment for Statistical Computing. Vienna, Austria. 2014;**ISBN 3-900**.http://www.r-project.org.

28 Faeh D, Minder C, Gutzwiller F, *et al.* Culture, risk factors and mortality: can Switzerland add missing pieces to the European puzzle? *J Epidemiol Community Heal* 2009;**63**:639–645.

29 Federal Office of Statistics. Rate of Caeserian section (MS regions). https://www.bfs.admin.ch/bfs/en/home/statistics/catalogues-databases/maps.assetdetail.4262550.html

30 Kozuki N, Lee AC, Silveira MF, *et al.* The associations of parity and maternal age with small-for-gestational-age, preterm, and neonatal and infant mortality: A meta-analysis. BMC Public Health. 2013;**13**:S2. doi:10.1186/1471-2458-13-S3-S2

31 Restrepo-Méndez MC, Lawlor DA, Horta BL, *et al.* The association of maternal age with birthweight and gestational age: A cross-cohort comparison. *Paediatr Perinat Epidemiol* 2015;**29**:31–40. doi:10.1111/ppe.12162

32 Alio AP, Mbah AK, Kornosky JL, *et al.* Assessing the impact of paternal involvement on racial/ethnic disparities in infant mortality rates. *J Community Health* 2011;**36**:63–8. doi:10.1007/s10900-010-9280-3

33 Gaudino JA, Jenkins B, Rochat RW. No fathers' names: A risk factor for infant mortality in the State of Georgia, Usa. *Soc Sci Med* 1999;**48**:253–65. doi:10.1016/S0277-9536(98)00342-6

34 Cheng ER, Hawkins SS, Rifas-Shiman SL, *et al.* Association of missing paternal demographics on infant birth certificates with perinatal risk factors for childhood obesity. *BMC Public Health* 2016;**16**:1–10. doi:10.1186/s12889-016-3110-1

35 Tan H, Wen SW, Walker M, *et al.* Missing paternal demographics: A novel indicator for

For peer review only

identifying high risk population of adverse pregnancy outcomes. *BMC Pregnancy Childbirth* 2004;**4**. doi:10.1186/1471-2393-4-21

36 Wen SW, Goldenberg RL, Cutter GR, *et al.* Smoking, maternal age, fetal growth, and gestational age at delivery. *Am J Obstet Gynecol* 1990;**162**:53–8. doi:10.1016/0002-9378(90)90819-S

37 Beaumont RN, Warrington NM, Cavadino A, *et al.* Genome-wide association study of offspring birth weight in 86 577 women identifies five novel loci and highlights maternal genetic effects that are independent of fetal genetics. *Hum Mol Genet* 2018;**27**:742–56. doi:10.1093/hmg/ddx429

38 Beaty TH. Invited commentary: Two studies of genetic control of birth weight where large data sets were available. *Am J Epidemiol* 2007;**165**:753–5. doi:10.1093/aje/kwk106

39 Lunde A, Melve KK, Gjessing HK, *et al.* Genetic and environmental influences on birth weight, birth length, head circumference, and gestational age by use of population-based parent-offspring data. *Am J Epidemiol* 2007;**165**:734–41. doi:10.1093/aje/kwk107

40 Altman DG, Royston P. The cost of dichotomising continuous variables. *BMJ* 2006;**332**:1080.

41 Cole SR, Platt RW, Schisterman EF, *et al.* Illustrating bias due to conditioning on a collider. *Int J Epidemiol* 2010;**39**:417–20. doi:10.1093/ije/dyp334

42 Delbaere I, Vansteelandt S, De Bacquer D, *et al.* Should we adjust for gestational age when analysing birth weights? The use of z-scores revisited. *Hum Reprod* 2007;**22**:2080–3. doi:10.1093/humrep/dem151

43 Wilcox AJ, Weinberg CR, Basso O. On the pitfalls of adjusting for gestational age at birth. *Am J Epidemiol* 2011;**174**:1062–8. doi:10.1093/aje/kwr230

For peer review only

For peer review only - http://bmjopen.bmj.com/site/about/guidelines.xhtml

Table 1. Characteristics of complete case and eligible study populations.

	Eligible population			Complete case population		
		Gest. age (days)	Birth weight (g)		Gest. age (days)	Birth weight (g)
	No. (%)	Mean (SD)	Mean (SD)	No. (%)	Mean (SD)	Mean (SD)
**Total**	315'177 (100%)	276 (12)	3328 (515)	81'968 (100%)	276 (12)	3339 (501)
**Birth weight (g)**						
<1500	2141 (0.7%)	196 (27)	966 (354)	445 (0.5%)	198 (28)	983 (491)
1500-1999	2413 (0.8%)	238 (15)	1800 (142)	612 (0.7%)	239 (15)	1803 (528)
2000-2499	10036 (3.2%)	258 (14)	2312 (134)	2484 (3%)	258 (13)	2314 (477)
≥ 2500	300586 (95.4%)	277 (9)	3391 (423)	78426 (95.7%)	277 (9)	3396 (502)
**Gestation. age (weeks)**						
$<32^0$	2333 (0.7%)	195 (23)	1108 (527)	487 (0.6%)	196 (23)	1134 (491)
32^0-34^6	3950 (1.3%)	237 (6)	2144 (424)	961 (1.2%)	237 (6)	2136 (470)
35^0-36^6	10907 (3.5%)	253 (4)	2686 (431)	2760 (3.4%)	253 (4)	2692 (489)
$\geq 37^0$	297987 (94.5%)	278 (8)	3385 (440)	77760 (94.9%)	278 (8)	3390 (502)
**Sex**						
Female	152757 (48.5%)	276 (12)	3260 (494)	39823 (48.6%)	276 (11)	3267 (502)
Male	162420 (51.5%)	275 (13)	3392 (525)	42145 (51.4%)	276 (12)	3406 (501)
**Birth rank**						
1	155739 (49.4%)	276 (13)	3262 (519)	37763 (46.1%)	276 (13)	3267 (498)
2	115440 (36.6%)	275 (11)	3382 (497)	32315 (39.4%)	276 (11)	3386 (504)
3	34364 (10.9%)	275 (11)	3418 (509)	9360 (11.4%)	275 (11)	3430 (508)
≥ 4	9634 (3.1%)	275 (12)	3438 (537)	2530 (3.1%)	275 (11)	3459 (498)
**Civil status**						
Married	250055 (79.3%)	276 (12)	3345 (508)	69465 (84.7%)	276 (12)	3349 (501)
Not married	65122 (20.7%)	276 (14)	3262 (536)	12503 (15.3%)	276 (13)	3283 (503)
**Maternal age (years)**						
mean (SD)	*31.7 (5.0)*			*32.2 (4.7)*		
< 20	2679 (0.8%)	275 (16)	3224 (554)	0 (0%)	-	-
≥ 20-25	28615 (9.1%)	277 (12)	3317 (511)	5417 (6.6%)	277 (12)	3337 (491)
≥ 25-30	82620 (26.2%)	276 (12)	3330 (506)	20771 (25.3%)	276 (12)	3337 (500)
≥ 30-35	118303 (37.5%)	276 (12)	3335 (510)	32771 (40%)	276 (12)	3341 (505)
≥ 35-40	67914 (21.5%)	275 (12)	3333 (523)	19052 (23.2%)	275 (11)	3345 (497)
≥ 40	15046 (4.8%)	273 (14)	3286 (555)	3957 (4.8%)	273 (14)	3295 (512)
**Nationality mother**						
Switzerland	194570 (61.7%)	276 (12)	3322 (511)	55591 (67.8%)	276 (12)	3331 (502)
Southern Europe	23585 (7.5%)	275 (12)	3251 (494)	5761 (7%)	276 (11)	3261 (502)
Western Europe	26005 (8.3%)	276 (12)	3348 (516)	6495 (7.9%)	276 (12)	3359 (508)
Northern Europe	3695 (1.2%)	276 (13)	3418 (510)	850 (1%)	276 (13)	3414 (508)
Eastern Europe	38762 (12.3%)	276 (13)	3397 (523)	8035 (9.8%)	276 (12)	3422 (499)
Other	28560 (9.1%)	275 (14)	3313 (535)	5236 (6.4%)	275 (13)	3332 (492)
**Nationality father**						
Switzerland	191589 (60.8%)	276 (12)	3329 (506)	55432 (67.6%)	276 (12)	3336 (502)
Southern Europe	31466 (10%)	275 (12)	3256 (493)	7970 (9.7%)	275 (11)	3262 (504)
Western Europe	26954 (8.6%)	276 (12)	3353 (518)	6661 (8.1%)	276 (12)	3367 (514)
Northern Europe	3911 (1.2%)	276 (12)	3406 (510)	887 (1.1%)	276 (13)	3393 (499)
Eastern Europe	35387 (11.2%)	276 (13)	3397 (528)	7229 (8.8%)	276 (12)	3418 (489)
Other	21077 (6.7%)	276 (13)	3307 (531)	3789 (4.6%)	276 (12)	3319 (497)
missing	4793 (1.5%)	272 (23)	3148 (693)	-	-	-
**Education mother**						
Tertiary	42088 (13.4%)	276 (12)	3344 (500)	33505 (40.9%)	276 (12)	3347 (500)
Secondary	48878 (15.5%)	276 (12)	3328 (509)	38382 (46.8%)	276 (12)	3331 (502)
Compulsory	14642 (4.6%)	275 (13)	3329 (534)	10081 (12.3%)	275 (13)	3336 (503)
Unknown (age <20 yrs)	2679 (0.8%)	275 (16)	3224 (554)	0 (0%)	-	-
missing	206890 (65.6%)	276 (12)	3326 (517)	-	-	-
**Education father**						
Tertiary	49848 (15.8%)	276 (12)	3348 (497)	40345 (49.2%)	276 (12)	3350 (500)
Secondary	41301 (13.1%)	276 (12)	3323 (511)	32118 (39.2%)	276 (12)	3327 (504)
Compulsory	13731 (4.4%)	276 (12)	3323 (514)	9505 (11.6%)	276 (12)	3330 (500)
missing	210297 (66.7%)	276 (13)	3325 (519)	-	-	-
**Altitude (m)**						
mean (SD)	*515 (189)*			*511 (180)*		
**Urbanisation**						
Urban	96643 (30.7%)	276 (13)	3326 (517)	22770 (27.8%)	276 (12)	3334 (502)
Peri-urban	138826 (44%)	275 (12)	3329 (514)	36629 (44.7%)	276 (12)	3339 (502)
Rural	79708 (25.3%)	276 (12)	3329 (512)	22569 (27.5%)	276 (12)	3343 (500)
**Language region**						
German	223586 (70.9%)	276 (12)	3348 (515)	54106 (66%)	276 (12)	3362 (502)
French	80068 (25.4%)	275 (12)	3283 (512)	23579 (28.8%)	275 (12)	3296 (501)
Italian	11523 (3.7%)	275 (12)	3252 (494)	4283 (5.2%)	275 (11)	3268 (500)
**Socio-economic position**						
1st quintile	63230 (20.1%)	276 (12)	3318 (522)	15752 (19.2%)	276 (12)	3331 (501)
2nd quintile	63199 (20.1%)	276 (12)	3324 (519)	16034 (19.6%)	276 (12)	3334 (505)
3rd quintile	63156 (20%)	276 (12)	3329 (516)	16555 (20.2%)	276 (12)	3337 (500)
4th quintile	62970 (20%)	276 (12)	3335 (509)	16933 (20.7%)	276 (12)	3344 (500)
5th quintile	62622 (19.9%)	276 (12)	3335 (507)	16694 (20.4%)	276 (12)	3346 (502)

For peer review only - http://bmjopen.bmj.com/site/about/guidelines.xhtml

Table 2. Associations of mean gestational age at birth and mean birth weight with pregnancy, parental and environmental factors from adjusted linear mixed-effects model (model 3).

	Gestational age (days) Absolute differences (95% CI)		Birth weight (g) * Relative differences (95% CI)	
	Eligible population	Complete case population	Eligible population	Complete case population
**Intercept**	277.3 (277.2 to 277.5)	277.9 (277.7 to 278.2)	3278 (3218 to 3339)&	3298 (3180 to 3420) &
**Sex**				
Female	0	0	1	1
Male	-0.56 (-0.65, -0.48)	-0.63 (-0.79, -0.47)	1.045 (1.044 to 1.046)	1.048 (1.046, 1.049)
**Birth rank**				
1¶	0	0	1	1
2	-0.39 (-0.49, -0.29)	-0.34 (-0.52, -0.16)	1.038 (1.037, 1.039)	1.039 (1.037, 1.041)
3	-0.37 (-0.52, -0.22)	-0.16 (-0.44, 0.11)	1.050 (1.048, 1.051)	1.054 (1.051, 1.057)
≥ 4	-0.24 (-0.50, 0.02)	0.24 (-0.25, 0.72)	1.058 (1.056, 1.061)	1.065 (1.059, 1.070)
**Age mother (yrs)‡**				
< 20 (per 5 yrs decr.)	-4.10 (-5.59, -2.61)	-	1.002 (0.987, 1.017)	-
≥ 20-30¶	0	0	1	1
≥ 30-40 (per 5 yrs)	-0.99 (-1.06, -0.91)	-0.93 (-1.07, -0.78)	1.000 (1.000, 1.001)	0.998 (0.997, 1.000)
≥ 40 (per 5 yrs)	-2.93 (-3.36, -2.50)	-3.46 (-4.29, -2.63)	0.998 (0.994, 1.003)	0.998 (0.990, 1.006)
**Civil status◊**				
Married	0	0	1	1
Not married	-0.01 (-0.13, 0.10)	0.15 (-0.08, 0.38)	0.990 (0.989, 0.991)	0.993 (0.99, 0.995)
**Nationality mother**				
Switzerland¶	0	0	1	1
S Europe	0.20 (-0.01, 0.40)	0.39 (00, 0.78)	0.994 (0.992, 0.996)	0.995 (0.991, 0.999)
W Europe	0.20 (0.02, 0.38)	-0.08 (-0.43, 0.26)	1.008 (1.006, 1.010)	1.007 (1.004, 1.011)
N Europe	0.37 (-0.07, 0.81)	0.30 (-0.57, 1.17)	1.025 (1.020, 1.029)	1.022 (1.013, 1.031)
E Europe	0.21 (0.04, 0.38)	0.33 (-0.01, 0.68)	1.013 (1.011, 1.014)	1.017 (1.014, 1.021)
Other	-0.32 (-0.49, -0.14)	-0.67 (-1.05, -0.30)	1.007 (1.005, 1.008)	1.012 (1.008, 1.016)
**Nationality father**				
Switzerland¶	0	0	1	1
S Europe	-0.46 (-0.64, -0.28)	-0.28 (-0.62, 0.06)	0.991 (0.990, 0.993)	0.993 (0.989, 0.996)
W Europe	0.07 (-0.11, 0.25)	0.30 (-0.04, 0.63)	1.008 (1.006, 1.009)	1.006 (1.003, 1.010)
N Europe	0.51 (0.08, 0.94)	-0.24 (-1.09, 0.62)	1.013 (1.009, 1.017)	1.011 (1.003, 1.020)
E Europe	-0.46 (-0.64, -0.28)	-0.01 (-0.38, 0.36)	1.009 (1.007, 1.010)	1.011 (1.008, 1.015)
Other	-0.02 (-0.22, 0.18)	0.48 (0.05, 0.90)	0.992 (0.991, 0.994)	0.992 (0.987, 0.996)
missing	-3.87 (-4.24, -3.50)	-	0.989 (0.985, 0.992)	-
**Education mother**				
Tertiary¶		0		1
Secondary		-0.55 (-0.74, -0.36)		0.996 (0.995, 0.998)
Compulsory		-0.90 (-1.22, -0.58)		0.993 (0.990, 0.996)
**Education father**				
Tertiary¶		0		1
Secondary		-0.16 (-0.35, 0.03)		0.996 (0.994, 0.998)
Compulsory		-0.25 (-0.58, 0.07)		0.997 (0.994, 1.000)
**Altitude (m)**				
500¶	0	0	1	1
per 500 m increase	0.07 (-0.09, 0.23)	0.03 (-0.24, 0.30)	0.989 (0.988, 0.991)	0.989 (0.987, 0.992)
**Urbanization**				
Urban¶	0	0	1	1
Peri-urban	-0.43 (-0.57, -0.28)	-0.59 (-0.82, -0.36)	1.001 (1.000, 1.002)	1.003 (1.000, 1.005)
Rural	-0.15 (-0.32, 0.02)	-0.29 (-0.55, -0.02)	1.000 (0.998, 1.001)	1.003 (1.001, 1.006)
**Language region**				
German¶	0	0	1	1
French	-0.62 (-0.77, -0.47)	-0.66 (-0.88, -0.44)	0.989 (0.987, 0.990)	0.988 (0.985, 0.990)
Italian	-0.94 (-1.26, -0.63)	-1.11 (-1.55, -0.68)	0.982 (0.980, 0.985)	0.983 (0.979, 0.987)
**Percent of spatial variance explained†**	31%	39%	87%	88%

*Birth weight was modelled on a log scale, which results in multiplicative effects. The model for birth weight was additionally adjusted for gestational age by a cubic spline function with knots at weeks 25, 30 and 35.

& In the model for BW, the intercept corresponds to an estimated mean birth weight (g) for a singleton girl born at gestational age 40 weeks as the first child (rank 1) in a German-speaking, urban region of elevation 500m, whose mother is 20-30 years old at birth and married, and both parents have Swiss nationality and tertiary education.

¶ Reference category

‡ Age modelled by a piece-wise linear function: constant at reference range ≥20-30, and separate slopes for age <20, ≥30-40, and ≥40.

◊ Married or in registered partnership / Not married: Single, widow, divorced or in dissolved partnership

† Percentage of regional variance explained by model predictors, i.e. percent reduction in variance of random effects (σ^2) when compared to model with no predictors (model 0).

For peer review only - http://bmjopen.bmj.com/site/about/guidelines.xhtml

Table 3. Percentage of spatial variation explained by each individual variable and explained in addition after adjusting for all other variables.

Spatial variation explained		Gestational age: Eligible population	Gestational age: Complete case population	Birth weight: Eligible population	Birth weight: Complete case population
**By single variables**					
**Pregnancy factors**	Gestational age	-	-	27%	34%
	Sex	0%	0%	1%	2%
	Birth rank	0%	1%	4%	0%
**Parental factors**	Maternal age	0%	1%	1%	1%
	Civil status	0%	0%	10%	5%
	Nationality mother	1%	3%	17%	17%
	Nationality father	3%	4%	25%	20%
	Nationality parents*	3%	5%	27%	23%
	Education mother	-	1%	-	0%
	Education father	-	1%	-	1%
	Education parents*	-	1%	-	1%
**Regional factors**	Altitude	0%	0%	10%	6%
	Urbanization	10%	12%	0%	0%
	Language region	23%	25%	62%	63%
**In addition to all other variables**					
**Pregnancy factors**	Gestational age	-	-	12%	12%
	Sex	0%	0%	0%	1%
	Birth rank	1%	0%	3%	1%
**Parental factors**	Maternal age	0%	1%	0%	0%
	Civil status	0%	0%	0%	0%
	Nationality mother	0%	0%	1%	2%
	Nationality father	1.5%	0%	1%	0%
	Nationality parents*	2.5%	0%	3%	4%
	Education mother	-	2%	-	0%
	Education father	-	0%	-	0%
	Education parents*	-	2%	-	1%
**Regional factors**	Altitude	0%	0%	9%	4%
	Urbanization	9%	10%	0%	1%
	Language region	17%	21%	22%	24%
	**Model 3 (full)**	**31%**	**39%**	**87%**	**88%**

- Data not available
* Nationality or educational attainment of both mother and father were entered into the model.

For peer review only

FIGURE LEGENDS

Figure 1. Maps of average gestational age (upper two panels) and birth weight (lower two panels) observed across 705 Swiss areas. Left: all eligible live births (n=315,177), right: complete case population (n=81,968). The orientation of the maps is standard, with North being up.

Figure 2. Maps of gestational age and birth weight from crude model (model 0) and multivariable linear mixed-effect models (models 1-3) with percent reduction in the regional variation, represented by random effects. Analyses based on complete case population (*N* = 81,968). The orientation of the maps is standard, with North being up.

FOOTNOTES

Contributors: ME and CEK conceived the study and obtained funding. VS, ME, MZ and CEK developed the analysis plan. VS did all statistical analyses and wrote the first draft of the paper, which was revised by ME and VS, taking into account the critical comments from MZ, MA, NL and CEK. All authors approved the final version of the report. ME supervised the study.

Funding: The Swiss National Cohort is funded by the Swiss National Science Foundation (SNSF) cohort grant No. 148415. The current analysis was funded by SNSF project grant No. 163452. ME was supported by special SNSF project funding (grant No. 174281).

Competing interests: None declared.

Patient consent: Not required.

Ethics approval: The SNC has been approved by the Ethics Committee of the Canton of Bern.

Provenance and peer review: Not commissioned; externally peer reviewed.

Data sharing statement: The data used in this study may be requested from the Federal Office of Statistics.

For peer review only

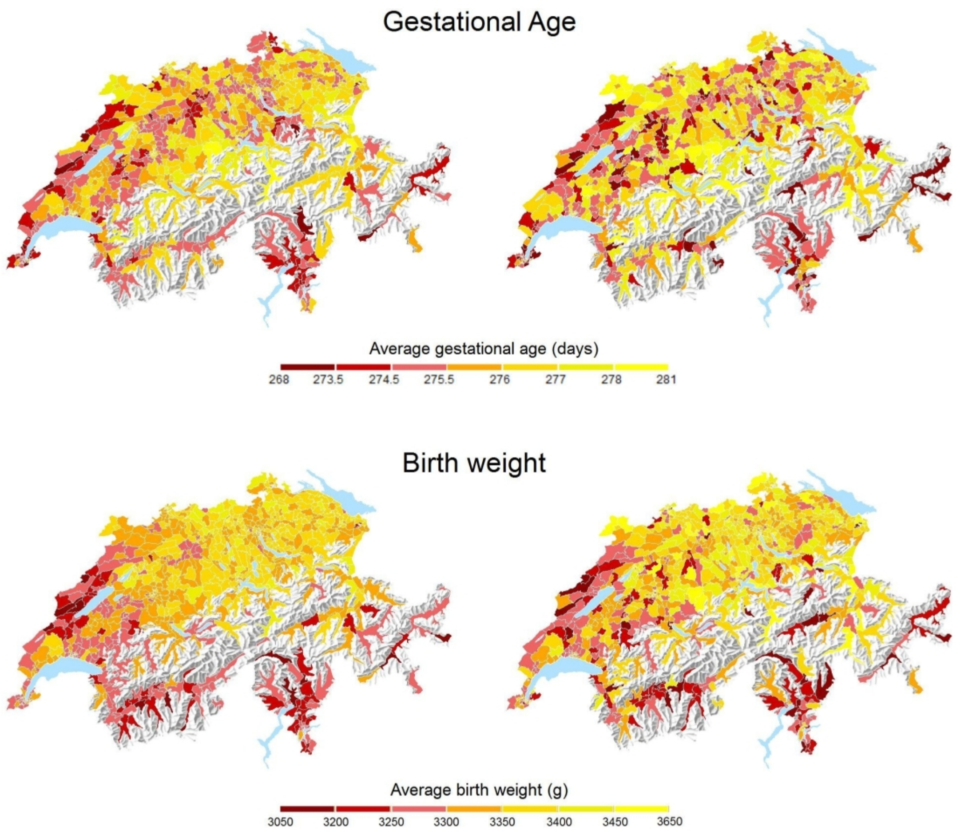

Figure 1. Maps of average gestational age (upper two panels) and birth weight (lower two panels) observed across 705 Swiss areas. Left: all eligible live births (n=315,177), right: complete case population (n=81,968). The orientation of the maps is standard, with North being up.

718x633mm (72 x 72 DPI)

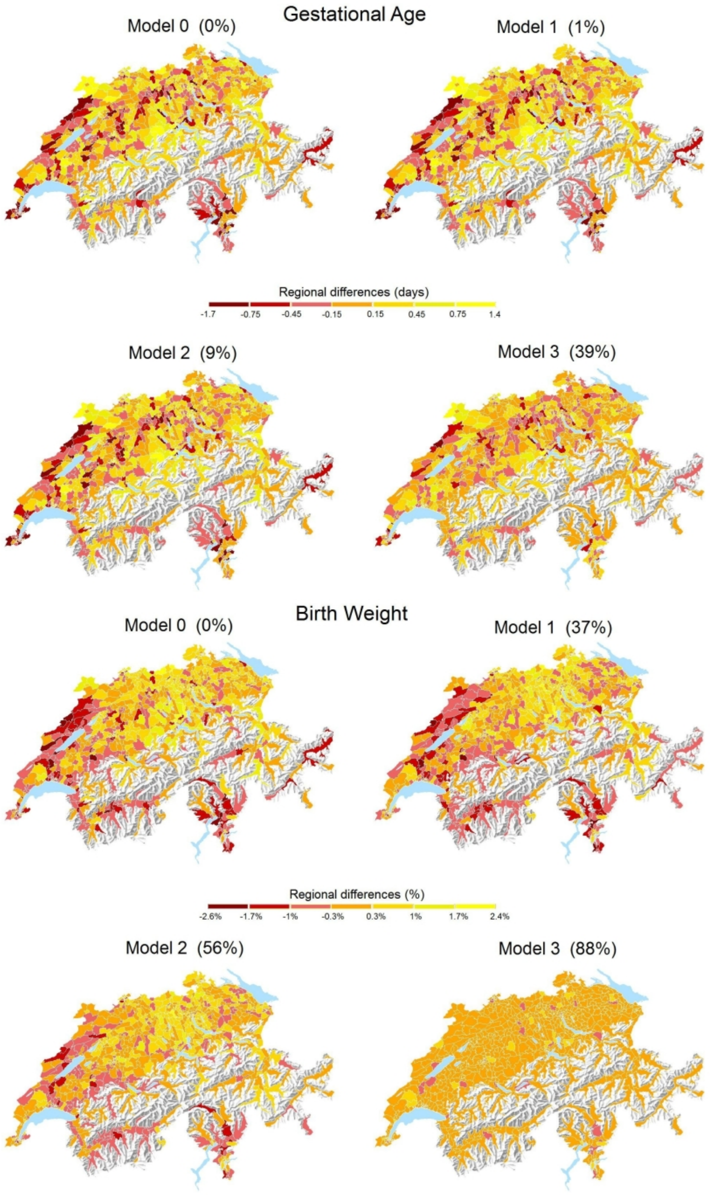

Figure 2. Maps of gestational age and birth weight from crude model (model 0) and multivariable linear mixed-effect models (models 1-3) with percent reduction in the regional variation, represented by random effects. Analyses based on complete case population (N = 81,968). The orientation of the maps is standard, with North being up.

718x1164mm (72 x 72 DPI)

SUPPLEMENTARY MATERIALS

Supplementary Table S1. Number of live births, mean gestational age and mean birth weight by maternal nationality in the eligible population (*N* = 315'177).

Nationality	N	Gestational age (days) Mean (SD)	Birth weight (g) Mean (SD)
**Switzerland**	194,570	276 (12)	3322 (511)
**Southern Europe**			
**Andorra**	1	279 (-)	3080 (-)
**Italy**	8337	275 (12)	3271 (496)
**Malta**	13	273 (8)	3188 (427)
**Portugal**	12,368	276 (12)	3235 (493)
**San Marino**	2	274 (1.4)	3485 (120)
**Spain**	2864	276 (12)	3263 (488)
**Western Europe**			
**Austria**	1555	275 (14)	3328 (528)
**Belgium**	583	276 (12)	3357 (482)
**Germany**	16,736	276 (13)	3369 (517)
**France**	6173	276 (12)	3294 (505)
**Lichtenstein**	100	275 (11)	3369 (488)
**Luxembourg**	55	276 (19)	3396 (636)
**Netherlands**	803	276 (12)	3377 (529)
**Northern Europe**			
**Denmark**	271	276 (13)	3383 (511)
**Estonia**	81	279 (7)	3601 (466)
**Finland**	312	276 (11)	3465 (523)
**Ireland**	212	276 (16)	3446 (548)
**Iceland**	31	272 (25)	3180 (775)
**Latvia**	187	279 (9)	3493 (434)
**Lithuania**	152	277 (13)	3450 (535)
**Norway**	110	275 (11)	3390 (525)
**Sweden**	571	276 (12)	3422 (470)
**UK**	1768	276 (13)	3397 (513)
**Eastern Europe**			
**Czech Republic**	623	275 (12)	3339 (499)
**Hungary**	913	275 (13)	3341 (512)
**Poland**	1778	276 (12)	3399 (497)
**Slovakia**	1068	276 (12)	3348 (509)
**Albania**	209	276 (12)	3406 (476)
**Bosnia & Herzegovina**	1952	276 (12)	3466 (492)
**Croatia**	1582	276 (12)	3448 (540)
**Kosovo**	10,278	276 (13)	3421 (530)
**Macedonia**	5842	276 (13)	3392 (514)
**Montenegro**	212	276 (10)	3416 (466)
**Serbia**	5195	276 (13)	3400 (536)
**Serbia & Montenegro**	10	277 (8)	3637 (250)
**Slovenia**	163	275 (15)	3366 (589)
**Cyprus**	15	278 (8)	3411 (525)
**Bulgaria**	406	273 (15)	3291 (559)
**Greece**	375	274 (13)	3317 (516)
**Romania**	971	274 (14)	3284 (537)
**Turkey**	4441	275 (13)	3347 (523)
**Belarus**	172	277 (13)	3385 (508)
**Moldova**	135	276 (10)	3496 (515)
**Russia**	1567	277 (12)	3427 (513)
**Ukraine**	855	277 (11)	3412 (473)
**Other (non-Europe)**			
6 most numerous: **Eritrea**	2600	279 (14)	3380 (528)
**Brazil**	2381	274 (12)	3312 (498)
**Sri Lanka**	1391	273 (14)	3158 (553)
**USA**	1291	276 (14)	3378 (532)
**China**	1293	276 (13)	3425 (541)
**Morocco**	1159	276 (14)	3378 (536)
. . .			
**Total**	315,177	276 (12)	3328 (515)

Supplementary Table S2. Comparison of results from fully adjusted model (model 3) accounting and not accounting for spatial autocorrelation. Based on complete-case population (*N* = 81,968).

	Accounting for spatial autocorrelation		Not accounting for spatial autocorrelation	
	**Gestational age (days) Absolute differences (95% CI)**	**Birth weight (g) * Relative differences (95% CI)**	**Gestational age (days) Absolute differences (95% CI)**	**Birth weight (g) * Relative differences (95% CI)**
Intercept	277.9 (277.6 to 278.1)	3293 (3163 to 3427)	277.9 (277.7 to 278.2)	3298 (3180 to 3420) [&]
**Sex**				
¶Female	0		0	1
Male	-0.62 (-0.78, -0.46)	1.048 (1.046, 1.049)	-0.63 (-0.79, -0.47)	1.048 (1.046, 1.049)
**Birth rank**				
1¶	0		0	1
2	-0.34 (-0.52, -0.17)	1.039 (1.037, 1.041)	-0.34 (-0.52, -0.16)	1.039 (1.037, 1.041)
3	-0.17 (-0.45, 0.10)	1.054 (1.051, 1.056)	-0.16 (-0.44, 0.11)	1.054 (1.051, 1.057)
≥ 4	0.23 (-0.26, 0.71)	1.065 (1.059, 1.070)	0.24 (-0.25, 0.72)	1.065 (1.059, 1.070)
**Maternal age (yrs)‡**				
≥ 20-30¶	0		0	1
≥ 30-40 yrs (per 5 yrs)	-0.92 (-1.06, -0.77)	0.998 (0.996, 1.000)	-0.93 (-1.07, -0.78)	0.998 (0.997, 1.000)
≥ 40 yrs (per 5 yrs)	-3.48 (-4.30, -2.66)	0.998 (0.990, 1.006)	-3.46 (-4.29, -2.63)	0.998 (0.990, 1.006)
**Civil status◊**				
Married¶	0		0	1
Not married	0.15 (-0.08, 0.38)	0.993 (0.990, 0.995)	0.15 (-0.08, 0.38)	0.993 (0.990, 0.995)
**Nationality mother**				
Switzerland¶	0		0	1
S Europe	0.38 (0.00, 0.77)	0.995 (0.992, 0.999)	0.39 (0.00, 0.78)	0.995 (0.991, 0.999)
W Europe	-0.08 (-0.42, 0.25)	1.007 (1.003, 1.010)	-0.08 (-0.43, 0.26)	1.007 (1.004, 1.011)
N Europe	0.30 (-0.56, 1.17)	1.022 (1.013, 1.031)	0.30 (-0.57, 1.17)	1.022 (1.013, 1.031)
E Europe	0.33 (-0.01, 0.67)	1.017 (1.013, 1.021)	0.33 (-0.01, 0.68)	1.017 (1.014, 1.021)
Other	-0.66 (-1.03, -0.29)	1.012 (1.008, 1.015)	-0.67 (-1.05, -0.30)	1.012 (1.008, 1.016)
**Nationality father**				
Switzerland¶	0		0	1
S Europe	-0.28 (-0.61, 0.06)	0.992 (0.989, 0.996)	-0.28 (-0.62, 0.06)	0.993 (0.989, 0.996)
W Europe	0.30 (-0.03, 0.64)	1.006 (1.002, 1.009)	0.30 (-0.04, 0.63)	1.006 (1.003, 1.010)
N Europe	-0.21 (-1.06, 0.63)	1.011 (1.002, 1.019)	-0.24 (-1.09, 0.62)	1.011 (1.003, 1.020)
E Europe	-0.02 (-0.38, 0.35)	1.011 (1.007, 1.015)	-0.01 (-0.38, 0.36)	1.011 (1.008, 1.015)
Other	0.49 (0.06, 0.91)	0.991 (0.987, 0.996)	0.48 (0.05, 0.90)	0.992 (0.987, 0.996)
**Education mother**				
Tertiary¶			0	1
Secondary	-0.56 (-0.75, -0.37)	0.997 (0.995, 0.999)	-0.55 (-0.74, -0.36)	0.996 (0.995, 0.998)
Compulsory	-0.92 (-1.23, -0.60)	0.993 (0.990, 0.997)	-0.90 (-1.22, -0.58)	0.993 (0.990, 0.996)
**Education father**				
Tertiary¶			0	1
Secondary	-0.16 (-0.35, 0.03)	0.997 (0.995, 0.999)	-0.16 (-0.35, 0.03)	0.996 (0.994, 0.998)
Compulsory	-0.25 (-0.57, 0.07)	0.997 (0.994, 1.001)	-0.25 (-0.58, 0.07)	0.997 (0.994, 1.000)
**Altitude (m)**				
500¶	0		0	1
per 500 m increase	-0.05 (-0.33, 0.24)	0.991 (0.987, 0.994)	0.03 (-0.24, 0.30)	0.989 (0.987, 0.992)
**Urbanization**				
Urban¶	0		0	1
Peri-urban	-0.54 (-0.75, -0.33)	1.001 (0.998, 1.004)	-0.59 (-0.82, -0.36)	1.003 (1.000, 1.005)
Rural	-0.25 (-0.50, 0.00)	1.003 (0.999, 1.006)	-0.29 (-0.55, -0.02)	1.003 (1.001, 1.006)
**Language region**				
German¶	0		0	1
French	-0.33 (-0.75, 0.09)	0.991 (0.983, 0.998)	-0.66 (-0.88, -0.44)	0.988 (0.985, 0.990)
Italian	-1.10 (-1.50, -0.70)	0.984 (0.978, 0.989)	-1.11 (-1.55, -0.68)	0.983 (0.979, 0.987)

*Birth weight was modelled on a log scale, which results in multiplicative effects. The model for birth weight was additionally adjusted for gestational age by a cubic spline function with knots at weeks 25, 30 and 35.

& In the model for BW, the intercept corresponds to an estimated mean birth weight (g) for a singleton girl born at gestational age 40 weeks as the first child (rank 1) in a German-speaking, urban region of elevation 500 m, whose mother is 20-30 years old at birth and married, and both parents have Swiss nationality and tertiary education.

¶ Reference category

‡ Age modelled by a piece-wise linear function: constant at reference range ≥20-30, and separate slopes for age <20, ≥30-40, and ≥40. For ages ≥40, the total estimated effect is hence addition of 10-year effect in age group ≥30-40 plus the corresponding effect in age-group ≥40.

† Percentage of regional variance explained by model predictors, i.e. percent reduction in variance of random effects (σ^2) when compared to model with no predictors (model 0).

Supplementary Table S3. Comparison of results from model (model 3) for birth weight, adjusted and not adjusted for gestational age.

	Birth weight - Model 3 without Gestational Age		Birth weight - Model 3 with Gestational Age*	
	Relative differences (95% CI)		Relative differences (95% CI)	
	Eligible population	Complete case population	Eligible population	Complete case population
**Intercept**	3188 (3181 to 3195)	3209 (3196 to 3222)	3278 (3218 to 3339)[&]	3298 (3180 to 3420)[&]
**Sex**				
¶Female	1	1	1	1
Male	1.04 (1.038, 1.041)	1.041 (1.039, 1.044)	1.045 (1.044 to 1.046)	1.048 (1.046, 1.049)
**Rank**				
1¶	1	1	1	1
2	1.039 (1.038, 1.041)	1.041 (1.038, 1.044)	1.038 (1.037, 1.039)	1.039 (1.037, 1.041)
3	1.052 (1.049, 1.054)	1.058 (1.054, 1.063)	1.050 (1.048, 1.051)	1.054 (1.051, 1.057)
≥ 4	1.060 (1.056, 1.064)	1.071 (1.063, 1.079)	1.058 (1.056, 1.061)	1.065 (1.059, 1.070)
**Age mother (yrs)‡**				
< 20yrs (per 5 yrs decr.)	0.956 (0.935, 0.978)	-	1.002 (0.987, 1.017)	-
≥ 20-30 yrs¶	1	1	1	1
≥ 30-40 yrs (per 5 yrs)	0.991 (0.990, 0.992)	0.990 (0.988, 0.993)	1.000 (1.000, 1.001)	0.998 (0.997, 1.000)
≥ 40 yrs (per 5 yrs)	0.973 (0.967, 0.979)	0.967 (0.955, 0.979)	0.998 (0.994, 1.003)	0.998 (0.990, 1.006)
**Civil status**[◊]				
Married¶	1	1	1	1
Not married	0.989 (0.987, 0.990)	0.992 (0.989, 0.996)	0.990 (0.989, 0.991)	0.993 (0.99, 0.995)
**Nationality mother**				
Switzerland¶	1	1	1	1
S Europe	0.996 (0.993, 0.999)	0.999 (0.993, 1.005)	0.994 (0.992, 0.996)	0.995 (0.991, 0.999)
W Europe	1.010 (1.007, 1.013)	1.006 (1.001, 1.011)	1.008 (1.006, 1.010)	1.007 (1.004, 1.011)
N Europe	1.027 (1.020, 1.034)	1.024 (1.010, 1.037)	1.025 (1.020, 1.029)	1.022 (1.013, 1.031)
E Europe	1.014 (1.011, 1.016)	1.020 (1.015, 1.026)	1.013 (1.011, 1.014)	1.017 (1.014, 1.021)
Other	1.003 (1.000, 1.006)	1.005 (0.999, 1.010)	1.007 (1.005, 1.008)	1.012 (1.008, 1.016)
**Nationality father**				
Switzerland¶	1	1	1	1
S Europe	0.988 (0.985, 0.991)	0.991 (0.986, 0.996)	0.991 (0.990, 0.993)	0.993 (0.989, 0.996)
W Europe	1.008 (1.005, 1.011)	1.009 (1.004, 1.014)	1.008 (1.006, 1.009)	1.006 (1.003, 1.010)
N Europe	1.017 (1.010, 1.024)	1.007 (0.994, 1.019)	1.013 (1.009, 1.017)	1.011 (1.003, 1.020)
E Europe	1.003 (1.000, 1.006)	1.010 (1.004, 1.015)	1.009 (1.007, 1.010)	1.011 (1.008, 1.015)
Other	0.990 (0.987, 0.993)	0.997 (0.990, 1.003)	0.992 (0.991, 0.994)	0.992 (0.987, 0.996)
missing	0.933 (0.928, 0.938)	-	0.989 (0.985, 0.992)	-
**Education mother**				
Tertiary¶	-	1	-	1
Secondary	-	0.992 (0.989, 0.995)	-	0.996 (0.995, 0.998)
Compulsory	-	0.984 (0.979, 0.989)	-	0.993 (0.990, 0.996)
**Education father**				
Tertiary¶	-	1	-	1
Secondary	-	0.995 (0.992, 0.997)	-	0.996 (0.994, 0.998)
Compulsory	-	0.995 (0.990, 1.000)	-	0.997 (0.994, 1.000)
**Altitude (m)**				
500 m¶	1	1	1	1
per 500 m increase	0.990 (0.988, 0.992)	0.989 (0.985, 0.993)	0.989 (0.988, 0.991)	0.989 (0.987, 0.992)
**Urbanization**				
Urban¶	1	1	1	1
Peri-urban	0.998 (0.996, 1.000)	0.998 (0.995, 1.002)	1.001 (1.000, 1.002)	1.003 (1.000, 1.005)
Rural	0.999 (0.996, 1.001)	1.001 (0.997, 1.005)	1.000 (0.998, 1.001)	1.003 (1.001, 1.006)
**Language region**				
[R]German¶	1	1	1	1
French	0.985 (0.983, 0.987)	0.983 (0.980, 0.986)	0.989 (0.987, 0.990)	0.988 (0.985, 0.990)
Italian	0.977 (0.973, 0.981)	0.976 (0.970, 0.982)	0.982 (0.980, 0.985)	0.983 (0.979, 0.987)
**% variation explained**				
Model 3	77%	76%	87%	88%
Model 2	25%	27%	52%	56%
Model 1	2%	5%	31%	37%

*Birth weight was modelled on a log scale, which results in multiplicative effects.
¶ Reference category
‡ Age modelled by a piece-wise linear function: constant at reference range ≥20-30, and separate slopes for age <20, ≥30-40, and ≥40. For ages ≥40, the total estimated effect is hence addition of 10-year effect in age group ≥30-40 plus the corresponding effect in age-group ≥40.
† Percentage of regional variance explained by model predictors, i.e. percent reduction in variance of random effects (σ^2) when compared to model with no predictors (model 0).

For peer review only

Supplementary Table S4. Comparison of results from fully adjusted model without and with additionally including Swiss neighbourhood index of socio-economic position (SEP). Based on eligible population (*N* = 315,177).

	**Model 3 without SEP**		**Model 3 with SEP**	
	Gestational age (days) Absolute differences (95% CI)	Birth weight (g) * Relative differences (95% CI)	Gestational age (days) Absolute differences (95% CI)	Birth weight (g) * Relative differences (95% CI)
**Intercept**	277.3 (277.2 to 277.5)	3278 (3218 to 3339)[&]	277.3 (277.2 to 277.5)	3278 (3218 to 3339)[&]
**Sex**				
¶Female	0	1	0	1
Male	-0.56 (-0.65, -0.48)	1.045 (1.044 to 1.046)	-0.56 (-0.65, -0.48)	1.045 (1.044 to 1.046)
**Birth rank**				
1¶	0	1	0	1
2	-0.39 (-0.49, -0.29)	1.038 (1.037, 1.039)	-0.39 (-0.49, -0.29)	1.038 (1.037, 1.039)
3	-0.37 (-0.52, -0.22)	1.050 (1.048, 1.051)	-0.37 (-0.52, -0.22)	1.050 (1.048, 1.051)
≥ 4	-0.24 (-0.50, 0.02)	1.058 (1.056, 1.061)	-0.24 (-0.49, 0.02)	1.058 (1.056, 1.061)
**Age mother (yrs)‡**				
< 20 (per 5 yrs decr.)	-4.10 (-5.59, -2.61)	1.002 (0.987, 1.017)	-4.10 (-5.59, -2.61)	1.002 (0.987, 1.017)
20 - <30¶	0	1	0	1
≥ 30-40 (per 5 yrs)	-0.99 (-1.06, -0.91)	1.000 (1.000, 1.001)	-0.99 (-1.07, -0.92)	1.000 (1.000, 1.001)
≥ 40 (per 5 yrs)	-2.93 (-3.36, -2.50)	0.998 (0.994, 1.003)	-2.92 (-3.35, -2.50)	0.998 (0.994, 1.003)
**Civil status◊**				
Married	0	1	0	1
Not married	-0.01 (-0.13, 0.10)	0.990 (0.989, 0.991)	-0.01 (-0.13, 0.10)	0.990 (0.989, 0.991)
**Nationality mother**				
Switzerland¶	0	1	0	1
S Europe	0.20 (-0.01, 0.40)	0.994 (0.992, 0.996)	0.20 (0.00, 0.41)	0.994 (0.992, 0.996)
W Europe	0.20 (0.02, 0.38)	1.008 (1.006, 1.010)	0.20 (0.02, 0.37)	1.008 (1.006, 1.010)
N Europe	0.37 (-0.07, 0.81)	1.025 (1.020, 1.029)	0.36 (-0.08, 0.80)	1.025 (1.020, 1.029)
E Europe	0.21 (0.04, 0.38)	1.013 (1.011, 1.014)	0.22 (0.05, 0.39)	1.013 (1.011, 1.015)
Other	-0.32 (-0.49, -0.14)	1.007 (1.005, 1.008)	-0.31 (-0.48, -0.14)	1.007 (1.005, 1.008)
**Nationality father**				
Switzerland¶	0	1	0	1
S Europe	-0.46 (-0.64, -0.28)	0.991 (0.990, 0.993)	-0.45 (-0.63, -0.27)	0.992 (0.99, 0.993)
W Europe	0.07 (-0.11, 0.25)	1.008 (1.006, 1.009)	0.07 (-0.11, 0.25)	1.008 (1.006, 1.009)
N Europe	0.51 (0.08, 0.94)	1.013 (1.009, 1.017)	0.50 (0.07, 0.93)	1.013 (1.009, 1.017)
E Europe	-0.46 (-0.64, -0.28)	1.009 (1.007, 1.010)	-0.45 (-0.63, -0.27)	1.009 (1.007, 1.011)
Other	-0.02 (-0.22, 0.18)	0.992 (0.991, 0.994)	-0.01 (-0.21, 0.19)	0.993 (0.991, 0.995)
missing	-3.87 (-4.24, -3.50)	0.989 (0.985, 0.992)	-3.86 (-4.23, -3.49)	0.989 (0.985, 0.993)
**SEP index**				
1st quintile	-	-	-0.08 (-0.25, 0.08)	0.997 (0.996, 0.999)
2nd quintile	-	-	-0.09 (-0.24, 0.06)	0.998 (0.997, 1.000)
3rd quintile	-	-	-0.02 (-0.17, 0.13)	0.998 (0.997, 0.999)
4th quintile	-	-	0.02 (-0.12, 0.17)	1.000 (0.999, 1.002)
5th quintile¶	-	-	0	1
**Altitude (m)**				
500¶	0	1	0	1
per 500 m increase	0.07 (-0.09, 0.23)	0.989 (0.988, 0.991)	0.08 (-0.08, 0.23)	0.989 (0.988, 0.991)
**Urbanization**				
Urban¶	0	1	0	1
Peri-urban	-0.43 (-0.57, -0.28)	1.001 (1.000, 1.002)	-0.43 (-0.57, -0.28)	1.001 (1.000, 1.002)
Rural	-0.15 (-0.32, 0.02)	1.000 (0.998, 1.001)	-0.13 (-0.30, 0.04)	1.001 (0.999, 1.002)
**Language region**				
German¶	0	1	0	1
French	-0.62 (-0.77, -0.47)	0.989 (0.987, 0.990)	-0.61 (-0.76, -0.46)	0.989 (0.987, 0.990)
Italian	-0.94 (-1.26, -0.63)	0.982 (0.980, 0.985)	-0.93 (-1.24, -0.61)	0.982 (0.980, 0.985)
**% variation explained**				
Full model	31%	87%	31%	88%
Model without Lang. region	14%	66%	15%	68%

*Birth weight was modelled on a log scale, which results in multiplicative effects. The model for birth weight was additionally adjusted for gestational age by a cubic spline function with knots at weeks 25, 30 and 35.

& In the model for BW, the intercept corresponds to an estimated mean birth weight (g) for a singleton girl born at gestational age 40 weeks as the first child (rank 1) in a German-speaking, urban region of elevation 500m, whose mother is 20-30 years old at birth and married, and both parents have Swiss nationality and tertiary education.

¶ Reference category

‡ Age modelled by a piece-wise linear function: constant at reference range ≥20-30, and separate slopes for age <20, ≥30-40, and ≥40.

◊ Married or in registered partnership / Not married: Single, widow, divorced or in dissolved partnership

† Percentage of regional variance explained by model predictors, i.e. percent reduction in variance of random effects (σ^2) when compared to model with no predictors (model 0).

For peer review only

Supplementary Figure S1. Selection of eligible and complete case study populations among live births in Switzerland 2011 to 2014.

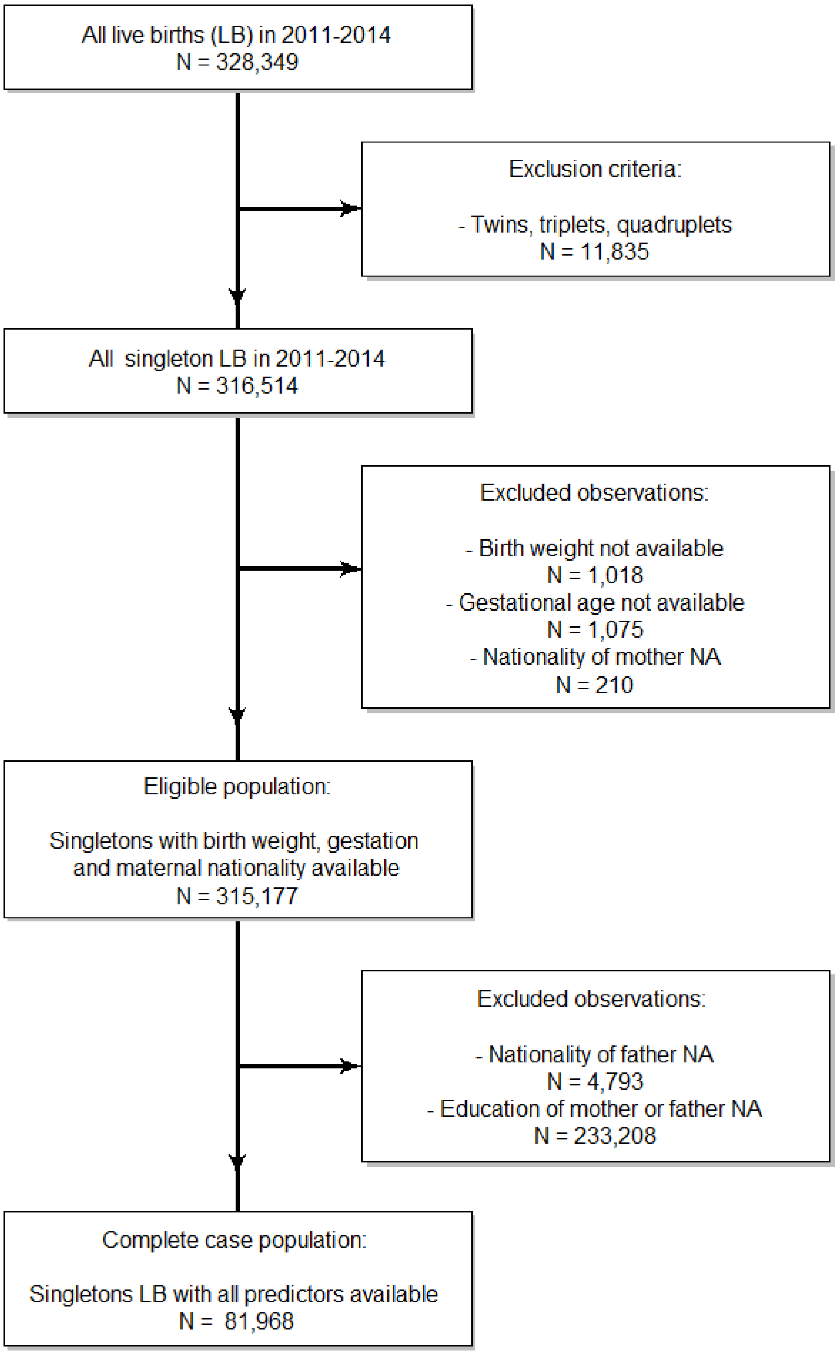

Supplementary Figure S2. Relationship between birth weight and gestational age at birth modeled by a cubic spline function. Separate fitted curves are shown for newborn girls and boys, with all other predictors corresponding to the reference categories shown in Table 2.

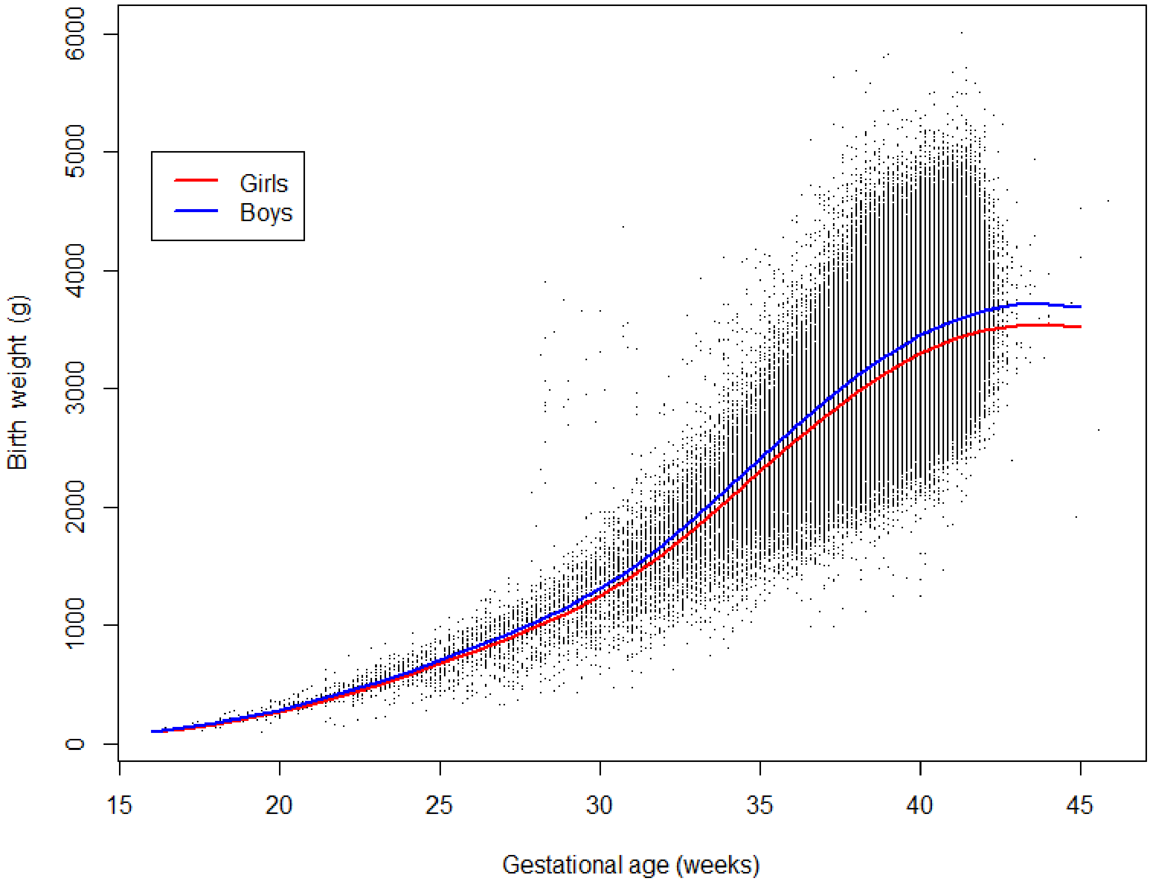

Supplementary Figure S3. Relationship between mean gestational age and proportion of preterm live births (<37 weeks) among eligible live births across 705 regions (upper panel) and between mean birth weight and proportion of low birth weight births (<2500g) (lower panel). Results from linear regression weighted by the number of live births in each region. Prediction intervals displayed for an average-size region (n=447). GA = gestational age; BW= birth weight; 276 days correspond to 39$^{3/7}$ weeks.

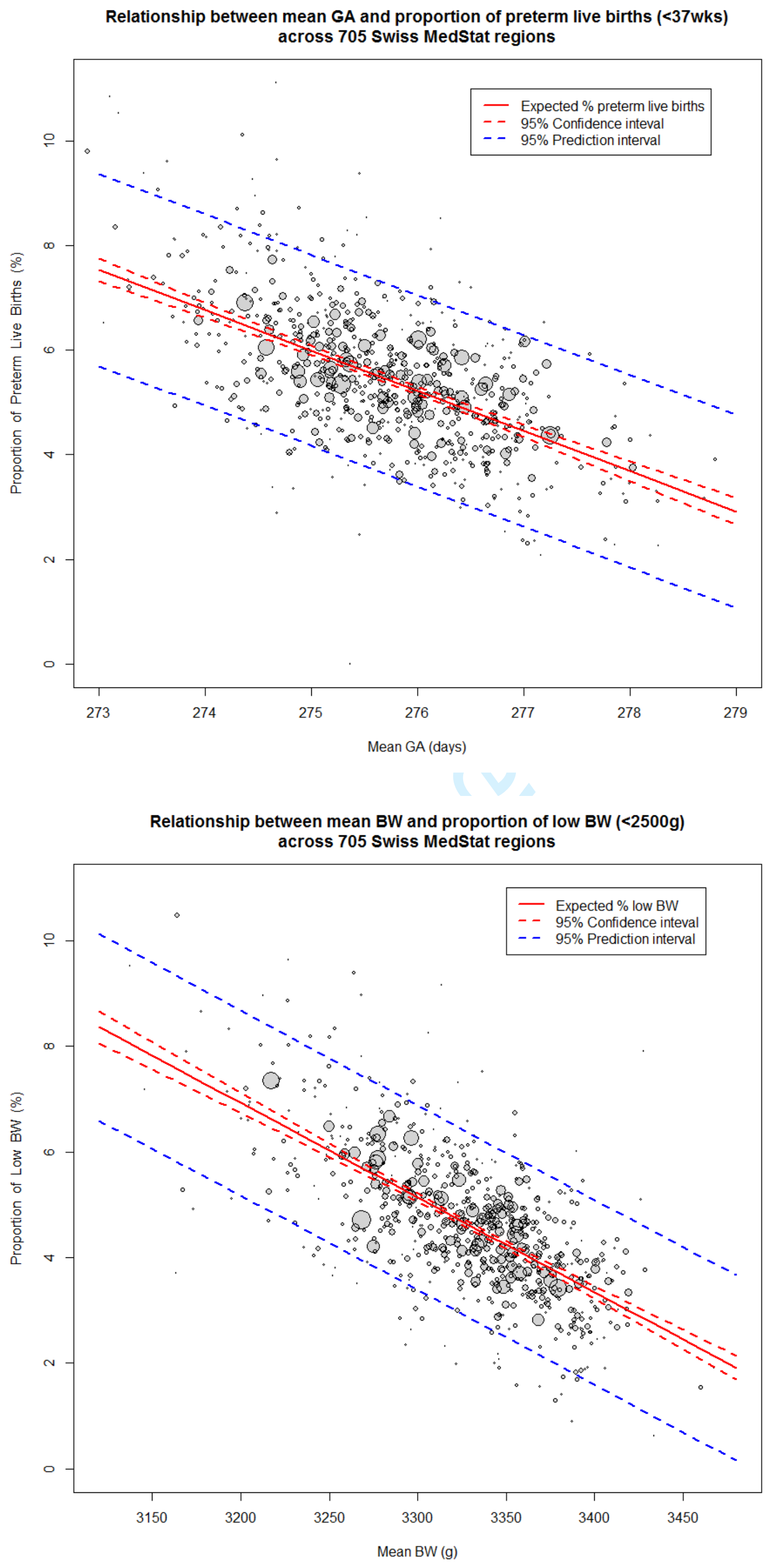

STROBE Statement—checklist of items that should be included in reports of observational studies

	Item No	Recommendation	Page No
**Title and abstract**	1	(*a*) Indicate the study's design with a commonly used term in the title or the abstract	1
		(*b*) Provide in the abstract an informative and balanced summary of what was done and what was found	2
**Introduction**			
Background/rationale	2	Explain the scientific background and rationale for the investigation being reported	4
Objectives	3	State specific objectives, including any prespecified hypotheses	4
**Methods**			
Study design	4	Present key elements of study design early in the paper	5
Setting	5	Describe the setting, locations, and relevant dates, including periods of recruitment, exposure, follow-up, and data collection	6
Participants	6	(*a*) *Cohort study*—Give the eligibility criteria, and the sources and methods of selection of participants. Describe methods of follow-up<br>*Case-control study*—Give the eligibility criteria, and the sources and methods of case ascertainment and control selection. Give the rationale for the choice of cases and controls<br>*Cross-sectional study*—Give the eligibility criteria, and the sources and methods of selection of participants	6
		(*b*) *Cohort study*—For matched studies, give matching criteria and number of exposed and unexposed<br>*Case-control study*—For matched studies, give matching criteria and the number of controls per case	
Variables	7	Clearly define all outcomes, exposures, predictors, potential confounders, and effect modifiers. Give diagnostic criteria, if applicable	5
Data sources/ measurement	8*	For each variable of interest, give sources of data and details of methods of assessment (measurement). Describe comparability of assessment methods if there is more than one group	5
Bias	9	Describe any efforts to address potential sources of bias	7
Study size	10	Explain how the study size was arrived at	8
Quantitative variables	11	Explain how quantitative variables were handled in the analyses. If applicable, describe which groupings were chosen and why	6
Statistical methods	12	(*a*) Describe all statistical methods, including those used to control for confounding	6
		(*b*) Describe any methods used to examine subgroups and interactions	na
		(*c*) Explain how missing data were addressed	8
		(*d*) *Cohort study*—If applicable, explain how loss to follow-up was addressed<br>*Case-control study*—If applicable, explain how matching of cases and controls was addressed<br>*Cross-sectional study*—If applicable, describe analytical methods taking account of sampling strategy	na
		(*e*) Describe any sensitivity analyses	7

Continued on next page

For peer review only - http://bmjopen.bmj.com/site/about/guidelines.xhtml

**Results**			
Participants	13*	(a) Report numbers of individuals at each stage of study—eg numbers potentially eligible, examined for eligibility, confirmed eligible, included in the study, completing follow-up, and analysed	8
		(b) Give reasons for non-participation at each stage	8
		(c) Consider use of a flow diagram	Figure S1
Descriptive data	14*	(a) Give characteristics of study participants (eg demographic, clinical, social) and information on exposures and potential confounders	Table 1, Table S1
		(b) Indicate number of participants with missing data for each variable of interest	Table 1
		(c) *Cohort study*—Summarise follow-up time (eg, average and total amount)	
Outcome data	15*	*Cohort study*—Report numbers of outcome events or summary measures over time	
		Case-control study—Report numbers in each exposure category, or summary measures of exposure	
		Cross-sectional study—Report numbers of outcome events or summary measures	Table 1
Main results	16	(*a*) Give unadjusted estimates and, if applicable, confounder-adjusted estimates and their precision (eg, 95% confidence interval). Make clear which confounders were adjusted for and why they were included	Table 2, Table S3
		(*b*) Report category boundaries when continuous variables were categorized	All tables
		(*c*) If relevant, consider translating estimates of relative risk into absolute risk for a meaningful time period	na
Other analyses	17	Report other analyses done—eg analyses of subgroups and interactions, and sensitivity analyses	Table S2, S3
**Discussion**			
Key results	18	Summarise key results with reference to study objectives	11
Limitations	19	Discuss limitations of the study, taking into account sources of potential bias or imprecision. Discuss both direction and magnitude of any potential bias	12
Interpretation	20	Give a cautious overall interpretation of results considering objectives, limitations, multiplicity of analyses, results from similar studies, and other relevant evidence	12
Generalisability	21	Discuss the generalisability (external validity) of the study results	12
**Other information**			
Funding	22	Give the source of funding and the role of the funders for the present study and, if applicable, for the original study on which the present article is based	29

*Give information separately for cases and controls in case-control studies and, if applicable, for exposed and unexposed groups in cohort and cross-sectional studies.

Note: An Explanation and Elaboration article discusses each checklist item and gives methodological background and published examples of transparent reporting. The STROBE checklist is best used in conjunction with this article (freely available on the Web sites of PLoS Medicine at http://www.plosmedicine.org/, Annals of Internal Medicine at

For peer review only

http://www.annals.org/, and Epidemiology at http://www.epidem.com/). Information on the STROBE Initiative is available at www.strobe-statement.org.

For peer review only

For peer review only - http://bmjopen.bmj.com/site/about/guidelines.xhtml

BMJ Open

Spatial epidemiology of gestational age and birth weight in Switzerland: Census-based linkage study

Journal:	*BMJ Open*
Manuscript ID	bmjopen-2018-027834.R2
Article Type:	Original research
Date Submitted by the Author:	09-Oct-2019
Complete List of Authors:	Skrivankova, Veronika; Universitat Bern Institut fur Sozial- und Praventivmedizin,<br>Zwahlen , Marcel; Universitat Bern Institut fur Sozial- und Praventivmedizin<br>Adams, Mark; University of Zurich, Department of Neonatology<br>Low, Nicola; University of Bern, Institute of Social and Preventive Medicine<br>Kuehni, Claudia; Universitat Bern Institut fur Sozial- und Praventivmedizin<br>Egger, Matthias; University of Bern, Institute of Social and Preventive Medicine
**Primary Subject Heading**:	Public health
Secondary Subject Heading:	Reproductive medicine, Obstetrics and gynaecology
Keywords:	birth weight, gestational age, spatial epidemiology, Switzerland, socio-economic position, education

SCHOLARONE™
Manuscripts

Spatial epidemiology of gestational age and birth weight in Switzerland: Census-based linkage study

Veronika Skrivankova[1], Marcel Zwahlen[1], Mark Adams[2], Nicola Low[1], Claudia E Kuehni[1,3], Matthias Egger[1]

1) Institute of Social and Preventive Medicine (ISPM), University of Bern, Bern, Switzerland

2) Department of Neonatology, University Hospital Zurich, University of Zurich, Zurich, Switzerland

3) Pediatric Respiratory Medicine, Inselspital, Bern University Hospital, University of Bern, Switzerland

Correspondence to:

Professor Matthias Egger MD

Institute of Social and Preventive Medicine (ISPM), University of Bern

Mittelstrasse 43, CH-3012 Bern, Switzerland

matthias.egger@ispm.unibe.ch

Word counts: Abstract 285 words, strengths and limitations of the study 91 words, main text 3484 words, 43 references, 3 tables, 2 figures, supplementary materials with 4 tables and 3 figures.

For peer review only - http://bmjopen.bmj.com/site/about/guidelines.xhtml

ABSTRACT

Background: Gestational age and birth weight are strong predictors of infant morbidity and mortality. Understanding spatial variation can inform policies to reduce health inequalities. We examined small-area variation in gestational age and birth weight in Switzerland.

Methods: All singleton live births recorded in the Swiss Live Birth Register 2011 to 2014 were eligible. We deterministically linked the Live Birth Register with census and survey data to create datasets including neonatal and pregnancy-related variables, parental characteristics and geographical variables. We produced maps of 705 areas and fitted linear mixed-effect models to assess to what extent spatial variation was explained by these variables.

Results: We analysed all 315,177 eligible live births. Area-level averages of gestational age varied between 272-279 days, and between 3138-3467g for birth weight. The fully adjusted models explained 31% and 87% of spatial variation of gestational age and birth weight, respectively. Language region accounted for most of the explained variation (23% in gestational age and 62% in birthweight), with shorter gestational age (-0.6 days and -0.9 days) and lower birth weight (-1.1% and -1.8%) in French- and Italian-speaking areas, respectively, compared to German-speaking areas. Other variables explaining variation were, for gestational age, the level of urbanization (10%) and parental nationality (3%). For birth weight, they were gestational age (27%), parental nationality (27%), civil status (10%) and altitude (10%). In a random sample of 81,968 live births with data on parental education, levels of education were only weakly associated with gestational age (-0.9 days for compulsory vs. tertiary maternal education) or birth weight (-0.7% for compulsory vs. tertiary maternal education).

Conclusions: In Switzerland, small area variation in birth weight is largely explained, and variation in gestational age partially explained, by geocultural, socio-demographic and pregnancy factors.

For peer review only - http://bmjopen.bmj.com/site/about/guidelines.xhtml

For peer review only

Strengths and limitations of this study

- This study was based on a large sample with national coverage and routinely collected data on neonatal and pregnancy-related predictors of gestational age and birth weight.
- Precise location data allowed for detailed geographical maps of spatial distribution and assessment of spatial variation in the two birth outcomes.
- No data were available on the mode of delivery, health-related behaviours such as maternal smoking, or gestational diabetes.
- Parental nationality served as crude proxy for parental height and weight, and language region as a proxy for a range of cultural, social and behavioural factors.

For peer review only

For peer review only - http://bmjopen.bmj.com/site/about/guidelines.xhtml

INTRODUCTION

Gestational age and birth weight are important indicators of prenatal development and predictors of infant morbidity, mortality and long-term health [1–4]. An understanding of geographic differences and their determinants can help to develop policies that reduce health inequalities across population groups and regions [1–4]. Many genetic, physiological, pregnancy-related, socio-economic, lifestyle and environmental factors have been reported to influence gestational age and birth weight [5–8]. Some of these factors tend to cluster in space and regional differences in health outcomes may hence be partially explained by the spatial distribution of their predictors. Importantly, both individual-level factors and the social and environmental characteristics of communities and neighbourhoods may contribute to regional differences [9,10].

Variation across small areas in pregnancy outcomes have not been studied widely. In Scotland, small area crime rates were associated with lower birth weight and with the risk of both small for gestational age babies and preterm birth [11]. A study at county level in Georgia and South Carolina in the United States showed that the proportion of African Americans was associated with low birth weight, whereas higher income was associated with higher birth weight [12]. Similarly, neighbourhood racial composition contributed to variation in low birth weight in New York State [13]. Other small-area analyses have examined associations between birth outcomes and air pollution [14,15]. To our knowledge, few small-area analyses have considered gestational age.

In Switzerland, studies of pregnancy outcomes have focused on specific groups such as migrants or HIV-infected women [16,17], but have not examined geographic variations. The Federal Office of Statistics publishes routine statistics from the Live Birth Register, which does not include geographic information [18]. The objectives of this study were to conduct a nationwide analysis of spatial variation in gestational age and birth weight, and to assess how much small-area variation was explained by available data about neonatal and pregnancy-related variables, parental characteristics and geographical variables.

For peer review only

METHODS

Data sources

We used deterministic methods to link three data sources using encrypted national identification numbers: the Live Birth Register, the Swiss National Cohort and the Structural Surveys. Registration of live births is compulsory by law in Switzerland coverage is near 100%. The Swiss National Cohort (SNC) is a long-term, national study of mortality in Switzerland [19,20], linking census and mortality records. The 1990 and 2000 censuses were the last house-to-house censuses with coverage of the entire Swiss population. From 2010 onwards, the national census was replaced by a national population register and annual postal survey of the resident population, known as Structural Surveys [21]. Each structural Survey includes a random sample of around 300,000 people aged 15 years or older; for example, in 2010, it included 317,221 persons [21]. The reference is the entire Swiss resident population and the reference day 31 December.

Variables and definitions

We defined three sets of variables. The first set, neonatal and pregnancy-related variables come from the Live Birth Register; date of birth, birth weight, gestational age, sex and birth rank. Birth weight is measured after initial mother-child bonding, usually by the midwife using a calibrated hospital scale. Gestational age is based on the last menstrual period, with or without additional information from ultrasound scans. Birth rank was determined from the list of all live births by the same mother recorded in the Live Birth Register, and is hence restricted to the births that occurred in Switzerland. It was classified as 1, 2, 3 and ≥4 live births, including the current birth. The second set includes parental variables. The Structural Surveys provide information about the highest level of completed maternal and paternal education, classified as 'tertiary', 'secondary', or 'compulsory or less'. The Swiss National Cohort provides data about parental nationality categorised as 'Swiss', 'Southern Europe', 'Western Europe', 'Northern Europe', 'Eastern Europe', 'Other' (non-European), or missing (supplementary Table S1 gives the full list of countries). The third set, geographical variables comes from the Swiss National Cohort. Each live birth was assigned an altitude and one of 705 statistical areas [22], based on the geocode of place of residence of the mother at the time of birth. Language regions are 'German', 'French' and 'Italian', and the level of urbanisation was defined using standard definitions of 'urban', 'peri-urban' and 'rural'.

For peer review only - http://bmjopen.bmj.com/site/about/guidelines.xhtml

Study populations and outcomes

All singleton live births recorded in the Live Birth Register from 1 January 2011 to 31 December 2014 were eligible. Gestational age at birth and birth weight were the outcomes of interest. For each outcome, two datasets were analysed: the first, larger dataset consisted of all eligible births with complete data on gestational age, birth weight and nationality of the mother. The second was the complete case population containing eligible live births with available data on all variables, including parental education. The second dataset hence included only newborns whose parents were included in the random sample of one of the Structural Surveys 2010-2014. We also excluded mothers who delivered at age less than 20 years, because education is incomplete at that age.

Statistical and spatial analyses

We fitted linear mixed-effect models (LMEM) to examine the associations between the two outcomes and the neonatal and pregnancy, parental and environmental factors. In the model for birth weight, we log-transformed the outcome and used a cubic spline function with three knots at weeks 25, 30 and 35 to capture the relationship between gestational age and log birth weight. Log transforming the birth weight results in a multiplicative model. Except for gestational age, maternal age and altitude, all predictors were modelled categorically. Maternal age was modelled by a piece-wise linear function, with age group 20 to 30 years as the reference group and separate linear trends for age groups 30-40 years, over 40 years and less than 20 years. Altitude was centred at 500 m and modelled linearly. The random effects in the mixed-effect model captured area-level differences between observed and expected mean outcome, based on the 705 statistical areas [22]. In the main analysis, we fitted four models to the complete-case dataset: Model 0 contained no explanatory variables. Model 1 included birth and pregnancy-related variables: sex, birth rank and gestational age (for the analysis of birth weight). Model 2 additionally included age of the mother, parental education and nationality. Model 3 additionally included geographical variables: altitude, degree of urbanisation and language region.

We displayed mean gestational age and birth weight at area-level on maps and assessed to what extent spatial variation was accounted for by the explanatory variables. Values were categorised into seven intervals symmetric around the mean and color-coded. Spatial autocorrelation of the gestational age and birth weight across regions was tested by global and local Moran's I tests [23]. The global Moran test summarises overall spatial

For peer review only - http://bmjopen.bmj.com/site/about/guidelines.xhtml

autocorrelation and the local test identifies areas that are correlated with neighbouring areas. In the presence of spatial autocorrelation, model estimates are at risk of bias if the autocorrelation is not taken into account.

We performed three sensitivity analyses. First, we accounted for spatial autocorrelation using the Besag-York-Mollier (BYM) model [24] using uninformative gamma-distributed (1, 0.005) priors. The calculations were carried out using the Integrated Nested Laplace Approximation (INLA) approach [25]. Similar results from models with and without the spatial component indicate low bias. Second, we repeated analyses of birth weight without adjusting for gestational age. Third, we repeated analyses of birth weight and gestational age, additionally adjusting for neighbourhood socio-economic position (SEP), using an updated version of the Swiss SEP index, which is based on levels of rent, education and occupation of heads of households and crowding [26]. The updated version of the index is based on data from Structural Surveys 2010-2014, and includes information on income of households in the neighbourhood. We used quintiles of the index in the analysis, with higher quintiles indicating higher SEP.

All analyses and maps were done in R 3.3.2 [27] using packages lme4, maptools, sp, spdep, rgdal, INLA, GISTools, rgeos, raster and ggplot2.

Patient and public involvement

This analysis was based on routine registry data and no patients were involved in developing the research question, outcome measures and overall design of the study. Due to the anonymous nature of the data, we were unable to disseminate the results of the research directly to study participants.

For peer review only - http://bmjopen.bmj.com/site/about/guidelines.xhtml

RESULTS

Characteristics of study populations

A total of 328,349 live births were recorded in Switzerland between 1 January 2011 and 31 December 2014. We excluded non-singleton live births (n=11,835) and those with missing gestational age, birth weight or maternal nationality. The eligible study population therefore included 315,177 singleton live births. The complete case population consisted of 81,968 singleton live births with values available for all predictors including parental education, for which complete data were only available in the Structural Surveys (supplementary Figure S1).

Table 1 shows the distributions of predictors and outcomes in the two study populations. Data about the nationality of fathers was missing for 1.5% of eligible live births. In almost all of these cases, information about the father was missing completely, indicating that the father is unknown to the authorities. Apart from missing data, the distributions of most variables were similar between the two nested datasets. The proportion of Swiss mothers and fathers was higher in the complete case population than in the eligible population. Birth at full term is defined as between 39 and 41 weeks of gestation (273 to 287 days). The mean gestational age in the eligible population was 276 days (SD 12) and the mean birth weight 3328 g (SD 515). The corresponding figures in the complete case population were 276 days (SD 12) and 3339 g (SD 501).

Maps of gestational age and birth weight

Figure 1 presents maps of Switzerland with crude average gestational age and birth weight across the 705 areas. For both outcomes, the maps are broadly similar between the eligible and complete case populations. For gestational age, area-level averages for the eligible population vary between 272 and 279 days. For the complete case population variation was greater, from 268 to 281 days, as expected for a smaller sample. The map shows shorter gestation in the Western, North Western region and Southern (Canton of Ticino) regions of Switzerland, with a patchy pattern in the densely populated areas between the Alps (across the centre) and Jura mountain ranges (to the North West). For birth weight, area-level averages vary between 3138 and 3467g for the eligible population and between 3080 and 3648 g for the complete case population. The maps for birth weight show lower birth weights in the Western and Southern regions of the country. The French and Italian-speaking

For peer review only

regions are in the West and South of Switzerland, with the remainder being German-speaking.

Multivariable analyses

Table 2 shows associations of area-level mean gestational age at birth and mean birth weight with pregnancy, parental and environmental factors from the fully adjusted linear mixed-effects models (model 3). For gestational age, the largest differences were observed across maternal age at birth. Compared to maternal age 20-30 years, gestational age was considerably shorter in teenage mothers, and in mothers aged over 40 years. For example, in mothers aged 15 years, pregnancies were about 4 days shorter, and after age of 40 years, they were about 3 days shorter for each 5-year increase in maternal age. Compared with Swiss fathers, pregnancies were about 4 days shorter if the nationality of the father was missing. Smaller differences in gestational age were observed across categories of sex, birth rank, nationality of the mother, urbanisation and between language regions (Table 2). In the complete case population, lower levels of education were associated with shorter pregnancies. Gestational age at birth was not associated with altitude.

Supplementary Figure S2 shows the relationship between gestational age and birth weight separately for male and female newborns. Male newborns were about 5% heavier than female newborns and birth weight increased with birth order (Table 2). In contrast to gestational age, mother's age was not associated with birth weight. Babies born to mothers or fathers from Northern or Eastern Europe were slightly heavier than babies born to Swiss mothers; birth weights were lowest for babies of fathers with missing nationality. Birth weight slightly decreased with increasing parental educational attainment. Babies born in the French and Italian-speaking regions were lighter than babies born in the German-speaking Switzerland. Finally, birth weight decreased with increasing altitude of residence.

Proportion of spatial variation explained

The fully adjusted model (model 3) for gestational age explained 31% and 39% of the spatial variation across the 705 areas for eligible and complete case populations, respectively. The corresponding figures for birth weight were 87% and 88%. When assessing each factor separately (Table 3), language region alone explained most of the spatial variation for both outcomes. For gestational age, level of urbanisation of the mother's place of residence also explained a considerable part of the variation. Factors that contributed to explaining the

For peer review only - http://bmjopen.bmj.com/site/about/guidelines.xhtml

spatial variation in birth weight were gestational age, parental nationalities, altitude at the mother's place of residence and birth order. Figure 2 illustrates the reduction in the spatial variation of gestational age and birth weight with maps, when moving from model 0 (0% reduction) to models 1, 2 and 3, based on the complete case population.

Spatial autocorrelation and sensitivity analyses

For gestational age, the global Moran's I statistic, based on the complete case dataset and model 0, was I=0.19, with $P<10^{-14}$. After adjusting for all the predictors in model 3 there was still some residual autocorrelation (*I*=0.10, P=0.0004). For birth weight, the corresponding Moran's I statistic was I=0.28, with $P<10^{-15}$. After adjusting for all predictors in model 3 there was little residual autocorrelation (I=0.04, P=0.051). Supplementary Table S2 compares the results from model 3 accounting and not accounting for spatial autocorrelation. The results are similar and the potential bias from residual spatial autocorrelation is therefore unlikely to be a major issue. Repeating analyses of birth weight without adjusting for gestational age produced generally similar coefficients (supplementary Table S3). Associations with maternal age, maternal education and language regions were slightly stronger in model 3 without adjustment for gestational age, possibly because some of their effect was mediated by gestational age. Model 3 without gestational age explained 77% and 76% of the spatial variation in the eligible and complete case population, respectively. The index of neighbourhood SEP was only weakly associated with the two outcomes (Supplementary Table S4), and adjusting for it only slightly increased the amount of spatial variation explained.

For peer review only

DISCUSSION

Our study assessed factors associated with gestational age and birth weight in Switzerland and their contribution to spatial variation, based on routinely collected data. Gestational age at birth was strongly associated with maternal age, missing information on the father and language region. Birth weight was associated with sex, birth rank, missing information on the father, parental education, altitude and language region. The variables included in the fully adjusted model explained more than 80% of the regional variation in birth weight and about 30% of the regional variation in gestational age. Strengths of this study include a large sample with national coverage of the Swiss resident population, as well as the availability of data on several relevant predictors, either on all births or on a large random sample of eligible births. Precise spatial data and spatial statistics allowed us to assess the proportion of area-level variation explained, spatial autocorrelation and gauge the likelihood of bias due to residual autocorrelation.

This study found important spatial variation in both gestational age and birth weight in Switzerland. Language region in Switzerland was the single factor that explained the greatest proportion of spatial variation in gestational age and birth weight. In the French and Italian speaking regions, gestational age was shorter and birth weight lower than in the German speaking part. Language region is a proxy for a wide range of cultural, social and behavioural factors, including diet, smoking and alcohol consumption [28] of parents, as well as their ancestry. In this context it is noteworthy that neighbourhood SEP explained only a small proportion of the spatial variation.

Other factors that could not be measured directly, such as health care provision, might have accounted for some of the unexplained variation. In particular, data at the individual or small area level on the mode of delivery (vaginal or by Caesarean section, induced or spontaneous) were not available. The proportion of live births with Caesarian section as the mode of delivery varies across regions in Switzerland, and it is reasonable to expect that it would explain some of the remaining variation, both in gestational age and birth weight. Specifically, we would expect regions with higher proportions of Caesarian section to have lower mean gestational age (and consequently birthweight). However, the regional rates of Caesarian section published by the Federal Office of Statistics do not match this expectation [29], with urban areas showing some of the highest Caesarian section rates

For peer review only - http://bmjopen.bmj.com/site/about/guidelines.xhtml

For peer review only

but also high mean gestational age and birth weight. In fact, geographical patterns of Caesarean section seem to be largely driven by urban-rural differences. Differences in section rates may have contributed to spatial variation in gestational age, but it seems unlikely that they are an important driver of this variation.

While young and old maternal age are well-known predictors of shorter gestation [30,31], the association we found with missing data on the father's nationality was unexpected. In the vast majority of cases, the information is missing because no father came forward and officially accepted paternity of the child. It is possible that missing data about the father are an indicator of lower socio-economic position and social support of the mother, resulting in greater vulnerability. Studies from the United States of America found a missing name of the father on the infant's birth certificate was associated with lower education, smoking during pregnancy, preterm birth, lower birth weight, no breastfeeding and higher neonatal and post-neonatal mortality [32–35]. Children not recognised by their fathers may thus be a group at higher risk of infant and child morbidity and mothers might benefit from additional care during pregnancy and postnatally.

There are several limitations to our study. We did not have data about maternal health-related behaviours such as smoking [36], mothers' weight and height [36], disease such as gestational diabetes and data on parental genetic factors. Whilst parental nationality and education might have served as crude proxies for some missing variables, individual-level data about these factors would be valuable. A recent large-scale meta-analysis of genome-wide association data indicated that genetic factors influence birth weight through their effects on gestational age, maternal glucose metabolism, cytochrome P450 activity and possibly through effects on maternal immune function and blood pressure [37]. Of note, compared to the foetus who carries maternal and paternal genes, maternal genes exert a larger effect on gestational age and a weaker effect on birth weight [38,39].

Our study also showed associations between mean gestational age and the proportion of preterm births (<37 weeks), as well as mean birth weight and proportion of low birth weight newborns (<2500 g) across the 705 small areas, i.e. associations with conditions that are clinically relevant (Figure S3). However, from a statistical point of view, analyzing means is more robust and powerful than using a binary indicator defined by a cutoff [40]. Finally, we adjusted analyses of birth weight for gestational age, which may

For peer review only - http://bmjopen.bmj.com/site/about/guidelines.xhtml

mediate the effects of other variables, for example maternal age. Adjusting for a variable on the causal pathway has been criticised because it may introduce selection bias (or collider bias in the language of directed acyclic graphs), if there are unknown or unmeasured factors that have an effect on both gestational age and birth weight [41–43]. In our study results were broadly similar with and without adjustment for gestational age and the focus of our study was not on causal inference, but on gaining an understanding of the factors contributing to spatial variation of birth weight and gestational age.

In conclusion, our study identified important differences in mean gestational age and birth weight across Switzerland. Small area variation in birth weight is largely, and in gestational age partially, explained by pregnancy-related, parental, and environmental factors.

For peer review only

For peer review only - http://bmjopen.bmj.com/site/about/guidelines.xhtml

REFERENCES

1 Regev RH, Lusky A, Dolfin T, *et al.* Excess mortality and morbidity among small-for-gestational-age premature infants: a population-based study. *J Pediatr* 2003;**143**:186–91.

2 Wilcox AJ. Birth weight and perinatal mortality: the effect of maternal smoking. *Am J Epidemiol* 1993;**137**:1098–104.

3 Barker DJ, Winter PD, Osmond C, *et al.* Weight in infancy and death from ischaemic heart disease. *Lancet* 1989;**ii**:577–80.http://www.ncbi.nlm.nih.gov/pubmed/2570282 (accessed 3 Feb 2015).

4 Lubchenco LO, Searls DT, Brazie J V. Neonatal mortality rate: relationship to birth weight and gestational age. *J Pediatr* 1972;**81**:814–22.

5 de Bernabé J V., Soriano T, Albaladejo R, *et al.* Risk factors for low birth weight: a review. *Eur J Obstet Gynecol Reprod Biol* 2004;**116**:3–15.

6 Kramer MS. Determinants of low birth weight: methodological assessment and meta-analysis. *Bull World Health Organ* 1987;**65**:663–737.

7 Secker-Walker RH, Vacek PM. Relationships between cigarette smoking during pregnancy, gestational age, maternal weight gain, and infant birthweight. *Addict Behav* 2003;**28**:55–66. doi:10.1016/S0306-4603(01)00216-7

8 Matoba N, Yu Y, Mestan K, *et al.* Differential Patterns of 27 Cord Blood Immune Biomarkers Across Gestational Age. *Pediatrics* 2009;**123**:1320–8. doi:10.1542/peds.2008-1222

9 Diez Roux A V, Mair C. Neighborhoods and health. *Ann N Y Acad Sci* 2010;**1186**:125–45. doi:10.1111/j.1749-6632.2009.05333.x

10 Macintyre S, Ellaway A, Cummins S. Place effects on health: how can we conceptualise, operationalise and measure them? *Soc Sci Med* 2002;**55**:125–39.http://www.ncbi.nlm.nih.gov/pubmed/12137182

11 Clemens T, Dibben C. Living in stressful neighbourhoods during pregnancy: An observational study of crime rates and birth outcomes. *Eur J Public Health* 2017;**27**:197–202. doi:10.1093/eurpub/ckw131

12 Kirby RS, Liu J, Lawson AB, *et al.* Spatio-temporal patterning of small area low birth weight incidence and its correlates: A latent spatial structure approach. *Spat Spatiotemporal Epidemiol* 2011;**2**:265–71. doi:10.1016/j.sste.2011.07.011

For peer review only
For peer review only - http://bmjopen.bmj.com/site/about/guidelines.xhtml

13 Insaf TZ, Talbot T. Identifying areas at risk of low birth weight using spatial epidemiology: A small area surveillance study. *Prev Med (Baltim)* 2016;**88**:108–14. doi:10.1016/j.ypmed.2016.03.019

14 Dibben C, Clemens T. Place of work and residential exposure to ambient air pollution and birth outcomes in Scotland, using geographically fine pollution climate mapping estimates. *Environ Res* 2015;**140**:535–41. doi:10.1016/j.envres.2015.05.010

15 Ghosh JKC, Wilhelm M, Su J, *et al.* Assessing the Influence of Traffic-related Air Pollution on Risk of Term Low Birth Weight on the Basis of Land-Use-based Regression Models and Measures of Air Toxics. *Am J Epidemiol* 2012;**175**:1262–74. doi:10.1093/aje/kwr469

16 Aebi-Popp K, Lapaire O, Glass TR, *et al.* Pregnancy and delivery outcomes of HIV infected women in Switzerland 2003-2008. *J Perinat Med* 2010;**38**:353–8. doi:10.1515/JPM.2010.052

17 Jaeger FN, Hossain M, Kiss L, *et al.* The health of migrant children in Switzerland. *Int J Public Health* 2012;**57**:659–71. doi:10.1007/s00038-012-0375-8

18 Federal Statistical Office. Births. https://www.bfs.admin.ch/bfs/de/home/statistiken/bevoelkerung/geburten-todesfaelle/geburten.html (accessed 18 Sep 2018).

19 Spörri A, Zwahlen M, Egger M, *et al.* The Swiss National Cohort: a unique database for national and international researchers. *Int J Public Heal* 2010;**55**:239–42. doi:10.1007/s00038-010-0160-5

20 Bopp M, Spörri A, Zwahlen M, *et al.* Cohort Profile: the Swiss National Cohort--a longitudinal study of 6.8 million people. *Int J Epidemiol* 2009;**38**:379–84. doi:10.1093/ije/dyn042

21 Federal Statistical Office. Structural Survey. https://www.bfs.admin.ch/bfs/en/home/statistics/population/surveys/se.html (accessed 18 Sep 2018).

22 Federal Statistical Office. Swiss MedStat regions. https://www.bfs.admin.ch/bfs/de/home/statistiken/gesundheit/nomenklaturen/medsreg.html (accessed 18 Sep 2018).

23 Moran PAP. Notes on Continuous Stochastic Phenomena. *Biometrika* 1950;**37**:17–23.http://www.jstor.org/stable/2332142

For peer review only - http://bmjopen.bmj.com/site/about/guidelines.xhtml

24 Besag J, York J, Mollié A. Bayesian image restoration, with two applications in spatial statistics. *Ann Inst Stat Math* 1991;:1–20.

25 Rue H, Martino S, Chopin N. Approximate Bayesian inference for latent Gaussian models by using integrated nested Laplace approximations. *J R Stat Soc Ser B* 2009;**71**:319–92.

26 Panczak R, Galobardes B, Voorpostel M, *et al.* A Swiss neighbourhood index of socioeconomic position: development and association with mortality. *J Epidemiol Community Health* 2012;**66**:1129–36. doi:10.1136/jech-2011-200699

27 The R Core Team: R. A Language and Environment for Statistical Computing. Vienna, Austria. 2014;**ISBN 3-900**.http://www.r-project.org.

28 Faeh D, Minder C, Gutzwiller F, *et al.* Culture, risk factors and mortality: can Switzerland add missing pieces to the European puzzle? *J Epidemiol Community Heal* 2009;**63**:639–645.

29 Federal Office of Statistics. Rate of Caeserian section (MS regions). https://www.bfs.admin.ch/bfs/en/home/statistics/catalogues-databases/maps.assetdetail.4262550.html

30 Kozuki N, Lee AC, Silveira MF, *et al.* The associations of parity and maternal age with small-for-gestational-age, preterm, and neonatal and infant mortality: A meta-analysis. BMC Public Health. 2013;**13**:S2. doi:10.1186/1471-2458-13-S3-S2

31 Restrepo-Méndez MC, Lawlor DA, Horta BL, *et al.* The association of maternal age with birthweight and gestational age: A cross-cohort comparison. *Paediatr Perinat Epidemiol* 2015;**29**:31–40. doi:10.1111/ppe.12162

32 Alio AP, Mbah AK, Kornosky JL, *et al.* Assessing the impact of paternal involvement on racial/ethnic disparities in infant mortality rates. *J Community Health* 2011;**36**:63–8. doi:10.1007/s10900-010-9280-3

33 Gaudino JA, Jenkins B, Rochat RW. No fathers' names: A risk factor for infant mortality in the State of Georgia, Usa. *Soc Sci Med* 1999;**48**:253–65. doi:10.1016/S0277-9536(98)00342-6

34 Cheng ER, Hawkins SS, Rifas-Shiman SL, *et al.* Association of missing paternal demographics on infant birth certificates with perinatal risk factors for childhood obesity. *BMC Public Health* 2016;**16**:1–10. doi:10.1186/s12889-016-3110-1

35 Tan H, Wen SW, Walker M, *et al.* Missing paternal demographics: A novel indicator for

For peer review only - http://bmjopen.bmj.com/site/about/guidelines.xhtml

identifying high risk population of adverse pregnancy outcomes. *BMC Pregnancy Childbirth* 2004;**4**. doi:10.1186/1471-2393-4-21

36 Wen SW, Goldenberg RL, Cutter GR, *et al.* Smoking, maternal age, fetal growth, and gestational age at delivery. *Am J Obstet Gynecol* 1990;**162**:53–8. doi:10.1016/0002-9378(90)90819-S

37 Beaumont RN, Warrington NM, Cavadino A, *et al.* Genome-wide association study of offspring birth weight in 86 577 women identifies five novel loci and highlights maternal genetic effects that are independent of fetal genetics. *Hum Mol Genet* 2018;**27**:742–56. doi:10.1093/hmg/ddx429

38 Beaty TH. Invited commentary: Two studies of genetic control of birth weight where large data sets were available. *Am J Epidemiol* 2007;**165**:753–5. doi:10.1093/aje/kwk106

39 Lunde A, Melve KK, Gjessing HK, *et al.* Genetic and environmental influences on birth weight, birth length, head circumference, and gestational age by use of population-based parent-offspring data. *Am J Epidemiol* 2007;**165**:734–41. doi:10.1093/aje/kwk107

40 Altman DG, Royston P. The cost of dichotomising continuous variables. *BMJ* 2006;**332**:1080.

41 Cole SR, Platt RW, Schisterman EF, *et al.* Illustrating bias due to conditioning on a collider. *Int J Epidemiol* 2010;**39**:417–20. doi:10.1093/ije/dyp334

42 Delbaere I, Vansteelandt S, De Bacquer D, *et al.* Should we adjust for gestational age when analysing birth weights? The use of z-scores revisited. *Hum Reprod* 2007;**22**:2080–3. doi:10.1093/humrep/dem151

43 Wilcox AJ, Weinberg CR, Basso O. On the pitfalls of adjusting for gestational age at birth. *Am J Epidemiol* 2011;**174**:1062–8. doi:10.1093/aje/kwr230

For peer review only

For peer review only - http://bmjopen.bmj.com/site/about/guidelines.xhtml

Table 1. Characteristics of complete case and eligible study populations.

	Eligible population			Complete case population		
		Gest. age (days)	Birth weight (g)		Gest. age (days)	Birth weight (g)
	No. (%)	Mean (SD)	Mean (SD)	No. (%)	Mean (SD)	Mean (SD)
**Total**	315'177 (100%)	276 (12)	3328 (515)	81'968 (100%)	276 (12)	3339 (501)
**Birth weight (g)**						
<1500	2141 (0.7%)	196 (27)	966 (354)	445 (0.5%)	198 (28)	983 (491)
1500-1999	2413 (0.8%)	238 (15)	1800 (142)	612 (0.7%)	239 (15)	1803 (528)
2000-2499	10036 (3.2%)	258 (14)	2312 (134)	2484 (3%)	258 (13)	2314 (477)
≥ 2500	300586 (95.4%)	277 (9)	3391 (423)	78426 (95.7%)	277 (9)	3396 (502)
**Gestation. age (weeks)**						
$<32^0$	2333 (0.7%)	195 (23)	1108 (527)	487 (0.6%)	196 (23)	1134 (491)
32^0-34^6	3950 (1.3%)	237 (6)	2144 (424)	961 (1.2%)	237 (6)	2136 (470)
35^0-36^6	10907 (3.5%)	253 (4)	2686 (431)	2760 (3.4%)	253 (4)	2692 (489)
$\geq 37^0$	297987 (94.5%)	278 (8)	3385 (440)	77760 (94.9%)	278 (8)	3390 (502)
**Sex**						
Female	152757 (48.5%)	276 (12)	3260 (494)	39823 (48.6%)	276 (11)	3267 (502)
Male	162420 (51.5%)	275 (13)	3392 (525)	42145 (51.4%)	276 (12)	3406 (501)
**Birth rank**						
1	155739 (49.4%)	276 (13)	3262 (519)	37763 (46.1%)	276 (13)	3267 (498)
2	115440 (36.6%)	275 (11)	3382 (497)	32315 (39.4%)	276 (11)	3386 (504)
3	34364 (10.9%)	275 (11)	3418 (509)	9360 (11.4%)	275 (11)	3430 (508)
≥ 4	9634 (3.1%)	275 (12)	3438 (537)	2530 (3.1%)	275 (11)	3459 (498)
**Civil status**						
Married	250055 (79.3%)	276 (12)	3345 (508)	69465 (84.7%)	276 (12)	3349 (501)
Not married	65122 (20.7%)	276 (14)	3262 (536)	12503 (15.3%)	276 (13)	3283 (503)
**Maternal age (years)**						
mean (SD)	*31.7 (5.0)*			*32.2 (4.7)*		
< 20	2679 (0.8%)	275 (16)	3224 (554)	0 (0%)	-	-
≥ 20-25	28615 (9.1%)	277 (12)	3317 (511)	5417 (6.6%)	277 (12)	3337 (491)
≥ 25-30	82620 (26.2%)	276 (12)	3330 (506)	20771 (25.3%)	276 (12)	3337 (500)
≥ 30-35	118303 (37.5%)	276 (12)	3335 (510)	32771 (40%)	276 (12)	3341 (505)
≥ 35-40	67914 (21.5%)	275 (12)	3333 (523)	19052 (23.2%)	275 (11)	3345 (497)
≥ 40	15046 (4.8%)	273 (14)	3286 (555)	3957 (4.8%)	273 (14)	3295 (512)
**Nationality mother**						
Switzerland	194570 (61.7%)	276 (12)	3322 (511)	55591 (67.8%)	276 (12)	3331 (502)
Southern Europe	23585 (7.5%)	275 (12)	3251 (494)	5761 (7%)	276 (11)	3261 (502)
Western Europe	26005 (8.3%)	276 (12)	3348 (516)	6495 (7.9%)	276 (12)	3359 (508)
Northern Europe	3695 (1.2%)	276 (13)	3418 (510)	850 (1%)	276 (13)	3414 (508)
Eastern Europe	38762 (12.3%)	276 (13)	3397 (523)	8035 (9.8%)	276 (12)	3422 (499)
Other	28560 (9.1%)	275 (14)	3313 (535)	5236 (6.4%)	275 (13)	3332 (492)
**Nationality father**						
Switzerland	191589 (60.8%)	276 (12)	3329 (506)	55432 (67.6%)	276 (12)	3336 (502)
Southern Europe	31466 (10%)	275 (12)	3256 (493)	7970 (9.7%)	275 (11)	3262 (504)
Western Europe	26954 (8.6%)	276 (12)	3353 (518)	6661 (8.1%)	276 (12)	3367 (514)
Northern Europe	3911 (1.2%)	276 (12)	3406 (510)	887 (1.1%)	276 (13)	3393 (499)
Eastern Europe	35387 (11.2%)	276 (13)	3397 (528)	7229 (8.8%)	276 (12)	3418 (489)
Other	21077 (6.7%)	276 (13)	3307 (531)	3789 (4.6%)	276 (12)	3319 (497)
missing	4793 (1.5%)	272 (23)	3148 (693)	-	-	-
**Education mother**						
Tertiary	42088 (13.4%)	276 (12)	3344 (500)	33505 (40.9%)	276 (12)	3347 (500)
Secondary	48878 (15.5%)	276 (12)	3328 (509)	38382 (46.8%)	276 (12)	3331 (502)
Compulsory	14642 (4.6%)	275 (13)	3329 (534)	10081 (12.3%)	275 (13)	3336 (503)
Unknown (age <20 yrs)	2679 (0.8%)	275 (16)	3224 (554)	0 (0%)	-	-
missing	206890 (65.6%)	276 (12)	3326 (517)	-	-	-
**Education father**						
Tertiary	49848 (15.8%)	276 (12)	3348 (497)	40345 (49.2%)	276 (12)	3350 (500)
Secondary	41301 (13.1%)	276 (12)	3323 (511)	32118 (39.2%)	276 (12)	3327 (504)
Compulsory	13731 (4.4%)	276 (12)	3323 (514)	9505 (11.6%)	276 (12)	3330 (500)
missing	210297 (66.7%)	276 (13)	3325 (519)	-	-	-
**Altitude (m)**						
mean (SD)	*515 (189)*			*511 (180)*		
**Urbanisation**						
Urban	96643 (30.7%)	276 (13)	3326 (517)	22770 (27.8%)	276 (12)	3334 (502)
Peri-urban	138826 (44%)	275 (12)	3329 (514)	36629 (44.7%)	276 (12)	3339 (502)
Rural	79708 (25.3%)	276 (12)	3329 (512)	22569 (27.5%)	276 (12)	3343 (500)
**Language region**						
German	223586 (70.9%)	276 (12)	3348 (515)	54106 (66%)	276 (12)	3362 (502)
French	80068 (25.4%)	275 (12)	3283 (512)	23579 (28.8%)	275 (12)	3296 (501)
Italian	11523 (3.7%)	275 (12)	3252 (494)	4283 (5.2%)	275 (11)	3268 (500)
**Socio-economic position**						
1st quintile	63230 (20.1%)	276 (12)	3318 (522)	15752 (19.2%)	276 (12)	3331 (501)
2nd quintile	63199 (20.1%)	276 (12)	3324 (519)	16034 (19.6%)	276 (12)	3334 (505)
3rd quintile	63156 (20%)	276 (12)	3329 (516)	16555 (20.2%)	276 (12)	3337 (500)
4th quintile	62970 (20%)	276 (12)	3335 (509)	16933 (20.7%)	276 (12)	3344 (500)
5th quintile	62622 (19.9%)	276 (12)	3335 (507)	16694 (20.4%)	276 (12)	3346 (502)

For peer review only - http://bmjopen.bmj.com/site/about/guidelines.xhtml

Table 2. Associations of mean gestational age at birth and mean birth weight with pregnancy, parental and environmental factors from adjusted linear mixed-effects model (model 3).

	Gestational age (days) Absolute differences (95% CI)		Birth weight (g) * Relative differences (95% CI)	
	**Eligible population**	**Complete case population**	**Eligible population**	**Complete case population**
**Intercept**	277.3 (277.2 to 277.5)	277.9 (277.7 to 278.2)	3278 (3218 to 3339)&	3298 (3180 to 3420) &
**Sex**				
Female	0	0	1	1
Male	-0.56 (-0.65, -0.48)	-0.63 (-0.79, -0.47)	1.045 (1.044 to 1.046)	1.048 (1.046, 1.049)
**Birth rank**				
1¶	0	0	1	1
2	-0.39 (-0.49, -0.29)	-0.34 (-0.52, -0.16)	1.038 (1.037, 1.039)	1.039 (1.037, 1.041)
3	-0.37 (-0.52, -0.22)	-0.16 (-0.44, 0.11)	1.050 (1.048, 1.051)	1.054 (1.051, 1.057)
≥ 4	-0.24 (-0.50, 0.02)	0.24 (-0.25, 0.72)	1.058 (1.056, 1.061)	1.065 (1.059, 1.070)
**Age mother (yrs)‡**				
< 20 (per 5 yrs decr.)	-4.10 (-5.59, -2.61)	-	1.002 (0.987, 1.017)	-
≥ 20-30¶	0	0	1	1
≥ 30-40 (per 5 yrs)	-0.99 (-1.06, -0.91)	-0.93 (-1.07, -0.78)	1.000 (1.000, 1.001)	0.998 (0.997, 1.000)
≥ 40 (per 5 yrs)	-2.93 (-3.36, -2.50)	-3.46 (-4.29, -2.63)	0.998 (0.994, 1.003)	0.998 (0.990, 1.006)
**Civil status◊**				
Married	0	0	1	1
Not married	-0.01 (-0.13, 0.10)	0.15 (-0.08, 0.38)	0.990 (0.989, 0.991)	0.993 (0.99, 0.995)
**Nationality mother**				
Switzerland¶	0	0	1	1
S Europe	0.20 (-0.01, 0.40)	0.39 (00, 0.78)	0.994 (0.992, 0.996)	0.995 (0.991, 0.999)
W Europe	0.20 (0.02, 0.38)	-0.08 (-0.43, 0.26)	1.008 (1.006, 1.010)	1.007 (1.004, 1.011)
N Europe	0.37 (-0.07, 0.81)	0.30 (-0.57, 1.17)	1.025 (1.020, 1.029)	1.022 (1.013, 1.031)
E Europe	0.21 (0.04, 0.38)	0.33 (-0.01, 0.68)	1.013 (1.011, 1.014)	1.017 (1.014, 1.021)
Other	-0.32 (-0.49, -0.14)	-0.67 (-1.05, -0.30)	1.007 (1.005, 1.008)	1.012 (1.008, 1.016)
**Nationality father**				
Switzerland¶	0	0	1	1
S Europe	-0.46 (-0.64, -0.28)	-0.28 (-0.62, 0.06)	0.991 (0.990, 0.993)	0.993 (0.989, 0.996)
W Europe	0.07 (-0.11, 0.25)	0.30 (-0.04, 0.63)	1.008 (1.006, 1.009)	1.006 (1.003, 1.010)
N Europe	0.51 (0.08, 0.94)	-0.24 (-1.09, 0.62)	1.013 (1.009, 1.017)	1.011 (1.003, 1.020)
E Europe	-0.46 (-0.64, -0.28)	-0.01 (-0.38, 0.36)	1.009 (1.007, 1.010)	1.011 (1.008, 1.015)
Other	-0.02 (-0.22, 0.18)	0.48 (0.05, 0.90)	0.992 (0.991, 0.994)	0.992 (0.987, 0.996)
missing	-3.87 (-4.24, -3.50)	-	0.989 (0.985, 0.992)	-
**Education mother**				
Tertiary¶		0		1
Secondary		-0.55 (-0.74, -0.36)		0.996 (0.995, 0.998)
Compulsory		-0.90 (-1.22, -0.58)		0.993 (0.990, 0.996)
**Education father**				
Tertiary¶		0		1
Secondary		-0.16 (-0.35, 0.03)		0.996 (0.994, 0.998)
Compulsory		-0.25 (-0.58, 0.07)		0.997 (0.994, 1.000)
**Altitude (m)**				
500¶	0	0	1	1
per 500 m increase	0.07 (-0.09, 0.23)	0.03 (-0.24, 0.30)	0.989 (0.988, 0.991)	0.989 (0.987, 0.992)
**Urbanization**				
Urban¶	0	0	1	1
Peri-urban	-0.43 (-0.57, -0.28)	-0.59 (-0.82, -0.36)	1.001 (1.000, 1.002)	1.003 (1.000, 1.005)
Rural	-0.15 (-0.32, 0.02)	-0.29 (-0.55, -0.02)	1.000 (0.998, 1.001)	1.003 (1.001, 1.006)
**Language region**				
German¶	0	0	1	1
French	-0.62 (-0.77, -0.47)	-0.66 (-0.88, -0.44)	0.989 (0.987, 0.990)	0.988 (0.985, 0.990)
Italian	-0.94 (-1.26, -0.63)	-1.11 (-1.55, -0.68)	0.982 (0.980, 0.985)	0.983 (0.979, 0.987)
**Percent of spatial variance explained†**	31%	39%	87%	88%

*Birth weight was modelled on a log scale, which results in multiplicative effects. The model for birth weight was additionally adjusted for gestational age by a cubic spline function with knots at weeks 25, 30 and 35.

& In the model for BW, the intercept corresponds to an estimated mean birth weight (g) for a singleton girl born at gestational age 40 weeks as the first child (rank 1) in a German-speaking, urban region of elevation 500m, whose mother is 20-30 years old at birth and married, and both parents have Swiss nationality and tertiary education.

¶ Reference category

‡ Age modelled by a piece-wise linear function: constant at reference range ≥20-30, and separate slopes for age <20, ≥30-40, and ≥40.

◊ Married or in registered partnership / Not married: Single, widow, divorced or in dissolved partnership

† Percentage of regional variance explained by model predictors, i.e. percent reduction in variance of random effects (σ^2) when compared to model with no predictors (model 0).

For peer review only - http://bmjopen.bmj.com/site/about/guidelines.xhtml

Table 3. Percentage of spatial variation explained by each individual variable and explained in addition after adjusting for all other variables.

		Gestational age		Birth weight	
**Spatial variation explained**		**Eligible population**	**Complete case population**	**Eligible population**	**Complete case population**
**By single variables**					
**Pregnancy factors**	Gestational age	-	-	27%	34%
	Sex	0%	0%	1%	2%
	Birth rank	0%	1%	4%	0%
**Parental factors**	Maternal age	0%	1%	1%	1%
	Civil status	0%	0%	10%	5%
	Nationality mother	1%	3%	17%	17%
	Nationality father	3%	4%	25%	20%
	Nationality parents*	3%	5%	27%	23%
	Education mother	-	1%	-	0%
	Education father	-	1%	-	1%
	Education parents*	-	1%	-	1%
**Regional factors**	Altitude	0%	0%	10%	6%
	Urbanization	10%	12%	0%	0%
	Language region	23%	25%	62%	63%
**In addition to all other variables**					
**Pregnancy factors**	Gestational age	-	-	12%	12%
	Sex	0%	0%	0%	1%
	Birth rank	1%	0%	3%	1%
**Parental factors**	Maternal age	0%	1%	0%	0%
	Civil status	0%	0%	0%	0%
	Nationality mother	0%	0%	1%	2%
	Nationality father	1.5%	0%	1%	0%
	Nationality parents*	2.5%	0%	3%	4%
	Education mother	-	2%	-	0%
	Education father	-	0%	-	0%
	Education parents*	-	2%	-	1%
**Regional factors**	Altitude	0%	0%	9%	4%
	Urbanization	9%	10%	0%	1%
	Language region	17%	21%	22%	24%
	**Model 3 (full)**	**31%**	**39%**	**87%**	**88%**

- Data not available
* Nationality or educational attainment of both mother and father were entered into the model.

For peer review only

FIGURE LEGENDS

Figure 1. Maps of average gestational age (upper two panels) and birth weight (lower two panels) observed across 705 Swiss areas. Left: all eligible live births (n=315,177), right: complete case population (n=81,968). Note that 277 days correspond to $39^{4/7}$ weeks. The orientation of the maps is standard, with North being up.

Figure 2. Maps of gestational age and birth weight from crude model (model 0) and multivariable linear mixed-effect models (models 1-3) with percent reduction in the regional variation, represented by random effects. Analyses based on complete case population (*N* = 81,968). The orientation of the maps is standard, with North being up.

FOOTNOTES

Contributors: ME and CEK conceived the study and obtained funding. VS, ME, MZ and CEK developed the analysis plan. VS did all statistical analyses and wrote the first draft of the paper, which was revised by ME taking into account the critical comments from CEK, MZ, MA and NL. ME supervised the study. All authors approved the final version of the report.

Funding: The Swiss National Cohort is funded by the Swiss National Science Foundation (SNSF) cohort grant No. 148415. The current analysis was funded by SNSF project grant No. 163452. ME was supported by special SNSF project funding (grant No. 174281).

Competing interests: None declared.

Patient consent: Not required.

Ethics approval: The SNC has been approved by the Ethics Committee of the Canton of Bern.

Provenance and peer review: Not commissioned; externally peer reviewed.

Data sharing statement: Data are available within the framework of a data sharing agreement.

For peer review only

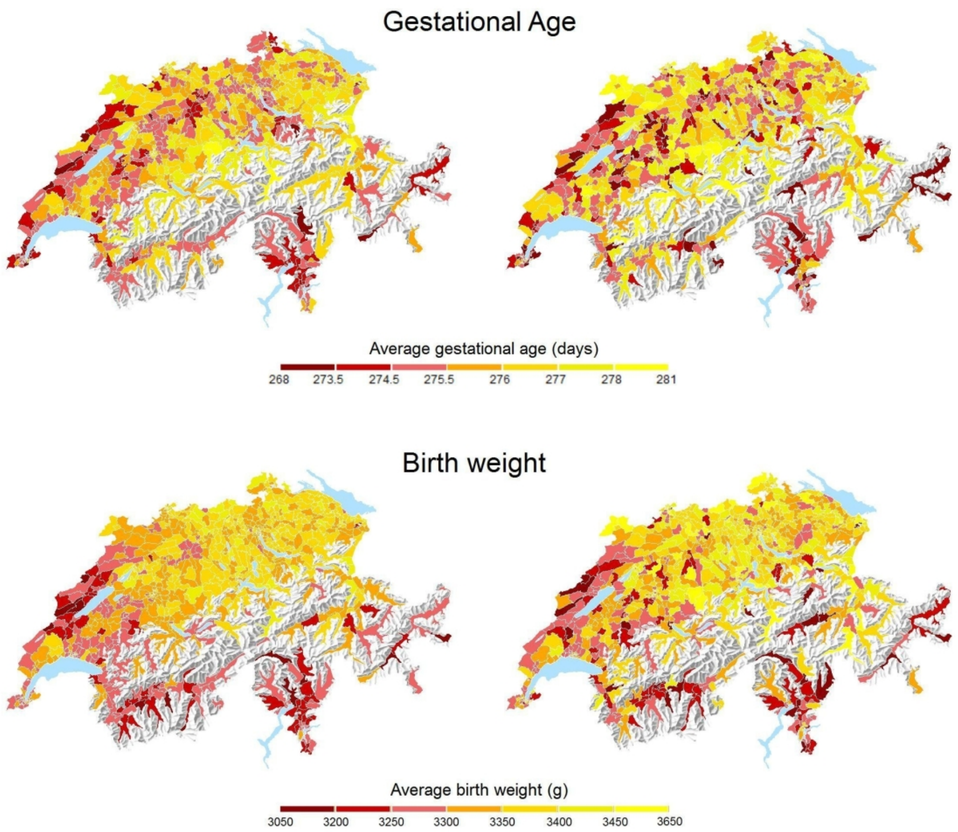

Figure 1. Maps of average gestational age (upper two panels) and birth weight (lower two panels) observed across 705 Swiss areas. Left: all eligible live births (n=315,177), right: complete case population (n=81,968). Note that 277 days correspond to 394/7 weeks. The orientation of the maps is standard, with North being up.

172x152mm (300 x 300 DPI)

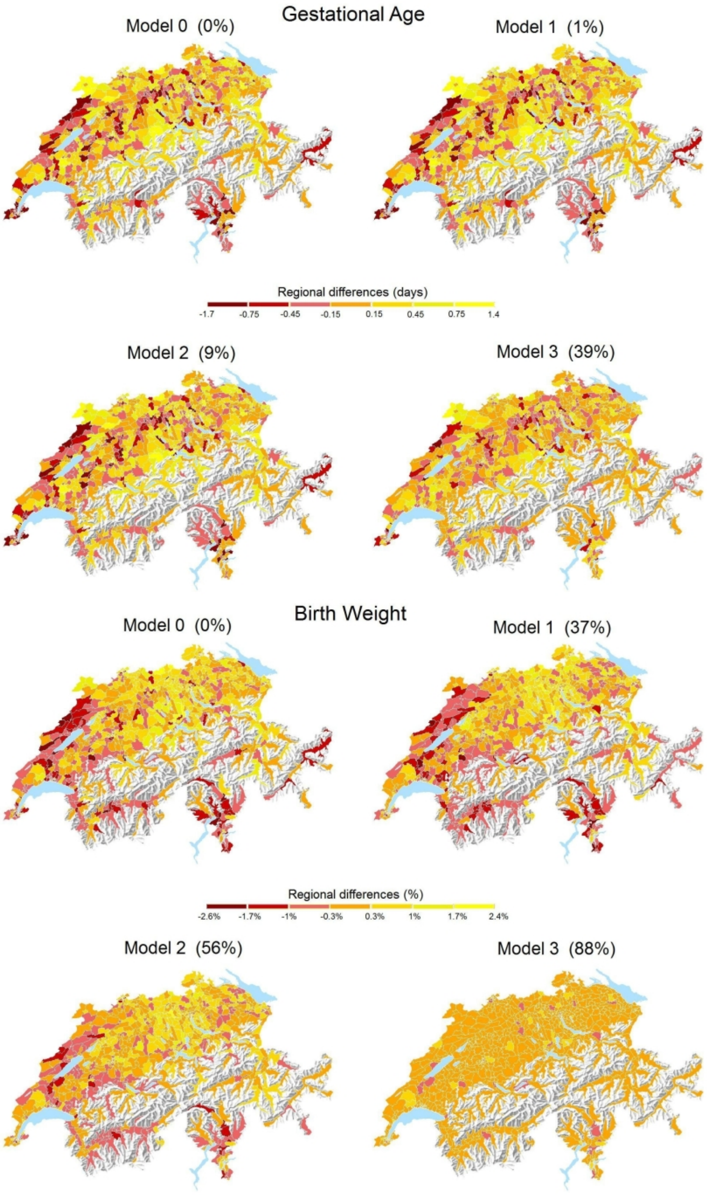

Figure 2. Maps of gestational age and birth weight from crude model (model 0) and multivariable linear mixed-effect models (models 1-3) with percent reduction in the regional variation, represented by random effects. Analyses based on complete case population (N = 81,968). The orientation of the maps is standard, with North being up.

172x279mm (300 x 300 DPI)

Supplementary Table S1. Number of live births, mean gestational age and mean birth weight by maternal nationality in the eligible population (N = 315'177); 276 days correspond to $39^{3/7}$ weeks.

Nationality	N	Gestational age (days) Mean (SD)	Birth weight (g) Mean (SD)
**Switzerland**	194,570	276 (12)	3322 (511)
**Southern Europe**			
**Andorra**	1	279 (-)	3080 (-)
**Italy**	8337	275 (12)	3271 (496)
**Malta**	13	273 (8)	3188 (427)
**Portugal**	12,368	276 (12)	3235 (493)
**San Marino**	2	274 (1.4)	3485 (120)
**Spain**	2864	276 (12)	3263 (488)
**Western Europe**			
**Austria**	1555	275 (14)	3328 (528)
**Belgium**	583	276 (12)	3357 (482)
**Germany**	16,736	276 (13)	3369 (517)
**France**	6173	276 (12)	3294 (505)
**Lichtenstein**	100	275 (11)	3369 (488)
**Luxembourg**	55	276 (19)	3396 (636)
**Netherlands**	803	276 (12)	3377 (529)
**Northern Europe**			
**Denmark**	271	276 (13)	3383 (511)
**Estonia**	81	279 (7)	3601 (466)
**Finland**	312	276 (11)	3465 (523)
**Ireland**	212	276 (16)	3446 (548)
**Iceland**	31	272 (25)	3180 (775)
**Latvia**	187	279 (9)	3493 (434)
**Lithuania**	152	277 (13)	3450 (535)
**Norway**	110	275 (11)	3390 (525)
**Sweden**	571	276 (12)	3422 (470)
**UK**	1768	276 (13)	3397 (513)
**Eastern Europe**			
**Czech Republic**	623	275 (12)	3339 (499)
**Hungary**	913	275 (13)	3341 (512)
**Poland**	1778	276 (12)	3399 (497)
**Slovakia**	1068	276 (12)	3348 (509)
**Albania**	209	276 (12)	3406 (476)
**Bosnia & Herzegovina**	1952	276 (12)	3466 (492)
**Croatia**	1582	276 (12)	3448 (540)
**Kosovo**	10,278	276 (13)	3421 (530)
**Macedonia**	5842	276 (13)	3392 (514)
**Montenegro**	212	276 (10)	3416 (466)
**Serbia**	5195	276 (13)	3400 (536)
**Serbia & Montenegro**	10	277 (8)	3637 (250)
**Slovenia**	163	275 (15)	3366 (589)
**Cyprus**	15	278 (8)	3411 (525)
**Bulgaria**	406	273 (15)	3291 (559)
**Greece**	375	274 (13)	3317 (516)
**Romania**	971	274 (14)	3284 (537)
**Turkey**	4441	275 (13)	3347 (523)
**Belarus**	172	277 (13)	3385 (508)
**Moldova**	135	276 (10)	3496 (515)
**Russia**	1567	277 (12)	3427 (513)
**Ukraine**	855	277 (11)	3412 (473)
**Other (non-Europe)**			
6 most numerous: **Eritrea**	2600	279 (14)	3380 (528)
**Brazil**	2381	274 (12)	3312 (498)
**Sri Lanka**	1391	273 (14)	3158 (553)
**USA**	1291	276 (14)	3378 (532)
**China**	1293	276 (13)	3425 (541)
**Morocco**	1159	276 (14)	3378 (536)
. . .			
**Total**	315,177	276 (12)	3328 (515)

For peer review only

Supplementary Table S2. Comparison of results from fully adjusted model (model 3) accounting and not accounting for spatial autocorrelation. Based on complete-case population (*N* = 81,968).

	Accounting for spatial autocorrelation		Not accounting for spatial autocorrelation	
	Gestational age (days) Absolute differences (95% CI)	Birth weight (g) * Relative differences (95% CI)	Gestational age (days) Absolute differences (95% CI)	Birth weight (g) * Relative differences (95% CI)
Intercept	277.9 (277.6 to 278.1)	3293 (3163 to 3427)	277.9 (277.7 to 278.2)	3298 (3180 to 3420) &
**Sex**				
¶Female	0		0	1
Male	-0.62 (-0.78, -0.46)	1.048 (1.046, 1.049)	-0.63 (-0.79, -0.47)	1.048 (1.046, 1.049)
**Birth rank**				
1¶	0		0	1
2	-0.34 (-0.52, -0.17)	1.039 (1.037, 1.041)	-0.34 (-0.52, -0.16)	1.039 (1.037, 1.041)
3	-0.17 (-0.45, 0.10)	1.054 (1.051, 1.056)	-0.16 (-0.44, 0.11)	1.054 (1.051, 1.057)
≥ 4	0.23 (-0.26, 0.71)	1.065 (1.059, 1.070)	0.24 (-0.25, 0.72)	1.065 (1.059, 1.070)
**Maternal age (yrs)‡**				
≥ 20-30¶	0		0	1
≥ 30-40 yrs (per 5 yrs)	-0.92 (-1.06, -0.77)	0.998 (0.996, 1.000)	-0.93 (-1.07, -0.78)	0.998 (0.997, 1.000)
≥ 40 yrs (per 5 yrs)	-3.48 (-4.30, -2.66)	0.998 (0.990, 1.006)	-3.46 (-4.29, -2.63)	0.998 (0.990, 1.006)
**Civil status◊**				
Married¶	0		0	1
Not married	0.15 (-0.08, 0.38)	0.993 (0.990, 0.995)	0.15 (-0.08, 0.38)	0.993 (0.990, 0.995)
**Nationality mother**				
Switzerland¶	0		0	1
S Europe	0.38 (0.00, 0.77)	0.995 (0.992, 0.999)	0.39 (0.00, 0.78)	0.995 (0.991, 0.999)
W Europe	-0.08 (-0.42, 0.25)	1.007 (1.003, 1.010)	-0.08 (-0.43, 0.26)	1.007 (1.004, 1.011)
N Europe	0.30 (-0.56, 1.17)	1.022 (1.013, 1.031)	0.30 (-0.57, 1.17)	1.022 (1.013, 1.031)
E Europe	0.33 (-0.01, 0.67)	1.017 (1.013, 1.021)	0.33 (-0.01, 0.68)	1.017 (1.014, 1.021)
Other	-0.66 (-1.03, -0.29)	1.012 (1.008, 1.015)	-0.67 (-1.05, -0.30)	1.012 (1.008, 1.016)
**Nationality father**				
Switzerland¶	0		0	1
S Europe	-0.28 (-0.61, 0.06)	0.992 (0.989, 0.996)	-0.28 (-0.62, 0.06)	0.993 (0.989, 0.996)
W Europe	0.30 (-0.03, 0.64)	1.006 (1.002, 1.009)	0.30 (-0.04, 0.63)	1.006 (1.003, 1.010)
N Europe	-0.21 (-1.06, 0.63)	1.011 (1.002, 1.019)	-0.24 (-1.09, 0.62)	1.011 (1.003, 1.020)
E Europe	-0.02 (-0.38, 0.35)	1.011 (1.007, 1.015)	-0.01 (-0.38, 0.36)	1.011 (1.008, 1.015)
Other	0.49 (0.06, 0.91)	0.991 (0.987, 0.996)	0.48 (0.05, 0.90)	0.992 (0.987, 0.996)
**Education mother**				
Tertiary¶			0	1
Secondary	-0.56 (-0.75, -0.37)	0.997 (0.995, 0.999)	-0.55 (-0.74, -0.36)	0.996 (0.995, 0.998)
Compulsory	-0.92 (-1.23, -0.60)	0.993 (0.990, 0.997)	-0.90 (-1.22, -0.58)	0.993 (0.990, 0.996)
**Education father**				
Tertiary¶			0	1
Secondary	-0.16 (-0.35, 0.03)	0.997 (0.995, 0.999)	-0.16 (-0.35, 0.03)	0.996 (0.994, 0.998)
Compulsory	-0.25 (-0.57, 0.07)	0.997 (0.994, 1.001)	-0.25 (-0.58, 0.07)	0.997 (0.994, 1.000)
**Altitude (m)**				
500¶	0		0	1
per 500 m increase	-0.05 (-0.33, 0.24)	0.991 (0.987, 0.994)	0.03 (-0.24, 0.30)	0.989 (0.987, 0.992)
**Urbanization**				
Urban¶	0		0	1
Peri-urban	-0.54 (-0.75, -0.33)	1.001 (0.998, 1.004)	-0.59 (-0.82, -0.36)	1.003 (1.000, 1.005)
Rural	-0.25 (-0.50, 0.00)	1.003 (0.999, 1.006)	-0.29 (-0.55, -0.02)	1.003 (1.001, 1.006)
**Language region**				
German¶	0		0	1
French	-0.33 (-0.75, 0.09)	0.991 (0.983, 0.998)	-0.66 (-0.88, -0.44)	0.988 (0.985, 0.990)
Italian	-1.10 (-1.50, -0.70)	0.984 (0.978, 0.989)	-1.11 (-1.55, -0.68)	0.983 (0.979, 0.987)

*Birth weight was modelled on a log scale, which results in multiplicative effects. The model for birth weight was additionally adjusted for gestational age by a cubic spline function with knots at weeks 25, 30 and 35.

& In the model for BW, the intercept corresponds to an estimated mean birth weight (g) for a singleton girl born at gestational age 40 weeks as the first child (rank 1) in a German-speaking, urban region of elevation 500 m, whose mother is 20-30 years old at birth and married, and both parents have Swiss nationality and tertiary education.

¶ Reference category

‡ Age modelled by a piece-wise linear function: constant at reference range ≥20-30, and separate slopes for age <20, ≥30-40, and ≥40. For ages ≥40, the total estimated effect is hence addition of 10-year effect in age group ≥30-40 plus the corresponding effect in age-group ≥40.

† Percentage of regional variance explained by model predictors, i.e. percent reduction in variance of random effects (σ^2) when compared to model with no predictors (model 0).

For peer review only

Supplementary Table S3. Comparison of results from model (model 3) for birth weight, adjusted and not adjusted for gestational age.

	Birth weight - Model 3 without Gestational Age		Birth weight - Model 3 with Gestational Age*	
	Relative differences (95% CI)		Relative differences (95% CI)	
	Eligible population	Complete case population	Eligible population	Complete case population
**Intercept**	3188 (3181 to 3195)	3209 (3196 to 3222)	3278 (3218 to 3339)[&]	3298 (3180 to 3420)[&]
**Sex**				
¶Female	1	1	1	1
Male	1.04 (1.038, 1.041)	1.041 (1.039, 1.044)	1.045 (1.044 to 1.046)	1.048 (1.046, 1.049)
**Rank**				
1¶	1	1	1	1
2	1.039 (1.038, 1.041)	1.041 (1.038, 1.044)	1.038 (1.037, 1.039)	1.039 (1.037, 1.041)
3	1.052 (1.049, 1.054)	1.058 (1.054, 1.063)	1.050 (1.048, 1.051)	1.054 (1.051, 1.057)
≥ 4	1.060 (1.056, 1.064)	1.071 (1.063, 1.079)	1.058 (1.056, 1.061)	1.065 (1.059, 1.070)
**Age mother (yrs)‡**				
< 20yrs (per 5 yrs decr.)	0.956 (0.935, 0.978)	-	1.002 (0.987, 1.017)	-
≥ 20-30 yrs¶	1	1	1	1
≥ 30-40 yrs (per 5 yrs)	0.991 (0.990, 0.992)	0.990 (0.988, 0.993)	1.000 (1.000, 1.001)	0.998 (0.997, 1.000)
≥ 40 yrs (per 5 yrs)	0.973 (0.967, 0.979)	0.967 (0.955, 0.979)	0.998 (0.994, 1.003)	0.998 (0.990, 1.006)
**Civil status**[◊]				
Married¶	1	1	1	1
Not married	0.989 (0.987, 0.990)	0.992 (0.989, 0.996)	0.990 (0.989, 0.991)	0.993 (0.99, 0.995)
**Nationality mother**				
Switzerland¶	1	1	1	1
S Europe	0.996 (0.993, 0.999)	0.999 (0.993, 1.005)	0.994 (0.992, 0.996)	0.995 (0.991, 0.999)
W Europe	1.010 (1.007, 1.013)	1.006 (1.001, 1.011)	1.008 (1.006, 1.010)	1.007 (1.004, 1.011)
N Europe	1.027 (1.020, 1.034)	1.024 (1.010, 1.037)	1.025 (1.020, 1.029)	1.022 (1.013, 1.031)
E Europe	1.014 (1.011, 1.016)	1.020 (1.015, 1.026)	1.013 (1.011, 1.014)	1.017 (1.014, 1.021)
Other	1.003 (1.000, 1.006)	1.005 (0.999, 1.010)	1.007 (1.005, 1.008)	1.012 (1.008, 1.016)
**Nationality father**				
Switzerland¶	1	1	1	1
S Europe	0.988 (0.985, 0.991)	0.991 (0.986, 0.996)	0.991 (0.990, 0.993)	0.993 (0.989, 0.996)
W Europe	1.008 (1.005, 1.011)	1.009 (1.004, 1.014)	1.008 (1.006, 1.009)	1.006 (1.003, 1.010)
N Europe	1.017 (1.010, 1.024)	1.007 (0.994, 1.019)	1.013 (1.009, 1.017)	1.011 (1.003, 1.020)
E Europe	1.003 (1.000, 1.006)	1.010 (1.004, 1.015)	1.009 (1.007, 1.010)	1.011 (1.008, 1.015)
Other	0.990 (0.987, 0.993)	0.997 (0.990, 1.003)	0.992 (0.991, 0.994)	0.992 (0.987, 0.996)
missing	0.933 (0.928, 0.938)	-	0.989 (0.985, 0.992)	-
**Education mother**				
Tertiary¶	-	1	-	1
Secondary	-	0.992 (0.989, 0.995)	-	0.996 (0.995, 0.998)
Compulsory	-	0.984 (0.979, 0.989)	-	0.993 (0.990, 0.996)
**Education father**				
Tertiary¶	-	1	-	1
Secondary	-	0.995 (0.992, 0.997)	-	0.996 (0.994, 0.998)
Compulsory	-	0.995 (0.990, 1.000)	-	0.997 (0.994, 1.000)
**Altitude (m)**				
500 m¶	1	1	1	1
per 500 m increase	0.990 (0.988, 0.992)	0.989 (0.985, 0.993)	0.989 (0.988, 0.991)	0.989 (0.987, 0.992)
**Urbanization**				
Urban¶	1	1	1	1
Peri-urban	0.998 (0.996, 1.000)	0.998 (0.995, 1.002)	1.001 (1.000, 1.002)	1.003 (1.000, 1.005)
Rural	0.999 (0.996, 1.001)	1.001 (0.997, 1.005)	1.000 (0.998, 1.001)	1.003 (1.001, 1.006)
**Language region**				
[R]German¶	1	1	1	1
French	0.985 (0.983, 0.987)	0.983 (0.980, 0.986)	0.989 (0.987, 0.990)	0.988 (0.985, 0.990)
Italian	0.977 (0.973, 0.981)	0.976 (0.970, 0.982)	0.982 (0.980, 0.985)	0.983 (0.979, 0.987)
**% variation explained**				
Model 3	77%	76%	87%	88%
Model 2	25%	27%	52%	56%
Model 1	2%	5%	31%	37%

*Birth weight was modelled on a log scale, which results in multiplicative effects.
¶ Reference category
‡ Age modelled by a piece-wise linear function: constant at reference range ≥20-30, and separate slopes for age <20, ≥30-40, and ≥40. For ages ≥40, the total estimated effect is hence addition of 10-year effect in age group ≥30-40 plus the corresponding effect in age-group ≥40.
† Percentage of regional variance explained by model predictors, i.e. percent reduction in variance of random effects (σ^2) when compared to model with no predictors (model 0).

Supplementary Table S4. Comparison of results from fully adjusted model without and with additionally including Swiss index of socio-economic position (SEP). Based on eligible population (*N* = 315,177); 277 days correspond to $39^{4/7}$ weeks.

	Model 3 without SEP		Model 3 with SEP	
	Gestational age (days) Absolute differences (95% CI)	Birth weight (g) * Relative differences (95% CI)	Gestational age (days) Absolute differences (95% CI)	Birth weight (g) * Relative differences (95% CI)
**Intercept**	277.3 (277.2 to 277.5)	3278 (3218 to 3339)&	277.3 (277.2 to 277.5)	3278 (3218 to 3339)&
**Sex**				
¶Female	0	1	0	1
Male	-0.56 (-0.65, -0.48)	1.045 (1.044 to 1.046)	-0.56 (-0.65, -0.48)	1.045 (1.044 to 1.046)
**Birth rank**				
1¶	0	1	0	1
2	-0.39 (-0.49, -0.29)	1.038 (1.037, 1.039)	-0.39 (-0.49, -0.29)	1.038 (1.037, 1.039)
3	-0.37 (-0.52, -0.22)	1.050 (1.048, 1.051)	-0.37 (-0.52, -0.22)	1.050 (1.048, 1.051)
≥ 4	-0.24 (-0.50, 0.02)	1.058 (1.056, 1.061)	-0.24 (-0.49, 0.02)	1.058 (1.056, 1.061)
**Age mother (yrs)‡**				
< 20 (per 5 yrs decr.)	-4.10 (-5.59, -2.61)	1.002 (0.987, 1.017)	-4.10 (-5.59, -2.61)	1.002 (0.987, 1.017)
20 - <30¶	0	1	0	1
≥ 30-40 (per 5 yrs)	-0.99 (-1.06, -0.91)	1.000 (1.000, 1.001)	-0.99 (-1.07, -0.92)	1.000 (1.000, 1.001)
≥ 40 (per 5 yrs)	-2.93 (-3.36, -2.50)	0.998 (0.994, 1.003)	-2.92 (-3.35, -2.50)	0.998 (0.994, 1.003)
**Civil status◊**				
Married	0	1	0	1
Not married	-0.01 (-0.13, 0.10)	0.990 (0.989, 0.991)	-0.01 (-0.13, 0.10)	0.990 (0.989, 0.991)
**Nationality mother**				
Switzerland¶	0	1	0	1
S Europe	0.20 (-0.01, 0.40)	0.994 (0.992, 0.996)	0.20 (0.00, 0.41)	0.994 (0.992, 0.996)
W Europe	0.20 (0.02, 0.38)	1.008 (1.006, 1.010)	0.20 (0.02, 0.37)	1.008 (1.006, 1.010)
N Europe	0.37 (-0.07, 0.81)	1.025 (1.020, 1.029)	0.36 (-0.08, 0.80)	1.025 (1.020, 1.029)
E Europe	0.21 (0.04, 0.38)	1.013 (1.011, 1.014)	0.22 (0.05, 0.39)	1.013 (1.011, 1.015)
Other	-0.32 (-0.49, -0.14)	1.007 (1.005, 1.008)	-0.31 (-0.48, -0.14)	1.007 (1.005, 1.008)
**Nationality father**				
Switzerland¶	0	1	0	1
S Europe	-0.46 (-0.64, -0.28)	0.991 (0.990, 0.993)	-0.45 (-0.63, -0.27)	0.992 (0.99, 0.993)
W Europe	0.07 (-0.11, 0.25)	1.008 (1.006, 1.009)	0.07 (-0.11, 0.25)	1.008 (1.006, 1.009)
N Europe	0.51 (0.08, 0.94)	1.013 (1.009, 1.017)	0.50 (0.07, 0.93)	1.013 (1.009, 1.017)
E Europe	-0.46 (-0.64, -0.28)	1.009 (1.007, 1.010)	-0.45 (-0.63, -0.27)	1.009 (1.007, 1.011)
Other	-0.02 (-0.22, 0.18)	0.992 (0.991, 0.994)	-0.01 (-0.21, 0.19)	0.993 (0.991, 0.995)
missing	-3.87 (-4.24, -3.50)	0.989 (0.985, 0.992)	-3.86 (-4.23, -3.49)	0.989 (0.985, 0.993)
**SEP index**				
1st quintile	-	-	-0.08 (-0.25, 0.08)	0.997 (0.996, 0.999)
2nd quintile	-	-	-0.09 (-0.24, 0.06)	0.998 (0.997, 1.000)
3rd quintile	-	-	-0.02 (-0.17, 0.13)	0.998 (0.997, 0.999)
4th quintile	-	-	0.02 (-0.12, 0.17)	1.000 (0.999, 1.002)
5th quintile¶	-	-	0	1
**Altitude (m)**				
500¶	0	1	0	1
per 500 m increase	0.07 (-0.09, 0.23)	0.989 (0.988, 0.991)	0.08 (-0.08, 0.23)	0.989 (0.988, 0.991)
**Urbanization**				
Urban¶	0	1	0	1
Peri-urban	-0.43 (-0.57, -0.28)	1.001 (1.000, 1.002)	-0.43 (-0.57, -0.28)	1.001 (1.000, 1.002)
Rural	-0.15 (-0.32, 0.02)	1.000 (0.998, 1.001)	-0.13 (-0.30, 0.04)	1.001 (0.999, 1.002)
**Language region**				
German¶	0	1	0	1
French	-0.62 (-0.77, -0.47)	0.989 (0.987, 0.990)	-0.61 (-0.76, -0.46)	0.989 (0.987, 0.990)
Italian	-0.94 (-1.26, -0.63)	0.982 (0.980, 0.985)	-0.93 (-1.24, -0.61)	0.982 (0.980, 0.985)
**% variation explained**				
Full model	31%	87%	31%	88%
Model without Lang. region	14%	66%	15%	68%

*Birth weight was modelled on a log scale, which results in multiplicative effects. The model for birth weight was additionally adjusted for gestational age by a cubic spline function with knots at weeks 25, 30 and 35.

& In the model for BW, the intercept corresponds to an estimated mean birth weight (g) for a singleton girl born at gestational age 40 weeks as the first child (rank 1) in a German-speaking, urban region of elevation 500m, whose mother is 20-30 years old at birth and married, and both parents have Swiss nationality and tertiary education.

¶ Reference category

‡ Age modelled by a piece-wise linear function: constant at reference range ≥20-30, and separate slopes for age <20, ≥30-40, and ≥40.

◊ Married or in registered partnership / Not married: Single, widow, divorced or in dissolved partnership

† Percentage of regional variance explained by model predictors, i.e. percent reduction in variance of random effects (σ^2) when compared to model with no predictors (model 0).

For peer review only - http://bmjopen.bmj.com/site/about/guidelines.xhtml

Supplementary Figure S1. Selection of eligible and complete case study populations among live births in Switzerland 2011 to 2014.

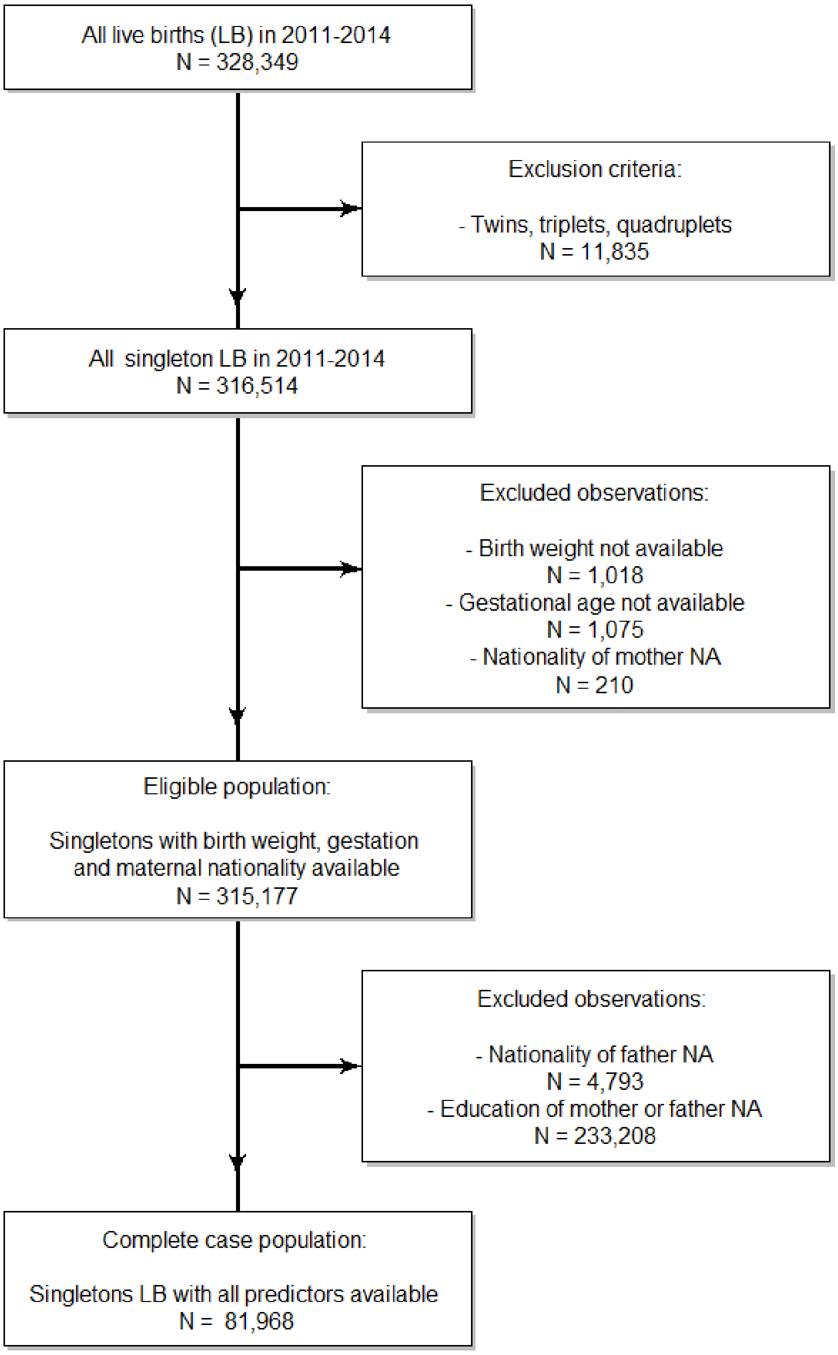

Supplementary Figure S2. Relationship between birth weight and gestational age at birth modelled by a cubic spline function. Separate fitted curves are shown for newborn girls and boys, with all other predictors corresponding to the reference categories shown in Table 2.

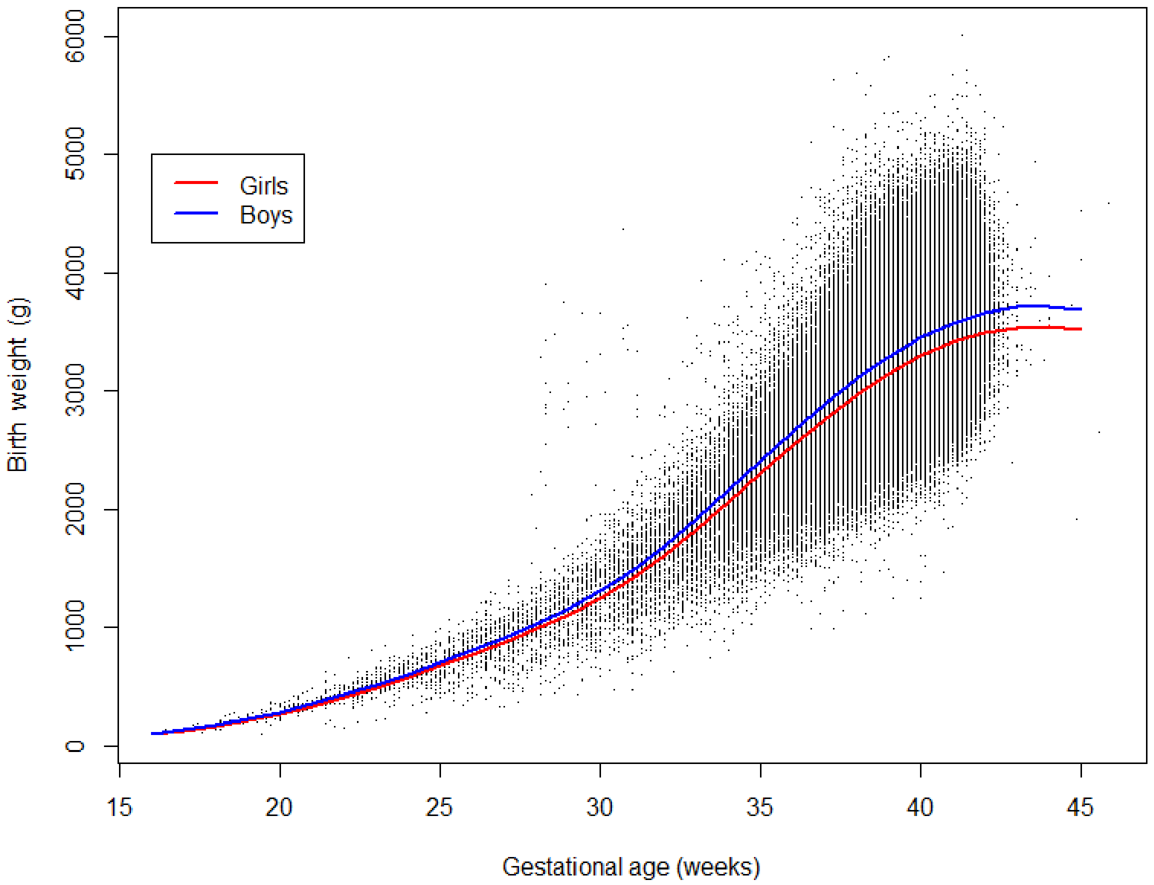

Supplementary Figure S3. Relationship between mean gestational age and proportion of preterm live births (<37 weeks) among eligible live births across 705 regions (upper panel) and between mean birth weight and proportion of low birth weight births (<2500g) (lower panel). Results from linear regression weighted by the number of live births in each region. Prediction intervals displayed for an average-size region (n=447). GA = gestational age; BW= birth weight; 276 days correspond to 39$^{3/7}$ weeks.

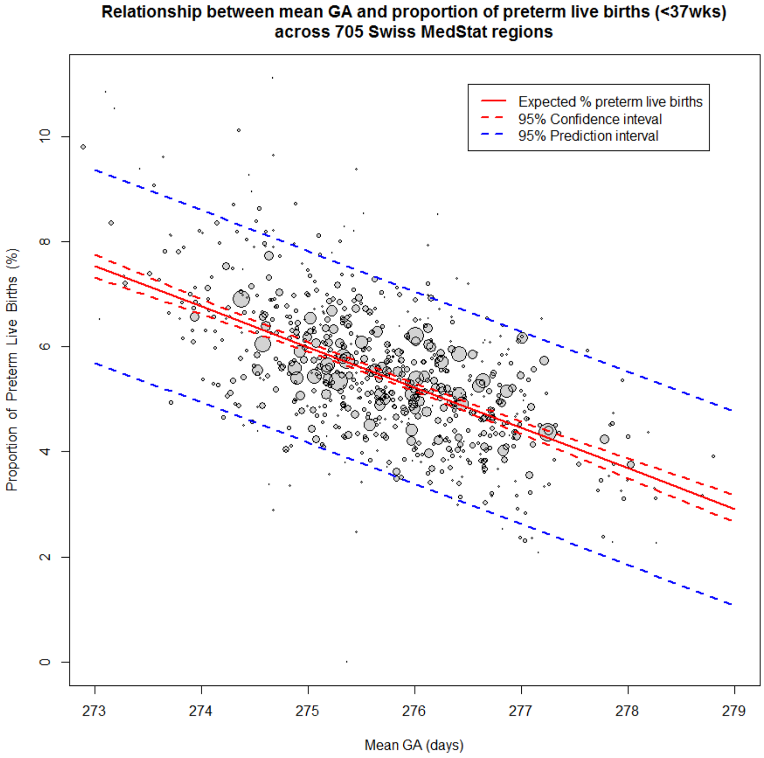

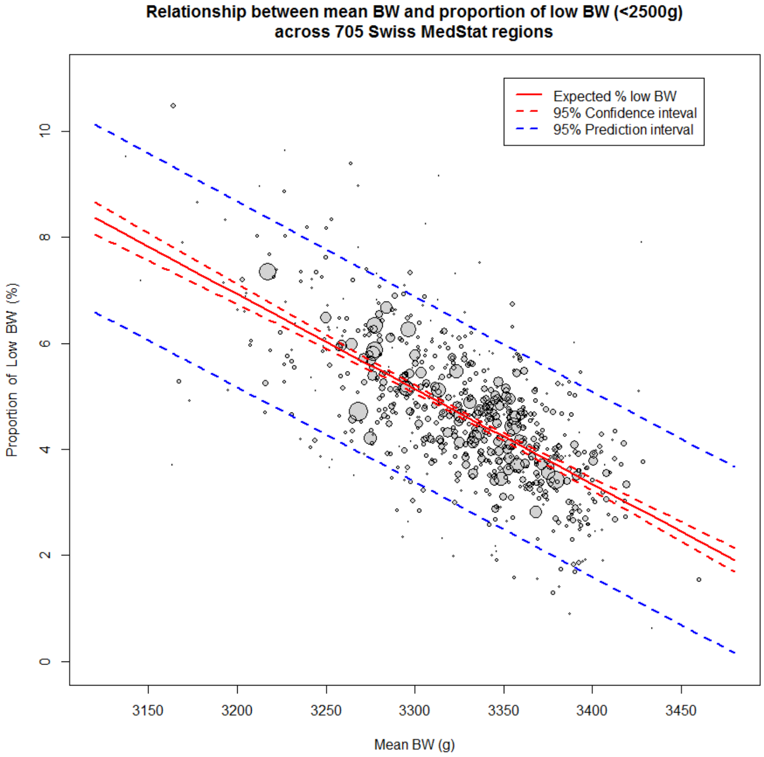

STROBE Statement—checklist of items that should be included in reports of observational studies

	Item No	Recommendation	Page No
**Title and abstract**	1	(*a*) Indicate the study's design with a commonly used term in the title or the abstract	1
		(*b*) Provide in the abstract an informative and balanced summary of what was done and what was found	2
**Introduction**			
Background/rationale	2	Explain the scientific background and rationale for the investigation being reported	4
Objectives	3	State specific objectives, including any prespecified hypotheses	4
**Methods**			
Study design	4	Present key elements of study design early in the paper	5
Setting	5	Describe the setting, locations, and relevant dates, including periods of recruitment, exposure, follow-up, and data collection	6
Participants	6	(*a*) *Cohort study*—Give the eligibility criteria, and the sources and methods of selection of participants. Describe methods of follow-up<br>*Case-control study*—Give the eligibility criteria, and the sources and methods of case ascertainment and control selection. Give the rationale for the choice of cases and controls<br>*Cross-sectional study*—Give the eligibility criteria, and the sources and methods of selection of participants	6
		(*b*) *Cohort study*—For matched studies, give matching criteria and number of exposed and unexposed<br>*Case-control study*—For matched studies, give matching criteria and the number of controls per case	
Variables	7	Clearly define all outcomes, exposures, predictors, potential confounders, and effect modifiers. Give diagnostic criteria, if applicable	5
Data sources/ measurement	8*	For each variable of interest, give sources of data and details of methods of assessment (measurement). Describe comparability of assessment methods if there is more than one group	5
Bias	9	Describe any efforts to address potential sources of bias	7
Study size	10	Explain how the study size was arrived at	8
Quantitative variables	11	Explain how quantitative variables were handled in the analyses. If applicable, describe which groupings were chosen and why	6
Statistical methods	12	(*a*) Describe all statistical methods, including those used to control for confounding	6
		(*b*) Describe any methods used to examine subgroups and interactions	na
		(*c*) Explain how missing data were addressed	8
		(*d*) *Cohort study*—If applicable, explain how loss to follow-up was addressed<br>*Case-control study*—If applicable, explain how matching of cases and controls was addressed<br>*Cross-sectional study*—If applicable, describe analytical methods taking account of sampling strategy	na
		(*e*) Describe any sensitivity analyses	7

Continued on next page

For peer review only - http://bmjopen.bmj.com/site/about/guidelines.xhtml

**Results**			
Participants	13*	(a) Report numbers of individuals at each stage of study—eg numbers potentially eligible, examined for eligibility, confirmed eligible, included in the study, completing follow-up, and analysed	8
		(b) Give reasons for non-participation at each stage	8
		(c) Consider use of a flow diagram	Figure S1
Descriptive data	14*	(a) Give characteristics of study participants (eg demographic, clinical, social) and information on exposures and potential confounders	Table 1, Table S1
		(b) Indicate number of participants with missing data for each variable of interest	Table 1
		(c) *Cohort study*—Summarise follow-up time (eg, average and total amount)	
Outcome data	15*	*Cohort study*—Report numbers of outcome events or summary measures over time	
		Case-control study—Report numbers in each exposure category, or summary measures of exposure	
		Cross-sectional study—Report numbers of outcome events or summary measures	Table 1
Main results	16	(*a*) Give unadjusted estimates and, if applicable, confounder-adjusted estimates and their precision (eg, 95% confidence interval). Make clear which confounders were adjusted for and why they were included	Table 2, Table S3
		(*b*) Report category boundaries when continuous variables were categorized	All tables
		(*c*) If relevant, consider translating estimates of relative risk into absolute risk for a meaningful time period	na
Other analyses	17	Report other analyses done—eg analyses of subgroups and interactions, and sensitivity analyses	Table S2, S3
**Discussion**			
Key results	18	Summarise key results with reference to study objectives	11
Limitations	19	Discuss limitations of the study, taking into account sources of potential bias or imprecision. Discuss both direction and magnitude of any potential bias	12
Interpretation	20	Give a cautious overall interpretation of results considering objectives, limitations, multiplicity of analyses, results from similar studies, and other relevant evidence	12
Generalisability	21	Discuss the generalisability (external validity) of the study results	12
**Other information**			
Funding	22	Give the source of funding and the role of the funders for the present study and, if applicable, for the original study on which the present article is based	29

*Give information separately for cases and controls in case-control studies and, if applicable, for exposed and unexposed groups in cohort and cross-sectional studies.

Note: An Explanation and Elaboration article discusses each checklist item and gives methodological background and published examples of transparent reporting. The STROBE checklist is best used in conjunction with this article (freely available on the Web sites of PLoS Medicine at http://www.plosmedicine.org/, Annals of Internal Medicine at

For peer review only

For peer review only - http://bmjopen.bmj.com/site/about/guidelines.xhtml

http://www.annals.org/, and Epidemiology at http://www.epidem.com/). Information on the STROBE Initiative is available at www.strobe-statement.org.

For peer review only

For peer review only - http://bmjopen.bmj.com/site/about/guidelines.xhtml
